# Structural Basis for Methine Excision by a Heme Oxygenase-like Enzyme

**DOI:** 10.1021/acscentsci.4c00015

**Published:** 2024-07-24

**Authors:** William C. Simke, Morgan E. Walker, Logan A. Calderone, Andrew T. Putz, Jon B. Patteson, Caitlin N. Vitro, Cynthia F. Zizola, Matthew R. Redinbo, Maria-Eirini Pandelia, Tyler L. Grove, Bo Li

**Affiliations:** † Department of Chemistry, The University of North Carolina at Chapel Hill, Chapel Hill, North Carolina 27599, United States; ‡ Department of Biochemistry, Brandeis University, 415 South Street, Waltham, Massachusetts 02453, United States; § Integrated Program for Biological and Genome Sciences, Department of Biochemistry and Biophysics, and Department of Microbiology, The University of North Carolina at Chapel Hill, Chapel Hill, North Carolina 27599, United States; ∥ Department of Biochemistry, Albert Einstein College of Medicine, Bronx, New York 10461, United States

## Abstract

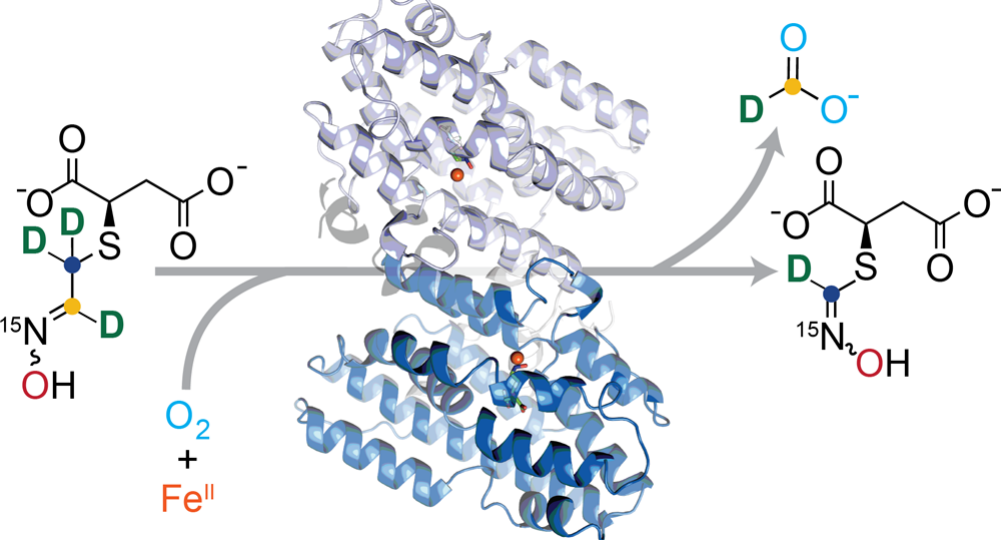

Heme oxygenase-like domain-containing oxidases (HDOs) are a rapidly expanding enzyme family that typically use dinuclear metal cofactors instead of heme. FlcD, an HDO from the opportunistic pathogen *Pseudomonas aeruginosa*, catalyzes the excision of an oxime carbon in the biosynthesis of the copper-containing antibiotic fluopsin C. We show that FlcD is a dioxygenase that catalyzes a four-electron oxidation. Crystal structures of FlcD reveal a mononuclear iron in the active site, which is coordinated by two histidines, one glutamate, and the oxime of the substrate. Enzyme activity, Mössbauer spectroscopy, and electron paramagnetic resonance spectroscopy analyses support the usage of a mononuclear iron cofactor. This cofactor resembles that of mononuclear non-heme iron-dependent enzymes and breaks the paradigm of dinuclear HDO cofactors. This study begins to illuminate the catalytic mechanism of methine excision and indicates convergent evolution of different lineages of mononuclear iron-dependent enzymes.

## Abstract 2

The heme oxygenase-like enzyme FlcD catalyzes an unusual four-electron oxidation and excision of a methine carbon using a non-heme mononuclear iron cofactor in the biosynthesis of fluopsin C.

## Introduction

Heme oxygenases are responsible for heme degradation in mammals, plants, and bacteria.^1^ These enzymes use heme both as a substrate and as a cofactor, catalyzing the oxidation of hemin to α-biliverdin, carbon monoxide, and free iron. They adopt a helical fold with a heme located between two of the seven conserved helices.^2−4^ The heme oxygenase fold is also found in enzymes that are not involved in heme degradation, such as PqqC in the biosynthesis of pyrroloquinolone quinone^5−7^ and TenA in the thiamine salvage pathway (Figure S1A).^8−10^ Despite having the heme oxygenase fold, PqqC and TenA do not require a metal cofactor to catalyze their reactions (Figure S1A).

Recently, a new family of heme oxygenase-like enzymes has emerged—the heme oxygenase-like diiron oxidases and oxygenases (HDOs). Several HDOs catalyze oxidative cleavage of carbon–carbon bonds (Figure S1B). For example, UndA catalyzes the oxidative decarboxylation of dodecanoic acid to the biofuel undecene,^11−13^ and BesC catalyzes the conversion of 4-chloro-lysine to 4-chloro-allylglycine in the biosynthesis of the terminal alkyne-containing amino acid β-ethynyl serine (Figure S1B).^14−16^Chlamydia protein associating with death domains (CADD) catalyzes the cleavage between the α- and β-carbon of its active site tyrosine and an amination to generate *para*-aminobenzoic acid (Figure S1C).^17−21^ HDOs also introduce essential functionalities into the structures of therapeutically and ecologically important natural products. For example, SznF catalyzes consecutive *N*-hydroxylations of methylarginine in the biosynthesis of the anticancer agent streptozotocin (Figure S1B).^22−24^ AetD catalyzes the oxidative rearrangement of a brominated tryptophan to a nitrile in the biosynthesis of the eagle-killing toxin aetokthonotoxin (Figure S1B).^25−27^ Because of the intriguing chemical reactions they catalyze and their key roles in the biosynthesis of important natural products, HDOs are of great interest for structural and mechanistic studies.

HDOs share a similar α-helical fold with heme oxygenases but use metal cofactors. Mössbauer spectroscopy, stopped-flow-absorption spectroscopy, and crystallography studies have established that UndA, SznF, BesC, and AetD all utilize a dinuclear iron cofactor;^12,13,15,16,23,24,27^ thus, “diiron” was included in the original naming of the protein family. Structural analysis of these HDOs revealed a conserved motif that is responsible for binding the dinuclear metal cofactor. However, activity of HDOs is not solely iron-dependent, as CADD appears to employ a dinuclear Mn/Fe cofactor for catalysis.^19−21^

**Figure 1:**
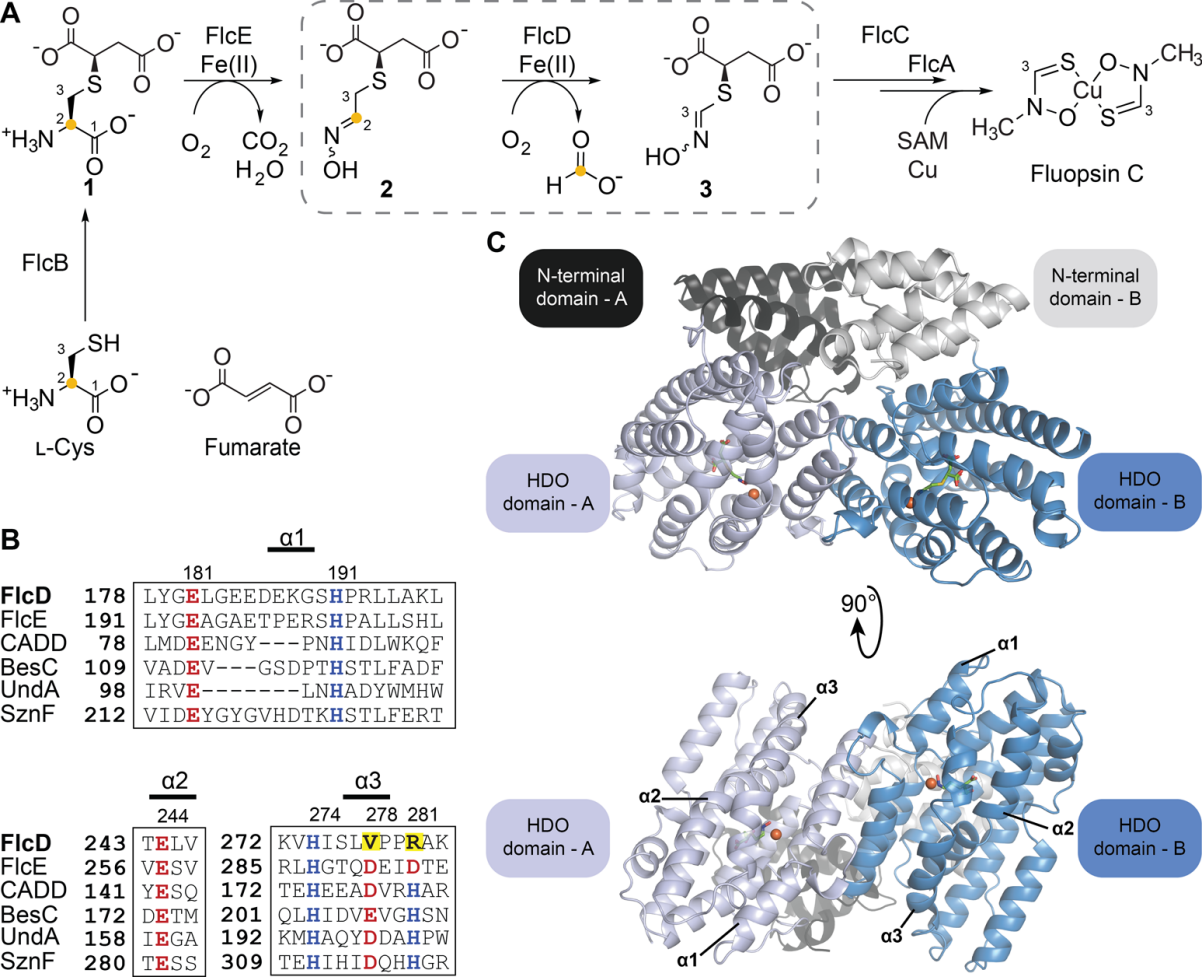
The sequence and structure of the carbon-excision enzyme FlcD. (A) Biosynthesis of fluopsin C. FlcD catalyzes the excision of the oxime carbon as formate, which originates from carbon-2 of l-cysteine (yellow dot). Carbons 1–3 are labeled. SAM, *S*-adenosyl methionine. (B) Sequence alignment of FlcD and FlcE to other characterized HDOs. The conserved metal-binding residues (aspartate/glutamate in red and histidine in blue) are located on three core helices (α1, α2, and α3). The last two metal ligands of typical HDOs are replaced by V278 and R281 in FlcD (yellow highlight). (C) Homodimeric structure of FlcD with bound Fe (orange) and substrate (green) in two views, one that shows both the N-terminal and the HDO domains (top) and the other with 90° rotation that focuses on the HDO domain (bottom). Monomer A (light blue/black) and monomer B (blue/light gray) each contain a single iron in the HDO domain. The core α-helices are labeled.

We discovered two new HDOs, FlcE and FlcD, from *Pseudomonas aeruginosa*, which are required for the biosynthesis of a rare copper-containing antibiotic, fluopsin C.^28^ FlcE and FlcD are responsible for oxidatively removing both carbon-1 and -2 from l-cysteine to generate the thiohydroxamate copper ligand (Figure 1A). Fluopsin C biosynthesis begins with the FlcB-catalyzed addition of l-cysteine and fumarate to form *S*-succinyl-l-cysteine (**1**). FlcE then catalyzes the oxidative decarboxylation and *N*-hydroxylation of **1** to form an oxime (**2**) and carbon dioxide. Subsequently, FlcD catalyzes the excision of the oxime carbon from **2** to generate a second oxime (**3**) and formic acid. Cleavage of the carbon–sulfur bond in **3** is catalyzed by FlcC followed by *N*-methylation catalyzed by FlcA using SAM as a cofactor, generating *N*-methylthiohydroxamate, two of which bind copper to form fluopsin C (Figure 1A). The ability to catalyze excision of nonaromatic carbons, as is the case for FlcD, has only been reported for a few other enzymes, such as the peptidyl-mercaptoglycine synthase TglHI^29−31^ and the 2-hydroxyethylphosphonate dioxygenase HEPD^32−34^ (Figure S1D). Thus, we sought to characterize FlcD both structurally and mechanistically.

Structural analysis of characterized HDOs revealed a conserved **E**-**H**-**E**-**H**-**(D/E)**-**H** motif that is responsible for binding dinuclear metal cofactors (Figure 1B), with a seventh residue (E) involved in iron binding in SznF.^24^ FlcD contains a divergent sequence in the conserved dimetal-binding motif in which the last two residues involved in coordinating the second iron are replaced with a valine and arginine to form an **E-H-E-H-V-R** motif (Figure 1B). An extant structure of *P. aeruginosa* FlcD (PDB: 3BJD), deposited by the Midwest Center for Structural Genomics, showed that FlcD exhibits the canonical seven-helix fold like other HDOs and contains a single nickel ion in the active site (Figure S2). The presence of nickel in the structure is likely an artifact since we previously showed that the activity of FlcD requires iron.^28^ Additionally, unlike the other HDOs, FlcD does not require external reductants for activity under multiple turnover conditions,^28^ suggesting that it couples a four-electron oxidation of the methine carbon to formate with the four-electron reduction of molecular oxygen (O_2_). This observation together with the deviant metal-binding motif and the presence of a single metal ion in the deposited structure raises the possibility that FlcD employs a mononuclear cofactor instead.

Here we sought to characterize the cofactor stoichiometry and mechanism of FlcD using structural, spectroscopic, and isotopic labeling studies. Our findings support the usage of a mononuclear iron cofactor by FlcD that is unique among HDOs characterized to date. Structures of FlcD bound to iron and substrate show that the oxime of the substrate coordinates to the mononuclear iron. Additionally, isotopic labeling reveals that FlcD is a dioxygenase incorporating both oxygens from O_2_ into formate. Lastly, by analyzing the primary sequences of ∼5,000 HDOs and predicting their metal-binding motifs, we found that the mononuclear iron-binding motif of FlcD constitutes a small fraction of the sequenced HDOs.

## Results

### Crystal Structures of FlcD Reveal the Presence of a Mononuclear Iron Cofactor

**Figure 2:**
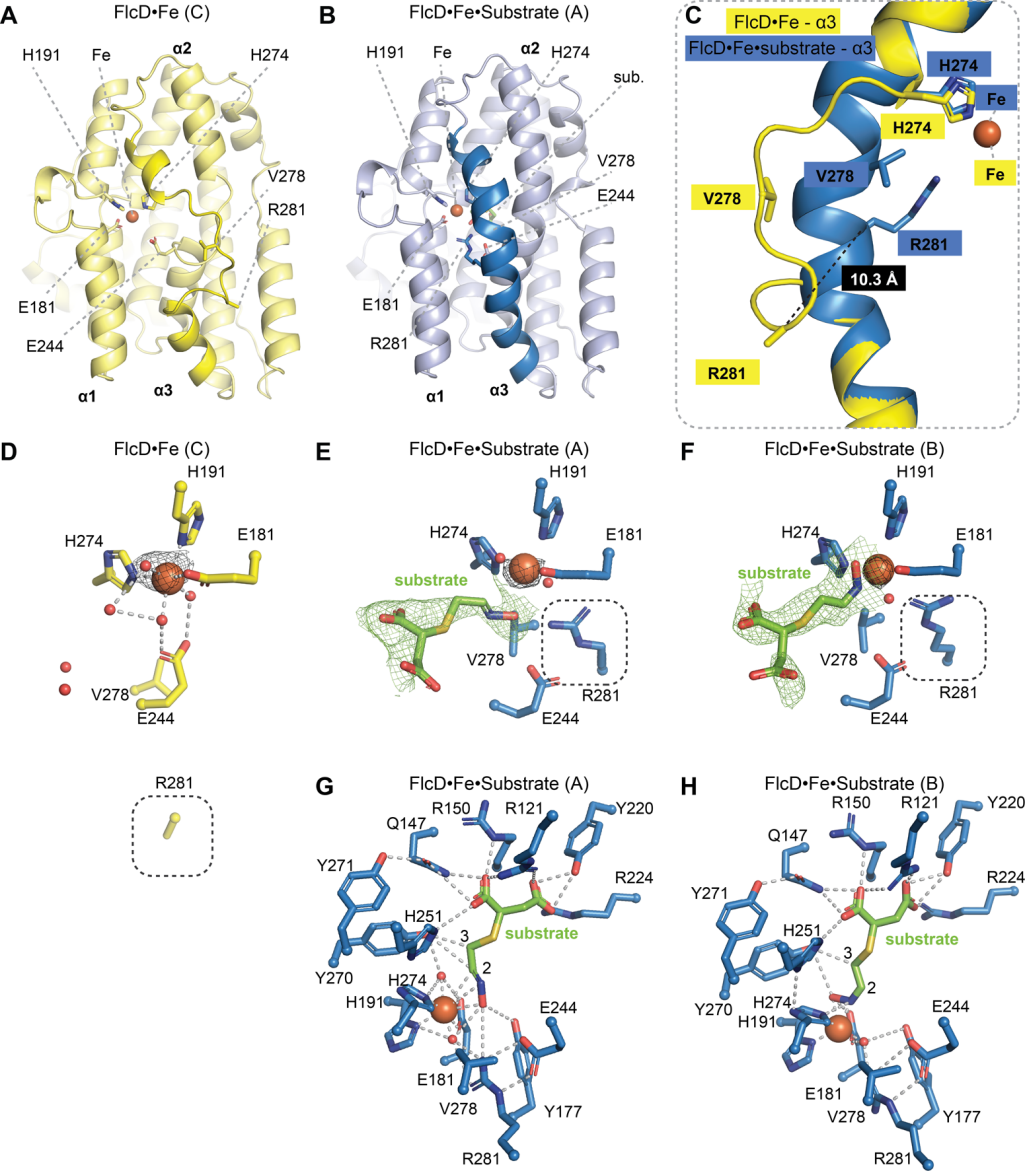
FlcD contains a single iron in each monomer and undergoes a conformational change upon substrate binding. (A) Structure of monomer C of FlcD bound to Fe (FlcD·Fe, PDB:9B9M), showing six residues that align with the metal-binding motif in other HDOs (yellow sticks), Fe (orange sphere), and α3 helix (bright yellow). This structure contains four monomers (two dimers) in the asymmetric unit. Monomers A, B, and D exhibit similar structures to monomer C. (B) Structure of monomer A of FlcD bound to Fe and substrate (FlcD·Fe·substrate, PDB: 9B9N), showing six residues that align with the metal-binding motif in other HDOs (light and dark blue sticks), substrate (green), Fe (orange), and the α3 helix (dark blue). (C) Overlayed structures of the α3 helix of the FlcD·Fe monomer C (yellow) and FlcD·Fe·substrate monomer A (blue). Fe (orange). The loop containing V278 and R281 forms a continuous α3 helix upon substrate binding, resulting in an ∼10 Å movement of the Cα of R281. (D–F) The active site of (D) FlcD·Fe monomer C, (E) FlcD·Fe·substrate monomer A, and (F) FlcD·Fe·substrate monomer B. Gray dashed boxes in (D–F) highlight the movement of R281 upon substrate binding. Side chain of R281 is not resolved in (D). Omit map of substrate is shown at 1.0 σ (green mesh). Fo-Fc map (red mesh) of substrate and iron anomalous signal (black mesh) are shown at 3.0 σ. (G, H) Active site of FlcD·Fe·substrate (G) monomer A and (H) monomer B. Key residues (sticks) include iron-binding and substrate-binding residues. Polar, metal–ligand, and charge–charge interactions are shown (gray dashes). Carbons 2 and 3 are labeled. Fe (orange), waters (red), substrate (green).

We obtained Fe-bound crystal structures of FlcD (FlcD·Fe) by growing FlcD crystals under anaerobic conditions in an MBraun glovebox maintained at 0.1 ppm of O_2_ and soaking the crystals in anoxic Fe(II)-containing buffer (Figure 2A, Figure S3, Table S1, PDB: 9B9M, 2.07 Å resolution) or by growing FlcD crystals outside the glovebox in the presence of excess iron (Figure S4, PDB: 8W1Q, 1.56 Å resolution). FlcD is a homodimer in each structure. Each monomer contains two domains: an N-terminal superhelical linker domain (IPR037061) and a C-terminal iron-binding domain (IPR016084, Figure S3). The iron-binding domain exhibits an overall seven-helix architecture characteristic of other HDOs (Figure 2A,B, Figures S4–S6).^11,16,17,24^ The interface of the FlcD homodimer exhibits extensive interactions between the monomers, including multiple hydrogen-bonding and π–π stacking interactions at the N-terminal domain and many electrostatic interactions at the HDO domain (Figures S7 and S8). Size exclusion chromatography coupled with multiangle light scattering analysis on FlcD without a His_6_-tag (FlcD_tagless_) confirmed that FlcD is a dimer in solution (Figure S9) and corroborates our structural data. Iron anomalous difference maps extracted from our native data revealed that in all cases each monomer contains a single iron situated in the middle of the three core helices (α1, α2, α3), which are surrounded by four auxiliary helices (Figure 2A, Figure S4, Table S1).

In the FlcD·Fe structure, Fe is coordinated by three residues that are part of the conserved iron-binding motif in other HDOs—E181 (α1), H191 (α1), and H274 (α3)—and three water molecules to form an octahedral geometry (Figure 2D). This coordination is reminiscent of the 2-His-1-Glu/Asp facial triad in non-heme iron α-ketoglutarate (α-KG)-dependent enzymes but exhibits meridional geometry (Figure S10). E244 is located on α2 and aligns with the fourth residue of the metal-binding motif of other HDOs and, instead of coordinating a second iron, is within hydrogen-bonding distance to two of the iron-coordinating water molecules (Figure 2D). V278 and R281, which replace the terminal two residues of the conserved metal-binding motif in other HDOs, are part of a loop that disrupts the α3 helix. A second iron is absent and the α2 helix is unfolded near the active site (Figure 2A).

The FlcD substrate, **2**, was synthesized using FlcE and **1** (Figures S11–S13). By soaking anaerobically grown crystals in both anoxic Fe(II) and substrate, we obtained a structure of FlcD with Fe and substrate bound (FlcD·Fe·substrate, PDB: 9B9N, 2.28 Å resolution) (Figure 1C, Table S1). The loop that contains V278 and R281 now folds into a continuous α3 helix (Figure 2B,C) and closes the active site alongside folding of the α2 helix (Figure S14), showing that substrate binding alone can induce conformational changes in FlcD without binding a second Fe. Surprisingly, upon substrate binding, the Cα of R281 moves 10.3 Å toward the active site (Figure 2C) to place the guanidino group within hydrogen-bonding distance to E244 (Figure 2E,F). The positions of the iron-binding residues, E181, H191, and H274, in both monomers of the FlcD·Fe·substrate structure are nearly identical; however, the substrate exhibits two distinct binding modes (Figure 2E,F). In monomer A, the oxime C=N–O of the substrate forms a complex with Fe and completes an octahedral geometry with two waters (Figure 2E, Figure S15). In monomer B, the oxime N–O conforms around the Fe in a square pyramidal geometry with one water (Figure 2F, Figure S16). To confirm that these distinct binding modes are not an artifact, we obtained a second FlcD·Fe·substrate structure from anaerobic crystals grown under different conditions (PDB: 9B9O, 2.16 Å resolution). This structure shows similar substrate binding modes to those from the first structure (Figure S17). In both monomers, the oxime moiety of the substrate coordinates the iron, while the succinate moiety interacts with the side chains of R121, Q147, R150, Y220, R224, and H251 in both monomers (Figure 2G,H, S18, S19A,B).

To identify which FlcD residues are essential for activity and probe their potential roles in catalysis, we generated alanine variants of five residues that correspond to the metal-binding motif of other HDOs—E181, H191, E244, H274, and R281—and nine residues in the vicinity of the substrate to alanine or phenylalanine (Table S3, Figure S20). Activity of the wild-type and variants was measured after a 2 min reaction with substrate to approximate the apparent initial velocity under pseudo-steady-state conditions (Figure S21). Formation of product was quantified by liquid chromatography–high resolution mass spectrometry (LC-HRMS) and normalized to that of the wild-type. While all variants except for Y271F resulted in nearly zero activity at 2 min, at longer incubation times (2 h) some of the variants exhibited some activity (Figure S19C,D). Alanine substitutions of the following residues resulted in no activity, independent of the conditions: E181, H191, and H274 (iron-binding residues), E244 and R281 (involved in the folding of the α3 helix upon substrate binding, Figure S19A,B), and R121 and R150 (substrate-interacting residues). The Q147A, Y177F, Y220F, R224A, and H251A variants showed some activity at 2 h, but their activities were 3–10-fold less than that of the wild-type, likely because these substitutions weaken the interactions with the substrate. Although Y270 does not appear to form polar contacts with the substrate, the Y270F variant exhibited no activity at 2 min and was over 10-fold less active than the wild-type at 2 h. Because the hydroxyl of Y270 lies ∼4 Å away from both carbon-2 and -3 of the substrate (Figure 2G,H, S19A,B), the loss in activity of Y270F suggests that Y270 plays a role in rate-limiting steps that may involve product release, which is corroborated by chemical quench data of Y270F (*vide infra*).

### Mössbauer and EPR Spectroscopies Corroborate a Mononuclear Iron Cofactor in FlcD

**Figure 3:**
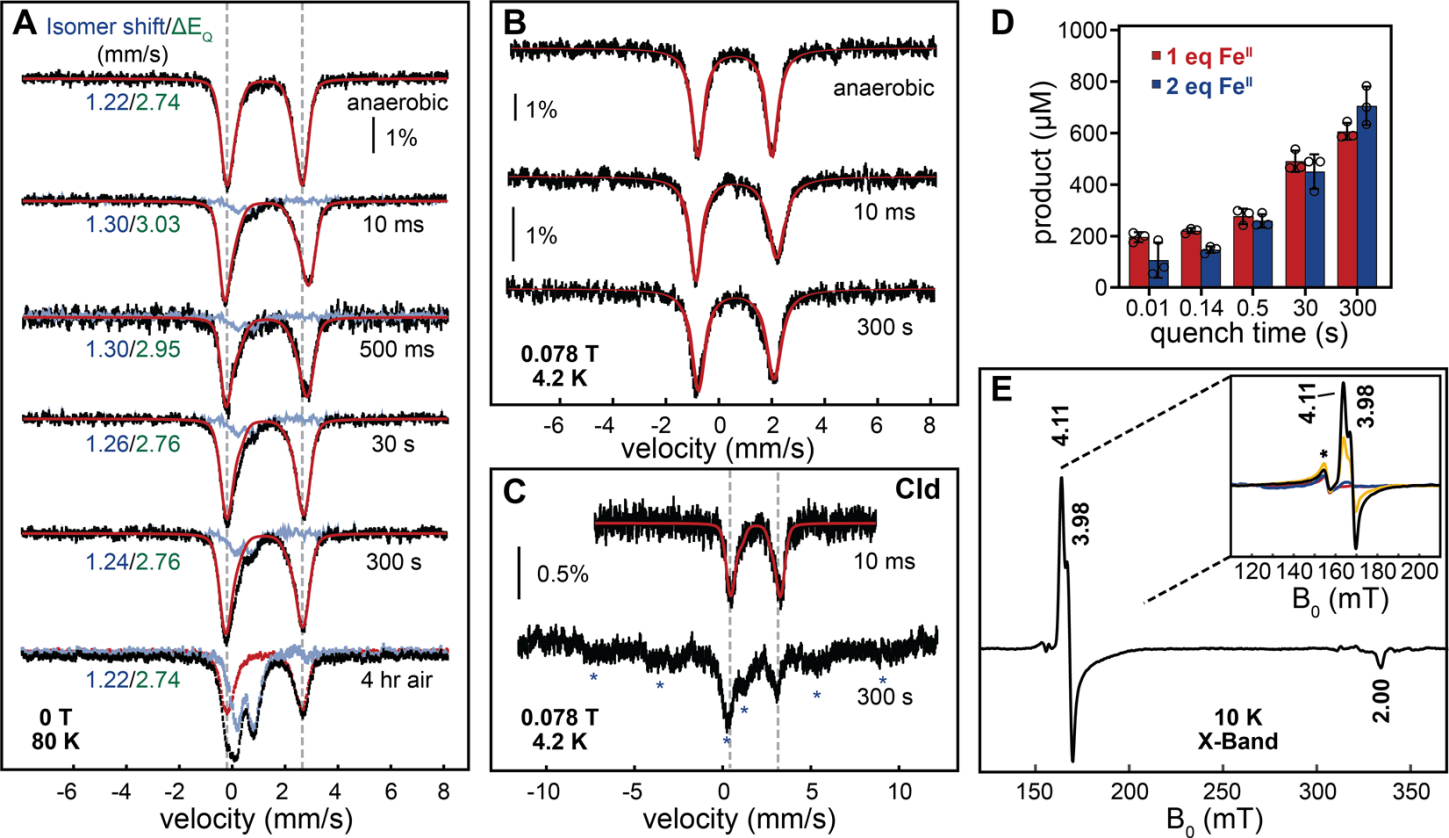
RFQ Mössbauer and EPR spectroscopic characterization of the reaction of FlcD·Fe(II)·substrate with O_2_ supports a mononuclear iron cofactor. (A) 80 K Mössbauer spectra of an anoxic solution of FlcD (1.7 mM) reconstituted with 1 molar equiv of Fe(II) and 3 molar equiv of substrate, as is or reacted with an O_2_-saturated buffer (1.8 mM) at 5 °C and quenched at selected time points. The spectra were acquired in the absence of an external magnetic field. The experimental spectra are shown as black vertical bars, the quadrupole doublets corresponding to the fit of the Fe(II) component are shown as red solid lines, and the isomer shift (dark blue) and Δ*E*_Q_ (green) are listed in each spectrum. Over time, a small amount of Fe(III) accumulates (blue trace) and its spectrum is obtained after subtraction of the ferrous quadrupole doublet fit from the experimental spectrum. (B) 4.2 K Mössbauer spectra of the same samples as in (A) and in the presence of a small external magnetic field (0.078 T) applied parallel to the direction of the γ-beam. (C) 4.2 K Mössbauer spectra of an anoxic solution of FlcD (1.7 mM) containing 1 molar equiv of Fe(II), 3 molar equiv of substrate, and 10 μM chlorite dismutase (Cld) reacted with a buffer solution containing 7.5 mM sodium chlorite. The experimental spectra are shown as black vertical bars; the quadrupole doublets corresponding to the fit of the Fe(II) component are shown as red solid lines. The asterisks in the 300 s spectrum highlight the positions of the mononuclear high-spin Fe(III) that accumulates in the reaction. (D) Time-dependent product formation in the reactions of FlcD reconstituted with 1 (red) or 2 (blue) molar equiv of Fe(II). Conditions are identical to those of the RFQ Mössbauer experiment with the exception of the Fe(II) molar equiv. Reactions were quenched by the addition of an equal volume of 3.5% H_2_SO_4_ (chemical quench). Product formation was quantified based on the absorbance peak at 250 nm from LC analysis. Product identity was confirmed using HRMS. E) CW normal mode EPR spectrum of FlcD (1.7 mM) reconstituted with 1 molar equiv of Fe(II) and 3 molar equiv of substrate reacted with an O_2_-saturated buffer (1.8 mM) at 5 °C for 300 s, after subtraction of the anaerobic control to remove any background signals due to adventitious high-spin Fe(III) (*g* ∼ 4.3). The inset contains time-dependent raw spectra centered on the low-field region that were obtained after reaction with O_2_ at time points 0 ms (red), 10 ms (blue), 45 s (yellow), and 300 s (black), respectively. The asterisk indicates mononuclear Fe(III). Experimental conditions: *T* = 10 K, microwave frequency 9.36 GHz, microwave power 2 mW, and modulation amplitude 1 mT.

Because the metal cofactors of HDOs are labile,^11−13,18,19,22−24^ we sought to verify our crystallographic findings and identify potential intermediate states of the FlcD iron center using rapid freeze quench (RFQ) Mössbauer, stopped-flow-absorption (SF-Abs), and continuous wave (CW) electron paramagnetic resonance (EPR) spectroscopies. The 80 K Mössbauer spectrum of FlcD reconstituted with ^57^Fe(II) and substrate under anoxic conditions shows a quadrupole doublet with an isomer shift (δ = 1.22 mm/s) and quadrupole splitting (Δ*E*_Q_ = 2.74 mm/s) characteristic of high-spin Fe(II) with N/O coordination (Figure 3A, Table S4). The same spectrum was obtained at 4.2 K and in the presence of a small external magnetic field (78 mT) with identical Mössbauer parameters (Figures 3B, S22). The FlcD·Fe(II)·substrate complex was subsequently mixed with O_2_-saturated buffer in a 1:1 ratio and rapidly frozen at various time points to allow for the detection of any accumulated intermediates. The Mössbauer spectrum of the 10 ms time point also shows a quadrupole doublet with parameters characteristic of high-spin Fe(II), albeit with parameters shifted slightly toward higher energies (δ = 1.30 mm/s, Δ*E*_Q_ = 3.03 mm/s) with respect to those observed for the O_2_-free FlcD·Fe(II)·substrate complex (Figure 3A, Table S4). This new Fe(II) species is transient, as demonstrated by the progressive downshift of the high-energy line yielding back the spectrum of the starting FlcD·Fe(II)·substrate complex (δ = 1.22 mm/s, Δ*E*_Q_ = 2.74 mm/s) at the end of the reaction. In addition, at later reaction time points, the Mössbauer spectra contain a second doublet that increases in intensity at ∼0.6 mm/s (corresponding to ∼15% of the Fe at 300 s) with parameters reminiscent of high-spin Fe(III).^35,36^ This species does not decay, demonstrating that it is not an intermediate in the reaction, but rather an oxidative product of the FlcD cofactor. This Fe(III) species must be paramagnetic because the 4.2 K spectra (slow relaxation regime) of the same samples completely lack the resonance at 0.6 mm/s (Figures 3B, S22). The apparent disappearance of this quadrupole doublet at 4.2 K is due to its conversion to a broad multiline spectrum that is masked by the baseline and consistent with the paramagnetism of the associated species. This Fe(III) component is thus incompatible with either an Fe_2_(III)-peroxo or an Fe_2_(III)-oxo species that are formally diamagnetic but is instead consistent with a half-integer mononuclear high-spin Fe(III) form that is paramagnetic.

To exclude the scenario that the lack of any detectable higher valence intermediates is due to limiting O_2_ concentration, we carried out the same experiment at low temperatures (4.2 K) but employed the chlorite dismutase (Cld)/chlorite system that yielded an almost 4-fold higher concentration of O_2_. The 4.2 K Mössbauer spectra (Figure 3C) show the same signal at 10 ms as that observed in the experiment using O_2_-saturated buffer (Figure 3B), but at 300 s a larger amount of mononuclear Fe(III) is discernible. Cumulatively, the Mössbauer data do not demonstrate formation of a diiron species at any point of the reaction, but only show accumulation of mononuclear Fe(III) species. Additionally, no optically detectable species reminiscent of an Fe_2_(III)-peroxo or Fe(IV)-oxo species were observed in SF-Abs experiments conducted with a protonated or dideuterated substrate ([3,3-D_2_]-**2**) (Figure S23), which was prepared by deuterium exchange of **2** in D_2_O (Figure S24A–C).

Although no high-valent intermediate species were observed, a second but transient Fe(II) signal appears with parameters distinct from those of the anoxic FlcD·Fe(II)·substrate complex (Figures 3A, 3B, S22). Because the RFQ Mössbauer experiment was performed under limiting O_2_ conditions, we posited that at the end of the time course, product formation would plateau, and the complex would return to its starting configuration. We thus hypothesized that the transient Fe(II) species may represent a step in the FlcD reaction that follows formation of a high-valent O_2_-adduct (which remains undetectable in our experiments). To test this hypothesis and confirm that the RFQ Mössbauer experiment monitors steps during FlcD catalysis, we quenched the reactant solutions from the RFQ syringes using 1.75% sulfuric acid at identical time points and quantified product formation (Figure 3D) based on LC analysis and a standard curve of purified product (Figures S25–S28). The data show that under single-turnover conditions, the ratio of product formed with respect to the O_2_ concentration is ∼0.8:1, demonstrating only a small unproductive uncoupling and in good agreement with the ∼15% accumulation of an oxidized species in our RFQ Mössbauer experiments. As the transient Fe(II) species disappears and the initial FlcD·Fe(II)·substrate complex recovers (Figure 3A, Table S4), product continues to form (Figure 3D), suggesting that the transient Fe(II) species is catalytically relevant. Addition of product or substrate to O_2_-free FlcD·Fe(II) does not yield any spectroscopic shifts (Figure S29). These data demonstrate that FlcD is highly active, even though we did not observe formation of any oxidized Fe-oxygen adducts. Because Fe has been shown to serve as a cosubstrate and enhance the kinetics and yield of Fe_2_(III)-peroxo species, we also performed RFQ Mössbauer in tandem with chemical quench experiments for FlcD reconstituted with 2 molar equiv of Fe(II). Again, no accumulation of an oxidized Fe_2_(III)-O_2_ adduct was observed (Figure S30), while product formation was comparable to that observed for FlcD reconstituted with 1 molar equiv of Fe(II) (Figure 3D). Chemical quench of the Y270F variant incubated with 1 equiv of Fe(II) yielded similar levels of product to the wild-type (Figure S31). In contrast, Y270F exhibits minimal activity under multiple turnover conditions (Figure S19C,D); thus, Y270 may be involved in steps preceding subsequent turnover such as product release.

We further analyzed the iron equivalency of FlcD by reconstituting FlcD_tagless_ with excess iron in the presence or absence of substrate and washing away the unbound iron. As quantified by a ferrozine assay, reconstituted FlcD_tagless_ in the presence of substrate retained 0.97 ± 0.01 molar equiv of iron and 0.88 ± 0.04 molar equiv in the absence of substrate (Figure S32A). Without iron reconstitution, FlcD_tagless_ as purified only contained 0.02 ± 0.01 molar equiv of iron. We measured the activity of FlcD_tagless_ as purified at the 2 min time point under multiple turnover conditions with the addition of varying molar equivalents of iron. Similar levels of product formation were observed in assays containing 1, 2, and 10 molar equiv of iron to FlcD_tagless_ (Figure S32B). A small amount of product formation was observed in the assay without iron addition, presumably due to residual FlcD-bound iron (Figure S32B). The activity data under both single- and multiple-turnover conditions together with our structural and spectroscopy data demonstrate that FlcD binds one Fe ion and not two, which is unlike the two-metal cofactors of prototypical HDOs.

Because Mössbauer may be insensitive to low amounts of any mononuclear Fe(III)-O_2_ adducts formed during turnover, we turned to EPR spectroscopy to examine formation of potential transient Fe(III) species. The EPR spectrum of the anoxic FlcD·Fe(II)·substrate lacks any signals in the high-spin region (with the exception of a small amount of adventitiously bound Fe(III), asterisk Figure 3E inset). Reaction of the complex with O_2_ results in accumulation of a signal that grows over time and shows maximal intensity at 300 s. Subtraction of the anoxic FlcD·Fe(II)·substrate spectrum from the raw spectrum at the 300 s time point yields an almost axial signal with a now well-defined *g*_II_ at *g* = 2.00 and the other two principal *g*-values at 4.11 and 3.98 (Figure 3E). This signal is best described by considering an *S* = 3/2 system with a small rhombicity *E*/*D* of ∼0.01, while the inverse temperature dependence (Figure S33) suggests that the axial zero-field splitting factor *D* has a positive value, and the observed transitions are from the ±1/2 manifold (Figure S34). The time dependence of this signal is inconsistent with an intermediate species, but rather suggests an oxidative product. This high-spin signal in FlcD is best described by an {FeNO}^7^ complex (Enemark–Feltham notation for a complex that contains 5 electrons on Fe(III) and 2 electrons on NO with a total of 7 electrons).^37^ This complex has been assigned to the species formed in other mononuclear non-heme Fe-dependent enzymes when they react with NO regardless of the presence of their substrates.^38−40^ The complex presumably forms because of the undesired reaction of the FlcD·Fe(II)·product complex with O_2_ and may represent the mononuclear Fe(III) species that is an oxidative byproduct observed in our Mössbauer data. This assignment is further supported by the recapturing of the same signal when the FlcD·Fe(II)·product complex is reacted with O_2_ under the same conditions (Figure S35).

Although a high-valent intermediate could not be trapped, FlcD forms a transient Fe(II) species during its reaction and regenerates the FlcD·Fe(II)·substrate complex in the absence of an auxiliary reducing system (Figure 3A, 3B). The recovery of the FlcD·Fe(II)·substrate complex at the end of a single turnover is consistent with results from experiments under multiple turnover conditions, which show that FlcD reconstituted with 0.8 equiv of iron can undergo ∼30 turnovers in air over a 3 h period in the absence of external reductants (Figure S36). The cyclic nature of catalysis distinguishes FlcD from the characterized HDOs, all of which require auxiliary reductants for multiple turnovers.^11−13,15,16,18−20,22,23^ Overall, the spectroscopic findings suggest that the FlcD cofactor deviates from that of prototypical HDOs and more resembles that of mononuclear non-heme Fe-dependent oxidative enzymes.

### FlcD Is a Dioxygenase

**Figure 4:**
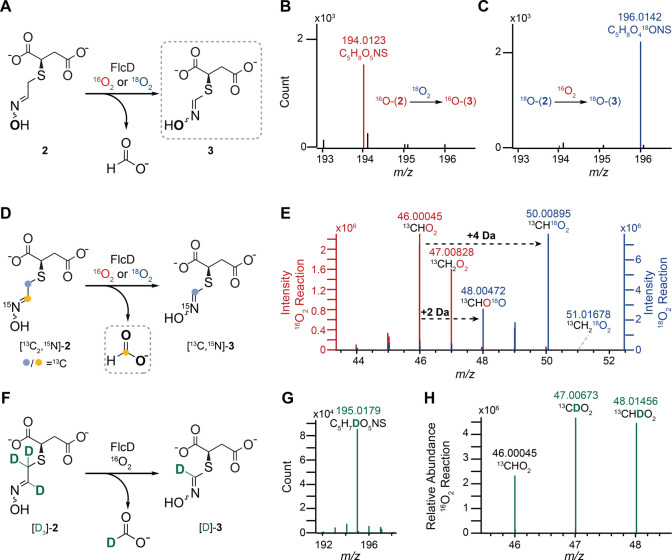
Oxygen and deuterium isotope tracing experiments demonstrate that FlcD is a dioxygenase that incorporates both oxygens of O_2_ into formic acid. (A) Reaction of FlcD in a ^16^O_2_ or ^18^O_2_ environment. (B, C) LC-HRMS spectra of the FlcD product, **3**, from a reaction using ^16^O-**2** in an ^18^O_2_ environment or using ^18^O-**2** in a ^16^O_2_ environment. (D) Reaction of [^13^C_2_,^15^N]-**2** in a ^16^O_2_ or ^18^O_2_ environment. (E) GC-HRMS spectra of formic acid produced in the FlcD reaction in a ^16^O_2_ (red) or ^18^O_2_ (blue) environment. Reaction in an ^18^O_2_ environment produced double ^18^O-labeled formic acid. (F) Reaction of FlcD using [2,3,3-D_3_]-**2** as substrate. (G) LC-HRMS spectrum of the FlcD product, **3**, showing that one deuterium is retained in **3** during the reaction (F). H) GC-HRMS spectrum shows enrichment of a deuterium in formic acid using [2,3,3-D_3_,^13^C_3_,^15^N]-**2** as a substrate for FlcD. LC-HRMS analysis was performed using electrospray ionization under positive ion mode. GC-HRMS analysis was performed using electron ionization under positive ion mode. All observed *m*/*z* from GC-HRMS analysis is within 1 ppm error of the calculated *m*/*z*.

To date, all characterized HDOs couple the reduction of O_2_ to oxidation of their substrates (Figure S1B,C).^11,14,18,22,26^ To determine the fate of O_2_ in the FlcD reaction, we performed isotope tracing experiments using either ^16^O_2_ or ^18^O_2_ (Figure 4A–4C). First, LC-HRMS analysis confirmed that no product was formed in the reaction under an anoxic environment (Figure S37), signifying the requirement of O_2_ in the FlcD reaction. Next, FlcD was reacted with unlabeled substrate, **2**, in an ^18^O_2_ environment. No ^18^O was incorporated into the product, **3** (Figure 4B). We also generated ^18^O-labeled **2** ([^18^O]-**2**) by conducting the FlcE reaction in an ^18^O_2_ environment, which led to incorporation of ^18^O into the oxime hydroxyl (Figures S38 and S39). Using [^18^O]-**2** as a substrate, the FlcD-catalyzed reaction retained the ^18^O label in the product ([^18^O]-**3**) regardless of whether the reaction was conducted in a ^16^O_2_ or ^18^O_2_ environment (Figures 4C and S40). These results demonstrate that the oxygen in the oxime is retained during the FlcD reaction and does not originate from water.

We also traced potential incorporation of O_2_ into the formic acid coproduct. ^13^C- and ^15^N-labeled substrate, [^13^C_2_,^15^N]-**2**, was used to distinguish formic acid produced in the FlcD reaction from spurious formic acid. [^13^C_2_,^15^N]-**2** was produced using FlcB, FlcE, and [^13^C_3_,^15^N]-l-cysteine (Figure S41). As expected, the FlcE-catalyzed reaction removed the carboxylate ^13^C of [^13^C_3_,^15^N]-**1**, preserving the remaining labels in [^13^C_2_,^15^N]-**2**. LC-HRMS analyses showed the FlcD-catalyzed reaction removed another ^13^C from [^13^C_2_,^15^N]-**2**, generating [^13^C,^15^N]-**3** (Figure S41). NMR analysis of this reaction at a larger scale confirmed production of [^13^C,^15^N]-**3** and ^13^C-formic acid (Figure S42), consistent with the removal of the ^13^C at position 2 as formate.^28^ The [^13^C]-formic acid produced in reactions conducted in ^16^O_2_ or ^18^O_2_ was analyzed using gas chromatography–high resolution mass spectrometry (GC-HRMS) (Figures 4D, 4E, and S43). Because the oxygen of formic acid rapidly exchanges with water under acidic conditions,^32^ the FlcD reaction of [^13^C_2_,^15^N]-**2** was conducted under ^18^O_2_ for 4 min. The major peak of [^13^C]-formic acid exhibited a mass increase of +4 Da, compared to formic acid produced in a ^16^O_2_ environment (Figure 4E), indicating that both oxygens from ^18^O_2_ are incorporated into formic acid. Smaller amounts of singly labeled (+2 Da) and unlabeled formic acid were also observed, likely due to exchange with ^16^O water. Consistent with this proposal, longer incubation (10 min) of the FlcD reaction resulted in higher levels of the singly ^18^O-labeled and unlabeled species (Figures S44 and S45). The ^18^O labeling data, together with the cyclic nature of the FlcD reaction shown by spectroscopy, demonstrate that FlcD is a dioxygenase that incorporates both oxygens from O_2_ into formic acid.

### Isotope Tracking of Substrate Protons

We further examined the fate of substrate protons in the FlcD reaction, with a focus on the protons that come from cysteine. Deuterated isotopologues of **2** were biosynthesized from [2,3,3-D_3_]-l-cysteine or [D_3_,^13^C_3_,^15^N]-l-cysteine (Figures S46A, S47A) and fumarate using FlcB and FlcE. FlcB catalyzed the formation of deuterated *S*-succinyl-l-cysteine ([D_3_]-**1** or [D_3_,^13^C_3_,^15^N]-**1**) (Figures S46B, S47B). All three deuteriums of [D_3_]-**1** or [D_3_,^13^C_3_,^15^N]-**1** were retained in the FlcE reaction, generating trideuterated **2** ([D_3_]-**2** or [D_3_,^13^C_2_,^15^N]-**2**) (Figures S46C, S47C). To track deuterium incorporation into the product, we incubated [D_3_]-**2** with FlcD in the presence of Fe(II) for 6 h. LC-HRMS analysis revealed that a single deuterium was retained in **3** ([D]**-3**), indicating the loss of two deuteriums from [D_3_]-**2** (Figures 4F, 4G, S47D). We then used the dideuterated **2** ([3,3-D_2_]-**2**) to determine the fate of protons on carbon 3 (Figure S24A–C). A single deuterium from [3,3-D_2_]-**2** was also retained in [D]-**3** (Figure S24D). These results show that the FlcD reaction retains one hydrogen and removes the other from carbon 3. We also measured the apparent kinetic isotope effect on carbon 3 in a competition assay. [3,3-D_2_]-**2** was mixed with unlabeled **2** in equal concentrations, and the FlcD-catalyzed formation of D-**3** and **3** was monitored over time by LC-HRMS (Figure S48A). No kinetic isotope effect was observed at each time point (Figure S48B), suggesting hydrogen abstraction at carbon 3 is not rate-limiting under the assay conditions.

Deuterium incorporation from [D_3_,^13^C_3_,^15^N]-**2** into formic acid was traced in the FlcD reaction using GC-HRMS, which can distinguish isotopologues (Figure S49). Ions that result from deuterated formic acid (^13^CDO_2_ and ^13^CHDO_2_) were significantly enriched compared to an unlabeled formic acid control (Figures 4H, S50, S51A,B). The levels of ions that correspond to protonated formic acid (^13^CH_2_O_2_) in the FlcD reaction with [D_3_,^13^C_3_,^15^N]-**2** were nearly identical to those in a deuterated sodium formate control (Figure S51C–E), suggesting that the protonated species result from background exchange with water. Overall, these results demonstrate that one deuterium from [D_3_]-**2** or [D_3_,^13^C_3_,^15^N]-**2** is removed from carbon 3, one is retained in the product, and the deuterium on carbon 2 is incorporated into formic acid.

### Bioinformatic Predictions of the Metal-Binding Motifs of Uncharacterized HDOs

The deviations in the conserved metal-binding motif and novel mononuclear iron cofactor in FlcD led us to examine whether divergent motifs are present in other members of the HDO family (Pfam14518). To predict the metal-binding motif of the uncharacterized HDOs, we analyzed the 150 largest groups in our previously generated sequence similarity network of HDOs.^28^ These groups account for approximately 95% of the sequences in the network. The HDO domains of all sequences from the 50 largest groups were trimmed and aligned with FlcD, FlcE, UndA, BesC, SznF, and CADD as references (Figure S52) to predict potential metal-binding motifs. These alignments showed that the first two residues varied in the spacing of their primary sequence, the third residue was spatially distinct, and the final three residues were close in sequence with variable spacing between them. This observation is consistent with the location of the first two residues on the α1 helix, the third residue on the α2 helix, and the last three residues on the α3 helix. Since multiple residues need to be on the same face of a helix to engage in metal binding, we generated possible motifs for the first two and last three metal-binding residues and predicted the most likely motif for each group based on its frequency (Figures S53–S59). Approximately 85% of the analyzed groups possessed the E-H-X-H-D-H or E-H-X-H-E-H motif found in UndA, CADD, SznF, and BesC (Figure S60). About 9% of the groups did not have strong predictions for the metal-binding motifs used in this search, likely due to the variable lengths of sequence between the possible metal-binding residues. These groups may also use different metal binding motifs or not bind a metal at all. About 4% of the groups are predicted to contain an asparagine in the last three residues. The FlcD motif (E-H-X-H-V-R) is only strongly predicted in 1 out of 150 groups (the FlcD subgroup), suggesting that this metal-binding motif is unique to the FlcD subgroup. Furthermore, a consensus sequence of the FlcD subgroup indicates the (E-H-X-H-V-R) motif, substrate-binding residues, and residues at the HDO dimer interface are highly conserved among members of the FlcD subgroup (Figure S61).

## Discussion

Crystallographic studies revealed that FlcD is an unusual mononuclear iron enzyme in the HDO family that typically use a dimetal cofactor. The structures of both SznF and CADD contain a dinuclear iron cofactor in their HDO domain,^17,24^ although CADD was recently found to also depend on Mn^2+^ for activity.^19,20^ The mononuclear iron in FlcD is positioned at the same site as the Fe1 in SznF and UndA and is coordinated by one aspartate and two histidines that are conserved among HDOs. Binding of the FlcD substrate and the subsequent relocation of R281 appear to sterically occlude another iron from binding to FlcD at a similar location to the Fe2 site of SznF (Figure S62). In turn, E244, which corresponds to the glutamate that bridges Fe1 and Fe2 in SznF, forms two hydrogen bonds with R281 instead of binding the metallocofactor. Although E244 and R281 do not form contacts with the mononuclear iron, they are both essential for activity and help close the active site pocket upon substrate binding (Figures S14, S62). These structural findings support the conclusion that FlcD is a mononuclear iron-dependent HDO.

The structure of FlcD exhibits conformational flexibility in both the α2 and α3 helices (Figure 2A–C). A flexible α3 helix was also reported for UndA, BesC, and SznF;^11,12,16,24^ however, assembly of the metallocofactor is sufficient for folding of the α3 helix in SznF,^24^ whereas α3 folding in FlcD requires binding of both mononuclear iron and substrate. Additionally, substrate binding to FlcD·Fe also induces folding of the α2 helix, while the α2 helix in SznF and UndA remains folded regardless of the iron-binding state. These conformational differences further distinguish FlcD from the characterized HDOs.

The dimeric state and substrate-binding modes of FlcD hint at the catalytic mechanism. FlcD, like SznF,^22,24^ contains an N-terminal superhelical linker domain and exists as a homodimer in crystal and in solution. UndA, BesC, CADD, and AetD lack an N-terminal domain but still form homodimers in crystal (Figure S6).^11,16,17,27^ The dimer interface between the HDO domains of FlcD is more similar to that of SznF, BesC, or CADD than that of UndA or AetD (Figure S6). In each FlcD monomer, the oxime of the substrate replaces one iron-bound water to coordinate iron (Figures S15–S17), consistent with the strong iron-binding properties of oximes.^41^ Interestingly, the oxime of the FlcD substrate binds iron in two different conformations, although it is unclear which monomer represents the productive substrate-binding mode. It remains to be seen if monomer-dependent substrate-binding modes also apply to SznF and other HDOs.

The mononuclear iron center in FlcD is unexpected but supported by our current spectroscopic and biochemical data. The data show that the active site configuration and mechanism of FlcD deviate from those of the archetypical representatives of the HDO family. Although UndA was originally reported to contain a mononuclear iron cofactor due to cofactor instability,^11^ all HDOs characterized to date apart from CADD employ a μ-peroxo-diiron(III) center as a common intermediate to initiate their oxidative chemical transformations.^12,13,15,16,23^ In contrast, reaction of the FlcD·Fe(II)·substrate complex with molecular oxygen neither forms a μ-peroxo-diiron(III) intermediate detectable by transient SF-Abs or RFQ Mössbauer spectroscopy nor yields any paramagnetic Fe(II)–Fe(III) diiron centers in RFQ EPR experiments. Only a mononuclear ferric species appears within a much shorter time scale than the species corresponding to the decomposition of the diferric cofactor in BesC, SznF, and UndA. This ferric species is best described as the oxidized form of the mononuclear FlcD cofactor. It is paramagnetic and was identified by EPR spectroscopy to be an Fe-nitroso center with an *S* = 3/2 spin state. Similar EPR signals have been observed in mononuclear non-heme Fe-dependent enzymes upon reaction with nitric oxide (NO) in the presence or absence of their substrates and assigned to {FeNO}^7^ complexes.^38−40^ In all these enzymes, catalysis proceeds through a ferryl-oxo intermediate and reaction with NO is used to capture any earlier O_2_-binding steps. In the case of FlcD, formation of this complex could result from tautomerization of the oxime product to a nitroso, which may be facilitated by the metallocofactor.

Despite the employment of high concentrations of molecular oxygen, Fe(II), or a dideuterated substrate, a ferryl intermediate in FlcD was not detected by either SF-Abs or RFQ Mössbauer experiments, suggesting that its accumulation is not favored. This situation closely resembles that of the mononuclear Fe/α-KG-dependent oxygenase, ethylene-forming enzyme (EFE), in which the ferryl species was hardly detectable, and only substitution of a residue proximal to the active site allowed for its enhanced accumulation.^42^ Similar substitution of a residue near the active site may be required to allow for accumulation and detection of a ferryl intermediate in FlcD.

**Figure 5:**
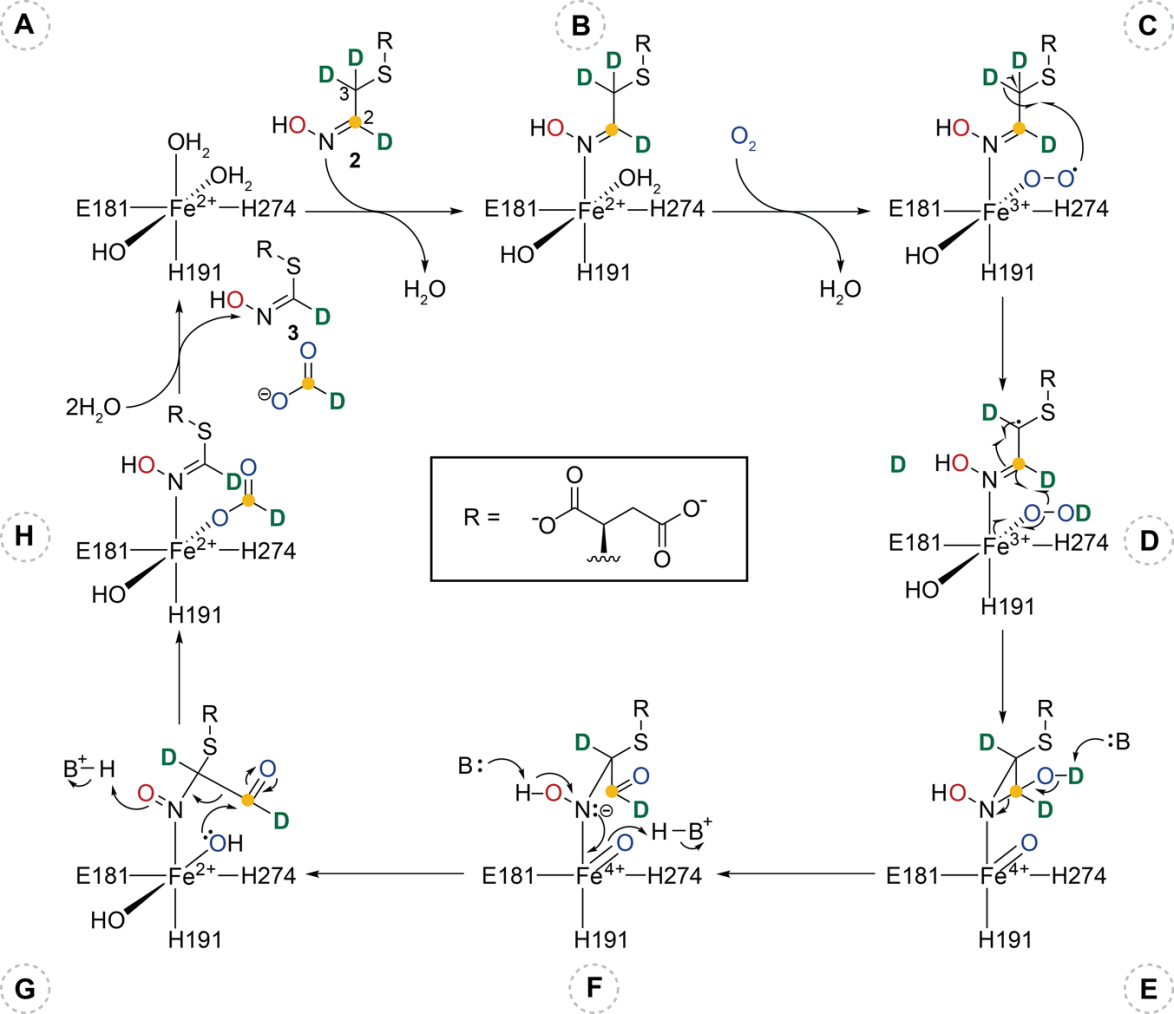
Proposed mechanism for oxidative cleavage catalyzed by FlcD. Fe is coordinated by E181, H191, and H274 and three waters. Relevant substrate atoms are labeled: carbon 2 of cysteine (yellow dot), the oxime oxygen (red), O_2_ (blue), and deuterium (green). FlcD begins with a mononuclear Fe(II) cofactor (**A**). Substrate replaces one of the water molecules and coordinates to Fe in a monodentate fashion (**B**). O_2_ is activated as Fe(III)-superoxo (**C**), which abstracts a deuterium from carbon 3, generating a radical (**D**). Hydroxylation at carbon 2 results in formation of an Fe(IV)-oxo, and radical recombination leads to formation of a hydroxy aziridine ring (**E**). Ring opening of the hydroxy aziridine results in an aldehyde (**F**). Two-electron oxidation of the hydroxylamine by Fe(IV)-oxo generates the nitroso and Fe(II) (**G**). Fe(II)-hydroxyl is transferred to the aldehyde. Isomerization of the nitroso and elimination of carbon 2 lead to formation of the oxime product, **3**, and formic acid (**H**). Products are released, completing the catalytic cycle.

The time-dependent RFQ Mössbauer spectra show formation of a transient ferrous species, with increased isomer shifts and quadrupole splitting values, which returns to the initial FlcD·Fe(II)·substrate complex after all molecular oxygen is consumed (Figure 3A). Similar spectral shifts have been observed in other mononuclear non-heme-Fe(II) enzymes and indicate conversion of a five-coordinate square-pyramidal Fe(II) to a six-coordinate Fe(II) center.^35^ This catalytically relevant Fe(II) intermediate may represent state H or G in the proposed mechanism (although state G may not favorably accumulate to be detected) (Figure 5). The amount of product in these single-turnover experiments is similar irrespective of whether FlcD is reconstituted with 1 or 2 molar equivalents of Fe(II) (Figure 3D), further supporting the assignment of the cofactor as mononuclear.

At the end of the reaction, FlcD returns to the initial ferrous state, ready to engage in a subsequent turnover. This result is corroborated by bulk activity assays in which FlcD catalyzes multiple turnovers in the absence of a reducing system (i.e., ascorbate). The regeneration of the ferrous state is characteristic of mononuclear non-heme Fe-dependent oxidases,^43,44^ but unlike that of other characterized HDOs, in which the diferric cofactor that forms following turnover needs to be reduced by an auxiliary reducing system. For instance, SznF, UndA, and BesC all require a reducing agent to catalyze multiple turnovers, while CADD only performs a single turnover as a suicide enzyme.^18−21^ Although the Fe cofactor in FlcD is labile and the protein is isolated in its apo form, Fe does not appear to be a cosubstrate, as higher levels do not enhance formation of intermediates or product, signifying another difference with the prototypical HDOs. Cumulatively, our spectroscopic and biochemical findings show that FlcD employs a mononuclear non-heme Fe cofactor that breaks the paradigm of a dinuclear active site for the HDO family. Thus, we propose to revise the HDO descriptor to heme oxygenase-like domain-containing oxidases.

Based on isotopic labeling and crystallographic data, we propose a mechanism for the oxidative cleavage catalyzed by the dioxygenase FlcD. The catalytic cycle begins with the coordination of Fe(II) with E181, H191, H274, and three waters (Figure 5A). Substrate binding displaces one water (Figure 5B), and O_2_ is activated by Fe(II), forming a Fe(III)-superoxo (Figure 5C), which abstracts a hydrogen atom from carbon 3 to generate a carbon radical (Figure 5D). Transfer of the hydroxyl radical from Fe(III)-hydroperoxo to the oxime carbon generates a Fe(IV)-oxo. Radical recombination leads to formation of a hydroxy aziridine (Figure 5E). The hydroxy aziridine ring opens to become an aldehyde and a hydroxylamine (Figure 5F). Two-electron oxidation of the hydroxylamine by Fe(IV)-oxo results in formation of a nitroso and Fe(II) hydroxide (Figure 5G). Transfer of the hydroxide from Fe(II) to the aldehyde generates a carboxylic acid, which is eliminated as formic acid, while the nitroso isomerizes to the oxime, **3** (Figure 5H). Both products are then released, and FlcD returns to its initial state for another turnover. Aziridine formation has precedent in natural product biosynthesis. For example, formation of an aziridine product from l-valine was reported for the Fe/α-KG enzyme TqaL in the biosynthesis of 2-aminoisobutyric acid.^45,46^ An aziridinium intermediate was also proposed for the Fe/α-KG enzyme DmfD that catalyzes methyl group insertion to synthesize dehydrofosmidomycin (Figure S1E).^47^ Although the lack of a deuterium kinetic isotope effect appears to contradict our proposed mechanism in which H atom abstraction on carbon 3 is an irreversible step, this observation could be due to a preceding or subsequent step being rate-limiting under the assay conditions. Other mechanisms for the oxidative cleavage by FlcD are also possible, including one that involves formation of a hydroxy aziridine carbon radical before the rebound of the hydroxyl from Fe(III)-hydroperoxo (Figure S63). Alternatively, radical addition on the oxime could generate an Fe(III)-peroxo-substrate-bridged species before H atom abstraction. Subsequent radical migration could lead to oxidation of carbon 2 without invoking a hydroxy aziridine intermediate (Figure S64).

The mononuclear iron cofactor of FlcD and the four-electron oxidation chemistry it catalyzes are reminiscent of mononuclear non-heme iron-dependent oxidases,^44^ such as cysteine dioxygenase (CDO),^48−50^ sulfoxide inserting enzyme (EgtB),^51−53^ isopenicillin N synthase (IPNS) (Figure S65),^54−56^ and HEPD^32^ (Figure S1D). Both CDO and EgtB catalyze four-electron oxidation of cysteine sulfur (Figure S65A and S65B), whereas we demonstrate that FlcD is a dioxygenase of a methine carbon. IPNS catalyzes the cyclization of the β-lactam and thiazolidine rings in isopenicillin (Figure S65C), which involves hydrogen abstraction at a carbon adjacent to sulfur by a ferric superoxo intermediate.^55^ FlcD catalysis also requires H atom abstraction at a carbon adjacent to sulfur, although the lack of deuterium isotope effects suggests that H atom abstraction may not be rate-limiting. The species that abstracts the H atom remains to be determined. While these enzymes all catalyze four-electron oxidations and share a 3-His or 2-His-1-Asp coordination of a mononuclear iron, they adopt different folds and likely descend from different evolutionary lineages, including the cupin domain in CDO and HEPD,^32,49^ the DinB-like domain in EgtB,^51,57^ and now the heme oxygenase-like domain in FlcD. Over evolution, these different folds have converged on non-heme mononuclear iron centers to catalyze diverse chemistry.

Our results indicate that FlcD is a remarkable mononuclear iron enzyme in the heme oxygenase-like superfamily. The mononuclear iron cofactor is distinct from other characterized members of the superfamily, which currently include dinuclear Fe (SznF, UndA, BesC, and AetD),^12,13,15,16,23,24,27^ dinuclear Fe/Mn or Mn/Mn (CADD),^19−21^ and no metal cofactor (PqqC).^5,7^ As in the case of FlcD, deviations in iron-binding motifs may be used to predict the cofactor nuclearity in heme oxygenase-like enzymes. The low frequency and high conservation of the mononuclear iron-binding motif of the FlcD subgroup (∼1% of all groups) suggest that mononuclear iron-dependent enzymes constitute a small fraction of the sequenced HDOs. Investigation of HDOs with undetermined motifs or those predicted to use unusual metal-binding residues will likely uncover new enzymatic functions and chemistries.

## Conclusions

The heme oxygenase-like enzyme FlcD is a carbon dioxygenase. FlcD catalyzes excision of the oxime carbon and incorporates both oxygens from molecular oxygen into the coproduct formic acid. We reveal the structural basis for substrate binding and catalysis and show FlcD shares the overall structural fold and conformational flexibility of the other dimetal HDOs. In contrast to quintessential HDOs, our data indicate that FlcD employs a mononuclear iron cofactor to catalyze a four-electron oxidation. These findings shed light on the mechanism of oxidative cleavage and carbon excision catalyzed by FlcD. Further spectroscopic and crystallographic studies are needed to characterize the mononuclear iron species. Our discovery also expands the cofactor diversity of the heme oxygenase-like superfamily to include mononuclear iron and will aid in understanding the evolution of non-heme mononuclear and dinuclear iron-dependent enzymes.

## Supporting Information Available

Materials, experimental procedures, tables, and supplemental figures for protein and small-molecule purity, crystallography, mass spectrometry, NMR, Mössbauer, EPR, and bioinformatics data (PDF)
