# Supplementary material for: Structural Basis for Methine Excision by a Heme Oxygenase-like Enzyme
Source: ACS Cent Sci. 2024 Jul 24;10(8):1524–36. doi: 10.1021/acscentsci.4c00015 (PMC11363339; doi:10.1021/acscentsci.4c00015)
Supplement: Supplementary file 1 — oc4c00015_si_001.pdf [file oc4c00015_si_001.pdf]

**Structural basis for methine excision by a heme oxygenase-like enzyme**

William C. Simke,[1#] Morgan E. Walker,[1#] Logan A. Calderone,[2#] Andrew T. Putz,[1] Jon B. Patteson,[1] Caitlin N. Vitro,[1] Cynthia F. Zizola,[4] Matthew R. Redinbo,[1,3] Maria-Eirini Pandelia,[2*] Tyler L. Grove,[4*] Bo Li[1*]

[1]Department of Chemistry, The University of North Carolina at Chapel Hill, Chapel Hill, NC 27599, USA

[2]Department of Biochemistry, Brandeis University, 415 South Street, Waltham, MA 02453, USA

[3]Integrated Program for Biological and Genome Sciences, Department of Biochemistry and Biophysics, and Department of Microbiology, University of North Carolina at Chapel Hill, Chapel Hill, NC 27599, USA

[4]Department of Biochemistry, Albert Einstein College of Medicine, Bronx, NY 10461, USA

[#]These authors contributed equally to this work

*Corresponding authors:

mepandelia@brandeis.edu

tyler.grove@einsteinmed.edu

boli@email.unc.edu

## Supporting Information


Media, culture conditions, and chemicals ........ S-1
General method for liquid chromatography-mass spectrometry analysis ........ S-1
General method for gas chromatography-mass spectrometry analysis ........ S-1
Expression and purification of FlcB ........ S-1
Expression and purification of FlcE ........ S-2
Expression and purification of FlcD ........ S-2
Purification of anaerobic FlcD ........ S-2
Expression and purification of TEV protease ........ S-2
$His_6$-TEV-FlcD cleavage of $His_6$-tag with TEV protease ........ S-3
Size exclusion chromatography multi-angle light scattering (SEC-MALS) analysis ........ S-3
Enzymatic synthesis and purification of FlcD substrate, **2** ........ S-4
Enzymatic synthesis and purification of FlcD product, **3** ........ S-4
Standard curve of FlcD product, **3** ........ S-5
Activity of FlcD in a 30-min time course ........ S-5
Crystallography ........ S-5
Cloning of FlcD mutants ........ S-6
Activity measurement of FlcD variants ........ S-6
EPR and Mössbauer spectroscopy ........ S-6
Preparation of rapid freeze-quench (RFQ) EPR and Mössbauer samples ........ S-7
Preparation of acid quenched samples ........ S-7
Stopped-flow absorption spectroscopy (SF-Abs) ........ S-7
Anaerobic preparation of $FlcD_{tagless}$ for determining iron equivalence ........ S-7
Ferrozine assay for iron equivalence testing of FlcD ........ S-8
Measuring FlcD activity in the presence of varying iron equivalents ........ S-8
Isotope tracing of [$^{13}C_3$, $^{15}N$]-Cys in the FlcE and FlcD reactions ........ S-9
Isotope tracing of [2,3,3-$D_3$]-Cys in the FlcE and FlcD reactions ........ S-9
Isotope tracing of oxygen in the one-pot FlcE and FlcD reactions ........ S-9
Isotope tracing of oxygen in the FlcD reaction ........ S-10
Isotope tracing of oxygen in formic acid using [$^{13}C_2$, $^{15}N$]-**2** in the FlcD reaction ........ S-10
Tracing deuterium in the FlcD reaction using deuterated **2** ........ S-11

NMR analysis of FlcD reaction to calculate turnovers ........ S-12
Sequence alignment of FlcD and FlcE to other characterized HDOs ........ S-12
Bioinformatic prediction of iron-binding motifs in HDOs (PFAM14518)........ S-12
Supplemental Tables: ........ S-13
Table S1. X-ray crystallography data collection and refinement statistics. ........ S-13
Table S2. Individual B-factors of iron and substrate ligands. ........ S-14
Table S3. Primers used in this study. ........ S-15
Table S4. Average Mössbauer parameters. ........ S-16
Supplemental Figures: ........ S-17
Figure S1. Reactions discussed in main text. ........ S-17
Figure S2. FlcD structure bound to nickel. ........ S-18
Figure S3. Tertiary and quaternary structure of FlcD•Fe. ........ S-19
Figure S4. Aerobic FlcD•Fe structure obtained by growing the crystal in Fe(II) ........ S-20
Figure S5. Comparison of the FlcD•Fe•substrate structure with other HDOs ........ S-21
Figure S6. Characterized HDOs crystalize as dimers. ........ S-22
Figure S7. The N-terminal domains of FlcD•Fe monomers exhibit electrostatic and hydrophobic interactions with each other. ........ S-23
Figure S8. The HDO domains of FlcD•Fe monomers exhibit extensive interactions with each other. S-24
Figure S9. FlcD is a stable homodimer. ........ S-25
Figure S10. Iron coordination geometry of FlcD is distinct from that of Fe/α-ketoglutarate-dependent enzymes. ........ S-26
Figure S11. LC-HRMS analysis of the purity of the FlcD substrate, **2**. ........ S-27
Figure S12. Representative $^{1}$H NMR spectrum of purified **2** in $D_6$-DMSO (850 MHz). ........ S-28
Figure S13. Representative $^{13}$C NMR spectrum of purified **2** in $D_6$-DMSO (850 MHz). ........ S-29
Figure S14. Active site pocket of FlcD closes upon substrate binding. ........ S-30
Figure S15. The substrate replaces waters in monomer C of the FlcD•Fe active site. ........ S-31
Figure S16. The substrate replaces waters in monomer D of the FlcD•Fe active site. ........ S-32
Figure S17. Two crystals of FlcD•Fe•substrate exhibit similar substrate binding modes. ........ S-33
Figure S18. FlcD•Fe•substrate Fo-Fc, OMIT, and anomalous maps. ........ S-34
Figure S19. Active site schematic of the FlcD•Fe•substrate structure and identification of essential residues for enzyme activity. ........ S-35
Figure S20. Purity of FlcD WT and variants assessed by sodium dodecyl sulfate-polyacrylamide gel electrophoresis (SDS-PAGE). ........ S-36
Figure S21. Time course of FlcD activity over 30 min. ........ S-37

Figure S22. The 4.2 K Mössbauer spectra of FlcD ........ S-38
Figure S23. FlcD single mixing stopped-flow-absorption spectroscopy experiments. ........ S-39
Figure S24. Preparation of [$D_2$]-**2** and its modification by FlcD. ........ S-40
Figure S25. LC-HRMS analysis of the purity of the FlcD product, **3**. ........ S-41
Figure S26. $^1$H NMR spectrum of purified **3** in $D_2O$ (850 MHz). ........ S-42
Figure S27. $^1$H NMR spectrum of 1 mM purified **3** in $D_2O$ with 1 mM of benzoic acid as an internal standard (850 MHz). ........ S-43
Figure S28. Standard curve of FlcD product, **3**. ........ S-44
Figure S29. The 80 K Mössbauer spectra of anoxic FlcD ........ S-45
Figure S30. The 80 K Mössbauer spectra of the FlcD reaction. ........ S-46
Figure S31. Time-dependent product formation in reactions of the Y270F variant reconstituted with one molar eq of Fe(II). ........ S-47
Figure S32. Iron content analysis of FlcD supports the presence of a mononuclear iron cofactor. ........ S-48
Figure S33. CW EPR spectra of anoxic FlcD. ........ S-49
Figure S34. Rhombogram for an S = 3/2 system with a positive axial zero-field splitting value D. ........ S-50
Figure S35. CW EPR spectra of FlcD•Fe(II)•substrate and FlcD•Fe(II)•product reacted in the absence and presence of $O_2$. ........ S-51
Figure S36. $^1$H NMR quantification of product formation in FlcD reactions containing 0.8 Fe(II) equivalents. ........ S-52
Figure S37. The FlcD reaction requires $O_2$. ........ S-53
Figure S38. FlcE incorporates one oxygen from $O_2$ into the oxime hydroxyl. ........ S-54
Figure S39. FlcE incorporates oxygen from $O_2$ into the oxime hydroxyl. ........ S-55
Figure S40. The FlcD-catalyzed reaction retains the oxime oxygen. ........ S-56
Figure S41. Biosynthesis of [$^{13}C$, $^{15}N$]-labeled metabolites in the fluopsin C pathway. ........ S-57
Figure S42. $^{13}C$ NMR verifies production of formic acid in the reaction of FlcD. ........ S-58
Figure S43. GC-HRMS analysis showed that formic acid produced by the reaction of FlcD possesses the same retention time as a formic acid standard. ........ S-59
Figure S44. Representative GC mass spectrum of formic acid produced in a reaction where [$^{13}C_2$, $^{15}N$]-**2** was incubated with FlcD in an $^{18}O_2$ environment for 10 min. ........ S-60
Figure S45. GC-HRMS quantification of labeled formic acid. ........ S-61
Figure S46. Biosynthesis of [$D_3$]-labeled metabolites in the fluopsin C pathway. ........ S-62
Figure S47. Biosynthesis of [$^{13}C$, $^{15}N$, $D_3$]-labeled metabolites in the fluopsin C pathway. ........ S-63
Figure S48. The reaction of FlcD did not exhibit an apparent kinetic isotope effect (KIE) toward the dideuterated substrate, $D_2$-**2**. ........ S-64
Figure S49. GC-HRMS can differentiate ions with high accuracy. ........ S-65

Figure S50. Mass spectrum of a formic acid standard. ........ S-66

Figure S51. GC-HRMS analysis of formate produced in the FlcD reaction containing [$^{13}C_2$, $^{15}N$, $D_3$]-**2**. ... S-67

Figure S52. NCBI accession IDs of HDOs used in bioinformatic analysis. ........ S-68

Figure S53. Bioinformatic approach to identify potential iron-binding motif of uncharacterized HDOs. S-69

Figure S54. Histogram analysis of potential iron-binding motif for SSN group 15 containing FlcD and FlcE. ........ S-70

Figure S55. Histogram analysis of potential iron-binding motif for SSN group 65 containing BesC. ... S-71

Figure S56. Histogram analysis of potential iron-binding motif for SSN group 1 containing uncharacterized HDOs. ........ S-72

Figure S57. Histogram analysis of potential iron-binding motif for SSN group 120 containing uncharacterized HDOs. ........ S-73

Figure S58. Heatmap of the predicted first two iron-binding residues of HDOs. ........ S-74

Figure S59. Heatmap prediction of the last three iron-binding residues of HDOs. ........ S-75

Figure S60. Pie chart of the predicted first two and last three iron-binding residues for the first 150 groups of HDOs in the SSN. ........ S-76

Figure S61. Consensus sequence of the FlcD subgroup shows the iron- and substrate- binding residues are conserved. ........ S-77

Figure S62. Comparison of the active site of FlcD•Fe•substrate to other characterized HDOs. ........ S-78

Figure S63. Alternative proposed mechanism of FlcD. ........ S-79

Figure S64. Alternative proposed mechanism of FlcD. ........ S-80

Figure S65. Reactions catalyzed by other mononuclear non-heme iron-dependent oxidases. ........ S-81

References ........ S-81

## Materials and Methods:

### Media, culture conditions, and chemicals

Chemicals were purchased from commercial suppliers and used without further purification. Diethyl ether was purchased from VWR. QIAquick PCR purification kit was purchased from Qiagen. Zymoclean Gel DNA Recovery Kit was purchased from Zymo Research. L-Cysteine, sodium ascorbate, 3-(2-pyridyl)-5,6-diphenyl-1,2,4-triazine-p,p′-disulfonic acid monosodium salt hydrate (ferrozine), 2-mercaptoethanol, $^{57}Fe$ and formic acid were purchased from Millipore-Sigma. HEPES, Luria broth (LB), acetonitrile (ACN), acetonitrile containing 0.1% formic acid, methanol, agar, glycerol, sodium chloride (NaCl), nickel(II) chloride hexahydrate, imidazole, bicine, potassium phosphate dibasic ($K_2HPO_4$), potassium phosphate monobasic ($KH_2PO_4$), isopropylthio-D-galactoside (IPTG), trifluoroacetic acid (TFA), hydrochloric acid, sulfuric acid ($H_2SO_4$), acetic acid, GeneJET Plasmid Miniprep Kit, and ampicillin were purchased from Fisher Scientific. Ammonium iron(II) sulfate hexahydrate ($(NH_4)_2Fe(SO_4)_2$ . $6H_2O$) and sodium fumarate were purchased from Acros Organics. DMSO-$D_6$, $D_2O$, sodium D-formate, [2,3,3-$D_3$]-L-cysteine, [2,3,3-$D_3$, $^{13}C_3$,$^{15}N$]-L-cysteine, $^{18}O_2$, and [$^{13}C_3$,$^{15}N$]-L-cysteine were purchased from Cambridge Isotopes Laboratories. Molecular biology reagents, such as restriction enzymes, were purchased from New England Biolabs. Crystallography screens MSCG1-4 were purchased from Anatrace.

### General method for liquid chromatography-mass spectrometry analysis

Compound analysis by liquid chromatography-coupled high resolution mass spectrometry (LC-HRMS, LC-MS for short) was conducted using Agilent Technologies 6520 Accurate-Mass Quadruple-Time of Flight (Q-TOF) mass spectrometer coupled with 1210 high-performance liquid chromatography (HPLC) as described.[1] MS analysis was performed using electrospray ionization under positive ion mode with the following parameters: gas temperature 300 °C, drying gas 10 L/min, nebulizer 45 lb/in$^2$, fragmentor 175 V, and skimmer 65 V. Samples were injected into a Kinetex C18 column (Phenomenex, 150 mm length, 2.6 μm particle size and 100 Å pore size) and separated using the following method at a flow rate of 0.4 mL/min. Solvent A contained 0.1% formic acid in water, and solvent B contained 0.1% formic acid in acetonitrile. Mobile phase was held at 2% B for 2 min, increased to 98% B over 16 min, and held at 98% B for 2 min before returning to 2% B over 1 min.

### General method for gas chromatography-mass spectrometry analysis

Samples were analyzed using a ThermoFisher Exactive gas chromatography (GC) mass spectrometer with an electron ionization source. Samples were acquired in positive ion mode with a scan range of 40−600 *m/z* and a resolution of 60,000. The ion source was set to 280 °C and the MS transfer line was set to 250 °C. The automatic gain control (AGC) target was set to $1\times10^6$. GC separation was performed using a TraceGOLD TG-5SilMS column (Thermo Scientific). Injection volume for samples was 2 μL. GC oven was initialized at 40 °C. At 1 min the GC oven started ramping at a rate of 25 °C/min until 300 °C over 11.5 min. The temperature was held at 300 °C for 2.5 min.

### Expression and purification of FlcB

The pLIC-His$_6$-TEV-FlcB (PA3516) was cloned previously.[1] BL21 (DE3) cells were transformed with the plasmid and protein expression was performed in 1 L of LB containing 100 μg/mL ampicillin with shaking at 225 rpm at 37 °C. Cells were grown to an optical density at 600 nm ($OD_{600}$) of 0.2–1.5 and expression was induced with the addition of 0.5 mM isopropyl β-D-1-

thiogalactopyranoside (IPTG). The temperature was then reduced to 16 °C and the cultures were grown overnight. $OD_{600}$ of cells was recorded, and cells were harvested by centrifugation at 4500 rcf for 30 min at 4 °C. The cell pellet was resuspended in ~20 mL wash buffer per 1 L of culture. Resuspended cells were kept on ice and sonicated at 30% amplitude for 2.5 min with a 1 s on and 1 s off cycle using a Sonic Dismembrator (Fisher Scientific model 500). Cellular debris was removed by centrifugation at 15000 rcf for 40 min at 4 °C. Cell lysate was clarified using a 0.45 μm filter. $His_6$-tagged FlcB was then purified by nickel affinity chromatography on an AKTA Pure FPLC or on benchtop using a 5 mL HisTrap column (GE). The wash buffer contained 25 mM bicine pH 8.0, 150 mM NaCl, 10% glycerol (v/v), and 30 mM imidazole. The elution buffer contained 25 mM bicine (or potassium phosphate) pH 8.0, 150 mM NaCl, 10% glycerol (v/v), and 500 mM imidazole. The storage buffer contained 25 mM bicine (or potassium phosphate) pH 8.0, 150 mM NaCl, and 10% (v/v) glycerol. Fractions of purified FlcB were verified by analysis on a 12% sodium dodecyl sulfate-polyacrylamide gel electrophoresis (SDS-PAGE) gel (195 V for 45 min) and stained with Coomassie blue (1.25 g/L in 50% methanol, 10% glacial acetic acid). Pure fractions were concentrated to 2.5 mL using an Amicon centrifugal filter with a 30 KDa cutoff (Millipore) and imidazole was removed from the protein using a PD-10 buffer exchange column. Purified protein was concentrated to 100−1000 μM and stored in pellets at −80 °C. Protein concentration was determined using Nanodrop based on a calculated extinction coefficient of 43430 $M^{-1}cm^{-1}$.

**Expression and purification of FlcE**

The pLIC-$His_6$-TEV-FlcE (PA3519) was cloned previously.[1] Expression and purification of $His_6$-tagged FlcE was performed using the same procedure as FlcB. Nickel affinity chromatography buffers included wash buffer (25 mM potassium phosphate or HEPES pH 7.0, 150 mM NaCl, and 30 mM imidazole), elution buffer (25 mM potassium phosphate or HEPES pH 7.0, 150 mM NaCl, and 500 mM imidazole), and storage buffer (25 mM potassium phosphate or HEPES pH 7.0, 150 mM NaCl, and 10% (v/v) glycerol). Purified protein was concentrated to 100−3000 μM and stored in pellets at −80 °C. Protein concentration was determined using Nanodrop based on a calculated extinction coefficient of 46870 $M^{-1}cm^{-1}$.

**Expression and purification of FlcD**

The pLIC-$His_6$-TEV-FlcD (PA3518) was cloned previously.[1] Expression and purification of $His_6$-tagged FlcD was performed using the same procedure and buffers as FlcE. Purified protein was concentrated to 100−3000 μM and stored in pellets at −80 °C. Protein concentration was determined using a Bradford assay or Nanodrop with a calculated extinction coefficient of 51340 $M^{-1}cm^{-1}$.

**Purification of anaerobic FlcD**

FlcD expression was performed mostly as described in “expression and purification of FlcD.” Nickel affinity chromatography was carried out in a vinyl anaerobic chamber (Coy Laboratory). All buffers were degassed and stored anaerobically for use. After cell lysis, cell lysate was brought into the anaerobic chamber without degassing. Buffer exchange was performed in the anaerobic chamber and a small aliquot of protein was taken out of the chamber for concentration determination. Purified protein was concentrated to 100−3000 μM and stored in pellets at −80 °C in O-ring sealed cryotubes to keep the protein anaerobic.

**Expression and purification of TEV protease**

The pLIC-TEV protease-$His_6$ was constructed previously.[2] BL21-CodonPlus (DE3)-RIPL cells were transformed with the plasmid and grown at 37 °C in 1 L of LB containing 100 μg/mL

ampicillin and 34 μg/mL chloramphenicol with shaking at 225 rpm. Cells were grown to an $OD_{600}$ of 0.2−0.8 and protein expression was induced with the addition of 0.5 mM IPTG. The temperature was then reduced to 16 °C and the cultures were grown overnight. $OD_{600}$ of cells was recorded, and cells were harvested by centrifugation at 4500 rcf for 30 min at 4 °C. The cell pellet was resuspended in ~20 mL wash buffer. Resuspended cells were kept on ice and sonicated at 30% amplitude for 5 min with a 1 s on and 1 s off cycle using a Sonic Dismembrator (Fisher Scientific model 500). Cellular debris was removed by centrifugation at 15000 rcf for 40 min at 4 °C. Cell lysate was clarified using a 0.45 μm filter. $His_6$-tagged TEV protease was then purified by nickel affinity chromatography on an AKTA Pure FPLC using a 5 mL HisTrap Column (GE). The wash buffer contained 50 mM sodium phosphate pH 8.0, 200 mM NaCl, 10% glycerol (v/v), and 30 mM imidazole. The elution buffer contained 50 mM sodium phosphate pH 8.0, 200 mM NaCl, and 10 % glycerol (v/v), and 500 mM imidazole. The storage buffer contained 50 mM sodium phosphate pH 8.0, 200 mM NaCl, and 10% (v/v) glycerol. Fractions of purified TEV protease were analyzed on a 12% SDS-PAGE gel (195 V for 45 min) and stained with Coomassie blue (1.25 g/L in 50% methanol, 10% glacial acetic acid). Semi-pure fractions were concentrated to 2.5 mL using an Amicon centrifugal filter with a 10 kDa cutoff (Millipore) and imidazole was removed from the protein using a PD-10 buffer exchange column. Semi-pure TEV protease was further purified by size exclusion chromatography on a Superdex 200 16/60 column equilibrated with storage buffer. Purified protein was concentrated to 100−200 μM, flash frozen, and stored as pellets at −80 °C. Protein concentration was determined using Nanodrop based on a calculated extinction coefficient of 33710 $M^{-1}cm^{-1}$.

**$His_6$-TEV-FIcD cleavage of $His_6$-tag with TEV protease**

Reactions were assembled using 175 μM of $His_6$-TEV-FIcD, 1.5 μM of TEV protease, and 20% v/v TEV cleavage buffer (250 mM Tris Base pH 8.0, 2.5 mM EDTA, and 5 mM dithiothreitol), and diluted to a volume of 7.5−49.2 mL using a modified FIcD storage buffer (25 mM HEPES pH 7.0 and 150 mM NaCl). Reaction volumes were set based on the amount of FIcD available for cleavage. Cleavage reactions were incubated at 4 °C for 16 h. FIcD with no $His_6$- tag ($FIcD_{tagless}$) was then purified by nickel affinity chromatography on an AKTA Pure FPLC using a 5 mL HisTrap column (GE) by collecting the flow-through after equilibrating the column with wash buffer. Same wash and elution buffers were used as described in "expression and purification of FIcD." Fractions containing pure $FIcD_{tagless}$ were concentrated to 2.5 mL using an Amicon centrifugal filter with a 30 KDa cutoff (Millipore) and imidazole was removed from the protein using a PD-10 buffer exchange column. Purified protein was concentrated to 0.1−5 mM and stored at −80 °C. Protein concentration was determined using Nanodrop based on a calculated extinction coefficient of 49850 $M^{-1}cm^{-1}$.

**Size exclusion chromatography multi-angle light scattering (SEC-MALS) analysis**

A 100 μL stock of 250 μM $FIcD_{tagless}$ (0.25 nmol) in 25 mM HEPES pH 7.0, 150 mM NaCl, and 10% glycerol was analyzed by SEC-MALS (Wyatt DAWN HELEOS II light scattering instrument interfaced to an Agilent FPLC equipped with a Superdex 200 Increase 10/300 GL column, Wyatt T-rEX refractometer, and Wyatt dynamic light scattering module). Separation was carried out at a flow rate of 0.5 mL/min for 50 min in a modified storage buffer (25 mM HEPES pH 7.0, 150 mM NaCl, and 0.1% w/v sodium azide). Peaks with UV-absorption were analyzed for the molecular weight and polydispersity index (PDI).

**Enzymatic synthesis and purification of FlcD substrate, 2**

FlcD substrate was prepared enzymatically using FlcB and FlcE. First, *S*-succinyl-cysteine (**1**) was generated in a reaction containing 25 μM FlcB, 50 mM L-cysteine, 50 mM sodium fumarate, and 200 mM bicine pH 9.0. After incubation for ~16 h at 25 °C, the reaction was quenched either by heating to 75 °C for 5 min and then placed on ice for 2 min or by the addition of an equal volume of acetonitrile. Typical reactions were 3−20 mL in volume. After the reaction was quenched, precipitated protein was removed via centrifugation at 21000 rcf for 5 min. Supernatant from the FlcB reaction was used without purification in a subsequent reaction with FlcE, which contained 20 μM FlcE, 250 μM $(NH_4)_2Fe(II)(SO_4)_2$, 1 mM sodium ascorbate, 25 mM potassium phosphate pH 7.0, 2 mM **1** (assuming 100% yield in the FlcB reaction), and 8 mM bicine. The reaction was separated into 200 μL aliquots in 96-well plates or petri dishes covered loosely with aluminum foil to allow aeration for ~16 h at 25 °C. Typical reactions were 10−200 mL in volume. To quench the FlcE reaction, 2 volumes of acetonitrile was added, and the reaction was placed on ice for ~10 min. Precipitated protein was pelleted by centrifugation at 4500 rcf for 20 min at 4 °C. Supernatant was collected and dried via rotary evaporation. Dried material was resuspended in 50% acetonitrile in water, filtered with a 0.2 μm filter, and purified via preparative HPLC with 0.1% TFA in the mobile phase. The yield of purification was increased by addition of 0.8% TFA to the reaction immediately before injection on HPLC. Compound **2** was separated from other reaction components on a Luna Prep C18 column (Phenomenex, 250 mm length, 10 μm particle size, and 100 Å pore size) at a flow rate of 12 mL/min using the following method. Solvent A contained 0.1% TFA in water, and solvent B contained 0.1% TFA in acetonitrile. Mobile phase was held at 2% B for 5 min, increased to 70% B over 15 min, then to 95% B over 10 min, and held at 95% B for 3 min before returning to 2% B over 5 min. Compound **2** eluted at 19−21 min. Purity of **2** was assessed by LC-MS and $^1$H NMR. MS data were collected using the "general method for liquid chromatography-mass spectrometry analysis." $^1$H NMR spectra were acquired in $D_2O$ on a Bruker Avance III at 850 MHz or a Bruker Avance NEO at 400 MHz. Fractions containing **2** were dried via rotary evaporation and resuspended in $H_2O$ to 50 or 100 mM and stored at −20 °C.

**Enzymatic synthesis and purification of FlcD product, 3**

FlcD product was prepared enzymatically using FlcB, FlcE, and FlcD. First, FlcD substrate, **2**, was generated as described in "enzymatic synthesis and purification of FlcD substrate, **2**." After the reaction to generate **2** was complete, supernatant was collected and dried via rotary evaporation. Dried material was resuspended in ¼ of the reaction volume of water and then mixed with 100 μM $(NH_4)_2Fe(II)(SO_4)_2$, 1 mM sodium ascorbate, and 25 μM FlcD and allowed to react for 16 h at 25 °C. The next day the reaction was quenched with the addition of 2 volumes of acetonitrile, centrifuged at 4500 rcf for 40 min at 4 °C, and the supernatant of the reaction was dried via rotary evaporation. Dried material was resuspended in 50% acetonitrile in water and purified via preparative HPLC with 0.1% TFA in the mobile phase. $H_2SO_4$ was added to the reaction mixture to 0.8% immediately before injection to improve the retention of **3** and separation from other assay components. Compound **3** was purified on the preparative HPLC using the same method as described in "enzymatic synthesis and purification of FlcD substrate, **2**." Compound **3** eluted at 17−19 min. Purity of product was assessed by LC-MS and $^1$H NMR. MS data were collected using the "general method for liquid chromatography-mass spectrometry analysis." $^1$H NMR spectra were acquired in $D_2O$ on a Bruker Avance III at 850 MHz. Pure fractions were dried via rotary evaporation and resuspended in water to 50 mM and stored at −20 °C.

**Standard curve of FlcD product, 3**

A standard curve of FlcD product was made to quantify the amount of product formation in various assays. FlcD product was diluted in water to concentrations of 2, 1.5, 1, 0.75, 0.5, 0.38, 0.25, 0.13, and 0.06 mM and analyzed using the "general method for liquid chromatography-mass spectrometry analysis." Each concentration was prepared in duplicate and measured using the UV absorbance at 250 nm. A linear regression was performed to generate this formula: ($UV_{250}$ peak area) = 0.8823 x (FlcD product concentration (μM)) + 10.001, which was used to quantify the amount of the FlcD product formed in an enzymatic assay.

**Activity of FlcD in a 30-min time course**

The activity of FlcD was assessed in a 30-min time course to determine the linear range for steady state kinetic analysis. FlcD stocks were diluted to 50 μM in storage buffer. A fresh solution of 10 mM $(NH_4)_2Fe(II)(SO_4)_2$ was prepared in milliQ water and diluted to 2.5 mM. A fresh solution of 50 mM sodium ascorbate was prepared in milliQ water. A master mix was assembled so that the reaction contained final concentrations of 25 mM potassium phosphate pH 7, 100 μM $(NH_4)_2Fe(II)(SO_4)_2$, 1 mM sodium ascorbate, and 1 mM of the FlcD substrate **2** in 350 μL. The reaction was initiated by addition of FlcD to 5 μM and incubated at 25 °C. Aliquots (25 μL) were removed from the master mix at 0, 1, 2, 3, 4, 10, 15, and 30 min and quenched in two volumes of acetonitrile. Quenched reactions were spun at 21000 rcf for 5 min to remove precipitated protein and the supernatant was collected to analyze on LC-MS using the "general method for liquid chromatography-mass spectrometry analysis." Formation of product was assessed by extracting the ion chromatogram for **3** ($[M+H]^+$ *m/z*: 194.0118). The time course was performed in duplicate.

**Crystallography**

Three FlcD crystals were grown anaerobically using the sitting drop vapor diffusion method at 20 °C inside an MBraun glovebox. A sample of 0.4 μL of FlcD (10 mg/mL) was mixed 1:1 with mother liquor containing either **A**) 0.2 M ammonium sulfate, 0.1 M Tris HCl pH 8.5, 25% (w/v) PEG 3350, (PDB: 9B9N) **B**) 0.2 M sodium chloride, 0.1 M sodium phosphate dibasic, citric acid pH 4.2, 20% (w/v) PEG 8000 (PDB 9B9O), or **C**) 0.1 M Bis-Tris HCl pH 6.5, 0.2 M $MgCl_2$, 25% (w/v) PEG 3350 (PDB: 9B9M). Co-crystal structures were obtained by soaking crystals anaerobically in their respective mother liquors that included 4 mM $(NH_4)_2Fe(II)(SO_4)_2$ and 1 mM substrate for 1−5 min prior to looping and flash cooling in liquid nitrogen. A FlcD crystal was grown aerobically in saturating 2.5 mM $(NH_4)_2Fe(II)(SO_4)_2$ using the sitting drop vapor diffusion method at 20 °C. A sample of 0.2 nL of FlcD (10.1 mg/mL) was mixed 2:1 with 0.1 nL mother liquor containing 0.2 M $MgCl_2$, 20% PEG 3350 (PDB: 8W1Q). This crystal was cryoprotected in 0.2 M $MgCl_2$, 20% PEG 3350, and 20% glycerol and flash cooled in liquid nitrogen. Diffraction data were collected for 180 degrees at 100 K at BNL NSLS-II 17-ID-1 for anaerobic crystals or collected for 195 degrees at APS GMCA 23-ID-D for the aerobic crystal. Anomalous maps were extracted from our native data collected at a wavelength of 0.9201 Å because iron absorbs strongly at this wavelength and magnesium does not. The reported refinement statistics are not generated from merging the anomalous data. Bijvoet pairs were kept separate to calculate the anomalous map. For the rest of the maps and structural refinement, the data were merged and the Bijvoet pairs were averaged. Data were processed and scaled with FastDP[3-6] or HKL2000.[7] Phaser within the Phenix suite (version 1.17.1-3660)[8] was used to perform molecular replacement using PDB 3BJD as a model. Output models from Phaser were refined iteratively using cycles of refinement in phenix.refine followed by manual adjustment using Coot (version

0.9.4.1).[9] $R_{free}$ reflection set was assigned by Phenix. Crystallography statistics are reported in Table S1. Structural figures were generated using Pymol (Schrödinger) version 2.2.3.

In the FlcD•Fe•substrate structure in the supplemental information (PDB 9B9O), our experimental data indicated two conformations for ligand binding in chain B. Therefore, we placed the ligand in two orientations in the active site, each with an initial occupancy of 0.5. The occupancy for each ligand was refined by phenix.refine to final values of 0.31 and 0.69, respectively.

**Cloning of FlcD mutants**

Site-directed mutagenesis was performed to generate single mutations in FlcD. PCRs of pLIC-His$_6$-TEV-FlcD were run using primers (Table S2) to generate linear product with the desired mutation. PCR product was extracted from an agarose gel using Zymo Gel Extraction Kit (Zymo Research). A kinase-ligase-DpnI (KLD) reaction was mixed in a microcentrifuge tube that contained 2 µL of PCR product of varying concentrations, 4 µL of autoclaved milliQ water, 1 µL T4 ligase buffer, 1 µL T4 ligase, 1 µL T4 polynucleotide kinase (PNK), and 1 µL DpnI. The reaction was incubated at room temperature for 1 h, followed by incubation at 37 °C for 30 min. NEB DH5α chemical competent cells were thawed on ice and 3 µL of the KLD reaction was added to 15−50 µL of cells. The mixture was incubated on ice for 5 min, heat shocked for 30 s at 42 °C, and grown in LB media at 37 °C with shaking for a maximum of 1 h. After transformation, cells were plated on LB plates containing 50 µg/mL ampicillin. Individual colonies were selected for overnight cultures and plasmid isolation. Purified plasmid was sequenced using the T7 promoter and T7 terminator universal primers at EtonBio (Research Triangle Park, NC). BL21 (DE3) cells were transformed with sequence verified plasmids and FlcD variant was expressed and purified using the procedure described in “expression and purification of FlcD”.

**Activity measurement of FlcD variants**

Aliquots of FlcD WT and variant proteins were thawed on ice. The concentration of each sample was measured on a Nanodrop and diluted to 50 µM in storage buffer. A fresh 10 mM solution of $(NH_4)_2Fe(II)(SO_4)_2$ was prepared in milliQ water and diluted to 2.5 mM in milliQ water. Fresh sodium ascorbate was prepared at 50 mM in milliQ water. Reaction components were assembled in a master mix containing 25 mM potassium phosphate pH 7.0, 100 µM $(NH_4)_2Fe(II)(SO_4)_2$, 1 mM sodium ascorbate, and 1 mM FlcD substrate **2**. The master mix was separated into 25 µL aliquots and the reaction was initiated with the addition of FlcD (WT or variant) to 5 µM. The reaction was incubated for 2 min or 2 h at 25 °C and quenched with the addition of two volumes of acetonitrile. Quenched reactions were spun at 21000 rcf for 5 min to pellet protein and the supernatant was collected and analyzed using the “general method for liquid chromatography-mass spectrometry analysis.” Formation of product was assessed by extracting the ion chromatogram for **3** ($[M+H]^+$ *m/z*: 194.0118). Each replicate contained a WT control. Percent relative activity was obtained by taking the ratio of the integrated peak areas of each mutant to WT and multiplying by 100. Variants that retained activity >5% at 2 h were also tested at 2 min to measure the initial velocity.

**EPR and Mössbauer spectroscopy**

Mössbauer spectra were recorded on a spectrometer from WEB Research, equipped with a Janis SVT-400 variable-temperature cryostat. The external magnetic field (78 mT) was applied parallel to the γ beam when spectra were acquired at 4.2 K. No magnetic field was applied for samples recorded at 80 K. All isomer shifts are quoted relative to the centroid of the

spectrum of α-iron metal at room temperature. Mössbauer spectra were simulated using the WMOSS spectral analysis software (www.wmoss.org, WEB Research, Edina, MN). EPR spectra were acquired on a Bruker E500 Elexsys continuous wave (CW) X-Band spectrometer (operating at approximately 9.36 GHz) equipped with a rectangular resonator (TE102) and a continuous-flow cryostat (Oxford 910) with a temperature controller (Oxford ITC 503). The spectra were recorded at variable temperatures between 10 K and 20 K at a microwave power of 2 mW, using a modulation amplitude of 1 mT, and a microwave frequency of 9.36 GHz. Baseline subtraction and spectral analysis were performed with Kazan (https://sites.psu.edu/silakovlab/software/), a MATLAB GUI created by Drs. Alexey Silakov and Boris Epel.

**Preparation of rapid freeze-quench (RFQ) EPR and Mössbauer samples**

RFQ experiments were carried out with an Update Instruments 1000 apparatus that was kindly gifted by JoAnne Stubbe. FlcD (1.7 mM) was reconstituted under anoxic conditions (Coy glovebox) with 1 molar eq of $^{57}$Fe(II) and 3 molar eq of substrate and was subsequently mixed against $O_2$-saturated buffer (1.8 mM $O_2$) in a 1:1 ratio at 5 °C. The samples were allowed to age for the appropriate time prior to rapid freezing in an isopentane bath at −155 °C. The anaerobic control sample was extracted under anoxic conditions from the syringe for both EPR and Mössbauer samples.

**Preparation of acid quenched samples**

Rapid mixing experiments were performed with an Update Instruments 1000 apparatus, but instead of the samples being rapidly frozen, they were rapidly quenched in a solution of 3.5% sulfuric acid ($H_2SO_4$) in a 1:1 ratio. Product formation at the respective acid-quench time-points was quantified as follows: the quenched samples were diluted 1:2 in acetonitrile and analyzed using the "general method for liquid chromatography-mass spectrometry analysis." The absorbance at 250 nm was used to quantify the amount of product using the linear fit produced in "standard curve of FlcD product, **3**."

**Stopped-flow absorption spectroscopy (SF-Abs)**

SF-Abs measurements were carried out with a SX20 stopped-flow spectrophotometer from Applied Photophysics Ltd (Leatherhead, UK) that was housed in an anoxic chamber (Coy). All experiments were carried out at 5 °C. In single-mix (two-syringe) experiments, the FlcD reactant solution containing 1.7 mM FlcD, 1 molar eq of Fe(II) and 3 molar eq of substrate was diluted two-fold after mixing with a solution of an $O_2$-saturated buffer at 5 °C (1.8 mM). The optical path length used was 10 mm. For the characterization of the reactions, the photodiode array detector (PDA) was used to acquire time-resolved absorption spectra.

**Anaerobic preparation of $FlcD_{tagless}$ for determining iron equivalence**

All preparations were performed in a Coy vinyl anaerobic chamber. A 10 mM $(NH_4)_2Fe(II)(SO_4)_2$ solution was freshly made using degassed storage buffer (25 mM HEPES pH 7.0, 150 mM NaCl). An aliquot of purified **2** was dried and resuspended to 100 mM in degassed storage buffer. $FlcD_{tagless}$ was thawed in the chamber, diluted to ~250 µM (2.5 mL), loaded onto a PD-10 equilibrated with degassed storage buffer, and eluted with 3.5 mL of buffer to remove $O_2$. $FlcD_{tagless}$ was further diluted to 112.5 µM. In a microcentrifuge tube, 1 mL of 112.5 µM $FlcD_{tagless}$ was reconstituted using 150 µL of 10 mM $(NH_4)_2Fe(II)(SO_4)_2$ (13.3 equivalents) and then mixed with either 350 µL of storage buffer or 335 µL of storage buffer and 15 µL of 100 mM substrate, **2**. Reconstituted $FlcD_{tagless}$ samples were then incubated on ice for 45 min. As a control, $FlcD_{tagless}$ was incubated on ice without any treatment to assess the iron content of

FlcD$_{tagless}$ as purified. To remove excess iron, the reconstituted FlcD$_{tagless}$ samples (1.5 mL each) were diluted in 15 mL of storage buffer and concentrated to 250 μL using a Teflon tape-sealed Amicon centrifugal filter (30 KDa cutoff). The wash step was repeated once. The resulting sample was mixed with 750 μL of storage buffer to a final volume of 1 mL. Iron content of each sample was quantified below in "ferrozine assay for iron equivalence testing of FlcD." Reconstitution of FlcD$_{tagless}$ in the presence of iron or iron and substrate was performed in triplicate.

**Ferrozine assay for iron equivalence testing of FlcD**

Solutions include freshly made 10 mM $(NH_4)_2Fe(II)(SO_4)_2$, freshly made 75 mM sodium ascorbate, saturating ammonium acetate, and 10 mM ferrozine (3-(2-pyridyl)-5,6-diphenyl-1,2,4-triazine-p,p′-disulfonic acid monosodium salt hydrate). A 10 mM $(NH_4)_2Fe(II)(SO_4)_2$ stock was diluted to 300 μM in water and used to make 0−300 μM standards via serial dilution. A 70.8 μL aliquot of each standard solution was added into a 1.5 mL microcentrifuge tube containing 14.2 μL of 10% trichloroacetic acid (TCA) to a final concentration of 1.67% TCA in 85 μL. Absorbances of anaerobically reconstituted FlcD$_{tagless}$ samples at 280 nm were determined using Nanodrop and their concentrations were calculated based on a calculated extinction coefficient of 49850 $M^{-1}cm^{-1}$. Protein concentrations were kept in the range of 50−150 μM for quantification of iron content. An 83.3 μL sample of protein was mixed with 16.7 μL of 10% TCA to a final concentration of 1.67% TCA. The resulting sample was spun for 2 min at 21000 x g to remove precipitated proteins. A sample of 85 μL supernatant or iron standard was added to a microcentrifuge tube containing 20 μL of 75 mM sodium ascorbate, 20 μL of 10 mM ferrozine, 120 μL of saturating ammonium acetate, and 415 μL of milliQ water (final volume 660 μL). A 250 μL aliquot of each sample was added into a 96-well flat clear bottom plate and the absorbance at 562 nm was measured. A standard curve was produced using the iron standards and the concentration of iron was calculated for the FlcD samples. The ratio of iron to protein was plotted in a bar graph showing the mean and SEM for triplicate samples.

**Measuring FlcD activity in the presence of varying iron equivalents**

A stock of FlcD and substrate **2** were thawed, and a fresh stock of 10 mM $(NH_4)_2Fe(II)(SO_4)_2$ and 50 mM sodium ascorbate were prepared in milliQ water. A 33 μL sample was assembled to contain a final concentration of 10 μM FlcD, 1 mM sodium ascorbate, 2 mM substrate **2** in storage buffer (25 mM HEPES pH 7.0, 150 mM NaCl), and 50 mM HEPES pH 7.5. $(NH_4)_2Fe(II)(SO_4)_2$ was added at 0, 0.5, 1, 2, or 10 equivalents of the FlcD concentration. The reaction was initiated with the addition of FlcD. At 2 min, the 33 μL reaction was quenched with the addition of 33 μL of 3.75% TFA in milliQ water. Quenched reactions were centrifuged at 21000 rcf for 5 min to remove protein precipitates. The supernatant was collected and 11.7 μL of 5.25% TFA in acetonitrile was added to a final volume of 77.7 μL. The reaction was analyzed using the "general method for liquid chromatography-mass spectrometry analysis" with a modified gradient: mobile phase held at 2% B for 2 min, increased to 67% B over 7 min, further increased to 98% B over 3 min, and held at 98% B for 2 min before returning to 2% B over 1 min. The amount of product formed at each timepoint was quantified by integrating the absorbance peak at 250 nm and calculated using the FlcD product standard curve. The extracted ion chromatogram ($[M+H]^+$ *m/z*: 194.0118) was used to verify the retention time of product. Samples for each iron equivalent were prepared and measured in triplicate and the triplicate values were plotted to obtain the average and standard error of the mean (SEM).

**Isotope tracing of [$^{13}C_3$, $^{15}N$]-Cys in the FlcE and FlcD reactions**

The FlcE substrate, [$^{13}C_3$, $^{15}N$]-**1**, was produced in a reaction containing 25 µM FlcB, 50 mM [1,2,3-$^{13}C_3$, $^{15}N$] L-cysteine, 50 mM sodium fumarate, and 200 mM bicine pH 9.0 in a total volume of 511 µL. The reaction was incubated for ~ 16 h at 25 °C and quenched by heating to 75 °C for 5 min and then placed on ice for 2 min. Precipitated protein was removed by centrifugation at 21000 rcf for 5 min. The supernatant of the FlcB reaction was directly used in the FlcE and FlcD one-pot reaction without purification. This supernatant was mixed with components of the FlcE and FlcD reactions to final concentrations of 10 µM FlcE, 10 µM FlcD, 200 µM $(NH_4)_2Fe(II)(SO_4)_2$, 1 mM sodium ascorbate, 25 mM potassium phosphate pH 7.0, 2 mM [$^{13}C_3$, $^{15}N$]-**1** (assuming 100% yield from the FlcB reaction), and 8 mM bicine pH 9.0 in 50 µL. The reaction was incubated for 6 h at 25 °C and quenched with the addition of two volumes of acetonitrile. Precipitated protein was pelleted via centrifugation at 21000 rcf for 5 min and the formation of product was analyzed using the "general method for liquid chromatography-mass spectrometry analysis." Production of [$^{13}C$,$^{15}N$]-**3** was assessed using the extracted ion chromatogram of its expected $[M+H]^+$ *m/z*: 196.0122.

**Isotope tracing of [2,3,3-$D_3$]-Cys in the FlcE and FlcD reactions**

The FlcE substrate, [$D_3$]-**1**, was prepared in a reaction containing 25 µM FlcB, 50 mM [2,3,3-$D_3$] L-cysteine, 50 mM sodium fumarate, and 200 mM bicine pH 9.0 in a total volume of 467 µL. The reaction was incubated for ~ 16 h at 25 °C and quenched by heating to 75 °C for 5 min and then placed on ice for 2 min. Precipitated protein was removed by centrifugation at 21000 rcf for 5 min. The supernatant of the FlcB reaction was directly used in the FlcE and FlcD one-pot reaction without purification. This supernatant was mixed with components of the FlcE and FlcD reactions to final concentrations of 10 µM FlcE, 10 µM FlcD, 200 µM $(NH_4)_2Fe(II)(SO_4)_2$, 1 mM sodium ascorbate, 25 mM potassium phosphate pH 7.0, 2 mM [2,3,3-$D_3$]-**1** (assuming 100% yield from the FlcB reaction), and 8 mM bicine pH 9.0 in 50 µL total volume. The reaction was incubated for 6 h at 25 °C and quenched with the addition of two volumes of acetonitrile. Precipitated protein was pelleted via centrifugation at 21000 rcf for 5 min and the formation of product was analyzed using the "general method for liquid chromatography-mass spectrometry analysis." Production of **3** and [D]-**3** was assessed using the extracted ion chromatogram of each molecule (**3**, $[M+H]^+$ *m/z*: 194.0118; [D]-**3**, *m/z*: 195.0180).

**Isotope tracing of oxygen in the one-pot FlcE and FlcD reactions**

Reactions were performed in an anaerobic chamber. FlcD substrate was prepared enzymatically using FlcE and **1** as described in "enzymatic synthesis and purification of FlcD substrate, **2**." FlcE and FlcD purified anaerobically were thawed. Solutions of 10 mM $(NH_4)_2Fe(II)(SO_4)_2$ and 50 mM sodium ascorbate were prepared using degassed milliQ water in the chamber. An aliquot of purified **1** was dried and resuspended anaerobically to the initial volume in milliQ water at 100 mM. A one-pot reaction contained 10 µM FlcE, 10 µM FlcD, 200 µM $(NH_4)_2Fe(II)(SO_4)_2$, 1 mM sodium ascorbate, 25 mM potassium phosphate pH 7.0, and 2 mM **1**. The $^{16}O_2$ reaction was initiated by opening the reaction to atmosphere outside of the anaerobic chamber. The $^{18}O_2$ reaction was initiated with the addition of $^{18}O_2$ inside the chamber. The anaerobic control reaction was initiated with the addition of FlcD inside the chamber. The $^{16}O_2$ and $^{18}O_2$ reactions were mixed shaking by hand and incubated for 3 h at 25 °C. Reactions were quenched with the addition of two volumes of acetonitrile (degassed acetonitrile for anaerobic and $^{18}O_2$ samples). Precipitated protein was pelleted via centrifugation at 21000 rcf for 5 min and the formation of product was assessed using the "general method for liquid

chromatography-mass spectrometry analysis.” Production of **2**, $^{18}O$-**2**, **3** and $^{18}O$-**3**) was assessed using the extracted ion chromatogram of the molecule (**2**, $[M+Na]^+$ *m/z*: 230.0099; $^{18}O$-**2**, $[M+Na]^+$ *m/z*: 232.0142; **3**, $[M+H]^+$ *m/z*: 194.0118; $^{18}O$-**3**, $[M+H]^+$ *m/z*: 196.0160).

**Isotope tracing of oxygen in the FlcD reaction**

FlcD substrate, **2**, was prepared enzymatically using FlcE and **1** as described in “enzymatic synthesis and purification of FlcD substrate, **2**.” FlcE and FlcD were purified anaerobically. Solutions of 10 mM $(NH_4)_2Fe(II)(SO_4)_2$ and 50 mM sodium ascorbate were prepared using degassed milliQ water in the chamber. An aliquot of purified **1** was dried and resuspended anaerobically to the initial volume in milliQ water at 100 mM. The FlcE reaction contained 10 µM FlcE, 100 µM $(NH_4)_2Fe(II)(SO_4)_2$, 1 mM sodium ascorbate, 25 mM potassium phosphate pH 7.0, and 2 mM **1** in 150 µL total volume. The reaction was initiated with the addition of $^{16}O_2$ or $^{18}O_2$ and incubated for 3 h at 25 °C. The reaction was quenched with the addition of two volumes of degassed acetonitrile. Precipitated protein was pelleted via centrifugation at 21000 rcf for 5 min and the supernatant was dried via a centrifugal vacuum concentrator. The $^{18}O_2$ reaction was resuspended in the anaerobic chamber with degassed milliQ water to the original volume of 147 µL (less 3 µL for LC-MS analysis). The $^{16}O_2$ reaction was resuspended in air in milliQ water to the same volume. $(NH_4)_2Fe(II)(SO_4)_2$ was added to a final concentration of 200 µM, sodium ascorbate to 2 mM, and FlcD to 10 µM. The reaction was initiated with the addition of $^{18}O_2$ for the $^{16}O$-**2** substrate or by the addition of FlcD for the $^{16}O_2$ reaction with the $^{18}O$-**2** substrate. FlcD reactions were incubated for 3 h at 25 °C. The reaction was then quenched with the addition of two volumes of acetonitrile (degassed for $^{18}O_2$ samples). Precipitated protein was pelleted via centrifugation at 21000 rcf for 5 min and the formation of product was assessed using the “general method for liquid chromatography-mass spectrometry analysis.” Production of **2** and $^{18}O$-**2** in the FlcE reaction was assessed using the extracted ion chromatogram of the molecule (**2**, $[M+Na]^+$ *m/z*: 230.0099;$^{18}O$-**2**, $[M+Na]^+$ *m/z*: 232.0142). Production of **3** and $^{18}O$-**3** in the FlcD reaction was assessed using the extracted ion chromatogram of the molecule (**3**, $[M+H]^+$ *m/z*: 194.0118; $^{18}O$-**3**, $[M+H]^+$ *m/z*: 196.0160).

**Isotope tracing of oxygen in formic acid using [$^{13}C_2$, $^{15}N$]-2 in the FlcD reaction**

FlcD substrate containing [$^{13}C$, $^{15}N$] label was prepared enzymatically with FlcB and FlcE. The compound [$^{13}C_3$, $^{15}N$]-**1** was produced in a reaction containing 25 µM FlcB, 50 mM [1,2,3-$^{13}C_3$, $^{15}N$]-L-cysteine, 50 mM sodium fumarate, and 200 mM bicine pH 9.0 in 799 µL total volume. The reaction was incubated for ~16 h at 25 °C and quenched by heating to 75 °C for 5 min and then placed on ice for 2 min. Precipitated protein was pelleted at 21000 rcf for 5 min and the supernatant was used without purification in the FlcE reaction. The FlcE reaction contained 125 µM FlcE, 400 µM $(NH_4)_2Fe(II)(SO_4)_2$, 1 mM sodium ascorbate, 25 mM potassium phosphate pH 7.0, 4 mM [$^{13}C_3$, $^{15}N$]-**1** (assuming 100% yield from the FlcB reaction), and 16 mM bicine pH 9.0 in a total volume of 500 µL. The reaction was incubated overnight with the cap open for ~16 h at 25 °C. The reaction was then quenched by the addition of two volumes of acetonitrile. Precipitated protein was pelleted via centrifugation at 21000 rcf for 5 min. Supernatant was collected and dried via rotary evaporation and stored at −80 °C if not used immediately. The dried FlcE reaction was resuspended in an equal volume of anaerobic milliQ water in the FlcD reaction. FlcD was purified anaerobically to a final concentration of 2−3 mM. Stocks of 100 mM $(NH_4)_2Fe(II)(SO_4)_2$ and 200 mM sodium ascorbate were prepared in anaerobic milliQ water. The FlcE reaction was resuspended to the initial volume of 500 µL, and 320 µM FlcD, 1 mM sodium ascorbate, 1 mM $(NH_4)_2Fe(II)(SO_4)_2$, and anaerobic milliQ water were added to a final volume of 625 µL. The reaction was started either by the addition of $^{18}O_2$,

bringing the vial out of the anaerobic chamber ($^{16}O_2$ reaction), or by addition of FlcD (anaerobic control). The reaction was incubated for 4 or 10 min, then simultaneously quenched and extracted by the addition of an equal volume of diethyl ether containing 10% $H_2SO_4$ to a final concentration of 1.67% $H_2SO_4$ in the aqueous layer. The extractions were shaken vigorously for ~10 s, the organic layer was collected, and the extraction was performed three more times. Organic layers from each extraction were pooled and concentrated by evaporating excess diethyl ether in air to a final volume that is approximately 25% of the original reaction. A formic acid control was treated the same as the FlcD reactions, with the addition of storage buffer in place of FlcD and the addition of formic acid at 3.2 mM. Samples were analyzed using the "general method for gas chromatography-mass spectrometry analysis." Production of formic acid was assessed using the extracted ion chromatogram of the expected ions: $^{13}CHO_2$ $[M]^+$ *m/z*: 46.00046, $^{13}CO^{18}O$ $[M]^+$ *m/z*: 46.99687, $^{13}CH_2O_2$ $[M]^+$ *m/z*: 47.00829, $^{13}CHO^{18}O$ $[M]^+$ *m/z*: 48.00471, $^{13}C^{18}O_2$ $[M]^+$ *m/z*: 49.00113, $^{13}CH_2O^{18}O$ $[M]^+$ *m/z*: 49.01253, and $^{13}CH^{18}O_2$ $[M]^+$ *m/z*: 50.00895.

**Tracing deuterium in the FlcD reaction using deuterated 2**

Production of [$^{13}C_3$,$^{15}N$, $D_3$]-**1** and [$D_3$]-**1** was performed in a reaction containing 25 µM FlcB, 50 mM [$^{13}C_3$,$^{15}N$, $D_3$]- or [$D_3$]-L-cysteine, 50 mM sodium fumarate, and 200 mM bicine pH 9.0 in 805 µL. The reaction was incubated for ~16 h at 25 °C and quenched by heating to 75 °C for 5 min and then placed on ice for 2 min. Precipitated protein was pelleted at 21000 rcf for 5 min. Supernatant from the FlcB reaction was used without purification in FlcE and FlcD reactions. A reaction was assembled containing 125 µM FlcE, 400 µM $(NH_4)_2Fe(II)(SO_4)_2$, 1 mM sodium ascorbate, 25 mM potassium phosphate pH 7.0, 4 mM [$^{13}C_3$,$^{15}N$, $D_3$]-**1** or [$D_3$]-**1** (assuming 100% yield from the FlcB reaction), and 16 mM bicine pH 9.0 in a total volume of 500 µL. The reaction was incubated overnight with the cap open for ~16 h at 25 °C. The reaction was then quenched by the addition of two volumes of acetonitrile. Precipitated protein was pelleted via centrifugation at 21000 rcf for 5 min. Supernatant was collected and dried via rotary evaporation and stored at −80 °C if not used immediately. FlcD was purified to a final concentration of ~2−3 mM. Stocks of 100 mM $(NH_4)_2Fe(II)(SO_4)_2$ and 200 mM sodium ascorbate were prepared in anaerobic milliQ water. The dried FlcE reaction was resuspended to the initial volume of 500 µL and mixed with 320 µM FlcD, 1 mM sodium ascorbate, 1 mM $(NH_4)_2Fe(II)(SO_4)_2$, and milliQ water to a final volume of 625 µL. The reaction was started by addition of FlcD and incubated for 10 min, then simultaneously quenched and extracted by the addition of an equal volume of diethyl ether containing 10% $H_2SO_4$ to a final concentration of 1.67% $H_2SO_4$ in the aqueous layer. The extractions were mixed via shaking vigorously for ~10 s, the organic layer was collected, and the extraction was repeated three more times. Organic layers from each extraction were pooled and concentrated by blowing off excess diethyl ether with air to a final volume that is approximately 25% of the original reaction.

A [D]-formic acid control was treated the same as the FlcD reactions, with the addition of storage buffer in place of FlcD and the addition of [D]-formic acid at 3.2 mM. Samples were analyzed using the "general method for gas chromatography-mass spectrometry analysis." Production of formic acid was assessed using the extracted ion chromatogram of the expected ions: $CO_2$ $[M]^+$ *m/z*: 43.98928, $^{13}CO_2$ $[M]^+$ *m/z*: 44.99264, $CHO_2$ $[M]^+$ *m/z*: 44.99711, $^{13}CHO_2$ $[M]^+$ *m/z*: 46.00047, $CDO_2$ $[M]^+$ *m/z*: 46.00341, $^{13}CDO_2$ $[M]^+$ *m/z*: 47.00675, $CH_2O_2$ $[M]^+$ *m/z*: 46.00493, $^{13}CH_2O_2$ $[M]^+$ *m/z*: 47.00824, $CHDO_2$ $[M]^+$ *m/z*: 47.01125, and $^{13}CHDO_2$ $[M]^+$ *m/z*: 48.01456.

**NMR analysis of FlcD reaction to calculate turnovers**

A stock of FlcD and its substrate, **2**, were thawed, and a fresh stock of 10 mM $(NH_4)_2Fe(II)(SO_4)_2$ was prepared in $D_2O$. A reaction was assembled containing 10 µM FlcD, 1 mM substrate **2**, 8 µM $(NH_4)_2Fe(II)(SO_4)_2$ (0.8 equivalents of FlcD), and 25 mM potassium phosphate pH 7.0 in $D_2O$, adjusting the final volume of the reaction to 500 µL with $D_2O$. Reactions contained ~2% (v/v) of $H_2O$. The reaction was initiated with the addition of FlcD and incubated for 3 h. At the end of the reaction, benzoic acid in $D_6$-DMSO was added at a final concentration of 495 µM. The reaction was transferred to an NMR tube and analyzed using $^1H$ NMR. The amount of the FlcD product, **3**, formed in the reaction was determined using integrated peaks of **3** at ~ 7.67 ppm and benzoic acid at ~ 7.79 ppm. $^1H$ NMR spectra were acquired on a Bruker Avance III at 850 MHz.

**Sequence alignment of FlcD and FlcE to other characterized HDOs**

Sequences of FlcD (AAG06906.1), FlcE (AAG06907.1), SznF (QBA82042.1), UndA (WP_011062591.1), BesC (WP_014151496.1), and CADD (O84616.1) were aligned using Clustal Omega multiple sequence alignment tools from EMBL-EBI[10] and visualized using Jalview Version 2.[11]

**Bioinformatic prediction of iron-binding motifs in HDOs (PFAM14518)**

The previous sequence similarity network (SSN)[1] was used to analyze the potential iron-binding residues of the HDO family of enzymes, PFAM14518 (IPR016084). The entries started with 5325 unique sequences dispersed amongst 229 groups. Using the rentrez toolbox (version 1.2.3)[12] in RStudio,[13] the FASTA sequence of a randomly selected member from each node of the SSN was downloaded using the NCBI or EMBL ID. To accurately analyze the sequences, the beginning and end residue of the HDO domain of each sequence was downloaded from Interpro[14] using a Python script. The information from Interpro was matched to the corresponding FASTA sequence using MATLAB version 2022a. Sequences that were missing an NCBI or EMBL ID, missing the HDO domain start and end site information, or sequence entries that had been removed on NCBI or EMBL were not further analyzed.

The FASTA sequences were trimmed to contain only the HDO domain using a custom algorithm, resulting in 5040 unique sequences. The canonical iron-binding residues (Asn, Asp, Glu, and His) or those found in the conserved motif of FlcD (Val and Arg) were each assigned a unique number to allow searching for motifs via an algorithm, with all other residues set to be "0". These data were imported into MATLAB and a custom algorithm was written to search for the iron-binding motif within each HDO domain. Briefly, the spacing of iron-binding residues was analyzed amongst the six characterized HDO members to define the search criteria on the first 50 groups within the SSN. Between the first and second canonical iron-binding residues, ten residues are found in FlcD, FlcE, and SznF, seven residues in CADD and BesC, and three residues in UndA. FlcD, FlcE, SznF, CADD, BesC, and UndA all have four residues between the fourth and fifth iron-binding residues, and three residues between the fifth and sixth iron-binding residues (Figure S53). Initial screening of these motifs revealed alternative spacing of residues in uncharacterized groups that was not observed in characterized HDOs. To predict the iron-binding motif of each group in the SSN, each group was aligned to FlcD, FlcE, SznF, UndA, and CADD via multiple sequence alignment. Not every group contained the expected spacing of the iron-binding motif, thus random members from each of the first 50 groups were analyzed using AlphaFold2 model structures to further support the predicted spacing of identified residues by comparing the 3-D position of each identified iron-binding residue. From this comparison,

different patterns in the spacing of iron-binding motifs (Fig. S53) were identified and used in the search of the top 150 groups, representing 4898 unique sequences, or ~97% of all previously filtered (5040) sequences. Another filter was applied to constrain the location of the first two residues and the last three residues in each motif, because the first two iron-binding residues consistently occur in the first half of the HDO domain, while the last three residues consistently occur in the second half. Thus, if the first two residues were found in the second half of the sequence, the predicted motif was discarded. Histograms were used to visualize the patterns of each motif with boxes denoting the filter regions (Fig. S54–57). A heat map was produced to show how well each group matched the predicted motif for the first two and last three residues separately (Fig. S58–59). The standalone residue on the α2-helix was not analyzed.

**<u>Supplemental Tables:</u>**

**Table S1. X-ray crystallography data collection and refinement statistics.**

| | FlcD•Fe (Main Text) | FlcD•Fe•substrate (Main text) | FlcD•Fe•substrate (SI) | FlcD•Fe (Excess Fe -SI) |
|---|---|---|---|---|
| PDB code | 9B9M | 9B9N | 9B9O | 8W1Q |
| Wavelength | 0.9201 | 0.9201 | 0.9201 | 1.033 |
| Resolution range | 29.35 - 2.07 (2.144 - 2.07) | 28.63 - 2.284 (2.365 - 2.284) | 28.55 - 2.16 (2.238 - 2.16) | 31.57 - 1.56 (1.616 - 1.56) |
| Space group | P 1 21 1 | C 1 2 1 | C 1 2 1 | P 1 21 1 |
| Unit cell | 71.941 114.061 103.952 90 102.537 90 | 124.953 55.865 96.462 90 96.882 90 | 124.991 55.849 96.558 90 96.797 90 | 52.461 83.551 68.829 90 97.702 90 |
| Total reflections | 194359 (19541) | 58866 (5631) | 68606 (6394) | 241006 (18154) |
| Unique reflections | 99235 (9909) | 30091 (2896) | 35471 (3373) | 79607 (7453) |
| Multiplicity | 2.0 (2.0) | 2.0 (1.9) | 1.9 (1.9) | 3.0 (2.4) |
| Completeness (%) | 99.19 (99.97) | 99.30 (96.24) | 99.08 (94.83) | 95.19 (89.23) |
| Mean I/sigma(I) | 9.70 (1.64) | 9.50 (1.53) | 9.85 (1.34) | 6.46 (1.32) |
| Wilson B-factor | 33.32 | 46.01 | 43.22 | 18.18 |
| R-merge | 0.05238 (0.4512) | 0.04726 (0.4915) | 0.04369 (0.5479) | 0.09696 (0.4948) |
| R-meas | 0.07408 (0.638) | 0.06684 (0.695) | 0.06178 (0.7749) | 0.1166 (0.6235) |
| R-pim | 0.05238 (0.4512) | 0.04726 (0.4915) | 0.04369 (0.5479) | 0.0637 (0.373) |
| CC1/2 | 0.997 (0.676) | 0.997 (0.591) | 0.997 (0.603) | 0.992 (0.508) |
| CC* | 0.999 (0.898) | 0.999 (0.862) | 0.999 (0.868) | 0.998 (0.821) |
| Reflections used in refinement | 98829 (9910) | 30083 (2896) | 35460 (3373) | 79607 (7453) |
| Reflections used for R-free | 1989 (200) | 2000 (192) | 2000 (190) | 1995 (186) |
| R-work | 0.1992 (0.2616) | 0.2032 (0.3094) | 0.1940 (0.2964) | 0.1758 (0.2674) |
| R-free | 0.2416 (0.2805) | 0.2501 (0.3648) | 0.2329 (0.3182) | 0.2083 (0.3233) |
| CC(work) | 0.945 (0.829) | 0.965 (0.712) | 0.967 (0.745) | 0.966 (0.768) |
| CC(free) | 0.900 (0.781) | 0.954 (0.584) | 0.965 (0.763) | 0.955 (0.587) |
| Number of non-hydrogen atoms | 11281 | 5363 | 5511 | 5694 |
| Macromolecules | 10420 | 5207 | 5274 | 5137 |
| Ligands | 8 | 28 | 41 | 11 |
| Solvent | 853 | 128 | 196 | 546 |
| Protein residues | 1311 | 652 | 657 | 652 |

| | | | | |
|---|---|---|---|---|
| RMS(bonds) | 0.009 | 0.012 | 0.01 | 0.006 |
| RMS(angles) | 1.26 | 1.59 | 1.42 | 0.83 |
| Ramachandran favored (%) | 98.1 | 98.5 | 98.9 | 98.9 |
| Ramachandran allowed (%) | 1.7 | 1.4 | 1.1 | 1.1 |
| Ramachandran outliers (%) | 0.2 | 0.2 | 0.0 | 0.0 |
| Rotamer outliers (%) | 0.7 | 1.5 | 0.8 | 1.0 |
| Clashscore | 5.3 | 7.4 | 7.1 | 3.7 |
| Average B-factor | 46.1 | 67.6 | 63.1 | 25.4 |
| Macromolecules | 46.2 | 68.0 | 63.6 | 24.8 |
| Ligands | 63.8 | 81.8 | 74.7 | 34.5 |
| Solvent | 43.7 | 46.7 | 47.3 | 31.7 |
| Number of TLS groups | 21 | 14 | 12 | 0 |

**Table S2. Individual B-factors of iron and substrate ligands.**

| Structure | Monomer Label | Monomer | Iron | Substrate |
|---|---|---|---|---|
| FlcD•Fe PDB: 9B9M | C | 45.59 | 39.20 | |
| | D | 58.15 | 45.87 | |
| FlcD•Fe PDB: 8W1Q | A | 23.13 | 18.63 | |
| | B | 26.38 | 22.25 | |
| FlcD•Fe•2 PDB: 9B9N | A | 69.45 | 50.42 | 88.46 |
| | B | 66.75 | 56.82 | 79.52 |
| FlcD•Fe•2 PDB: 9B9O | A | 61.73 | 53.62 | 86.64 |
| | B | 65.52 | 55.75 | 70.22 |

**Table S3. Primers used in this study.**

| Primer name | Gene | Mutation | Direction | Primer sequence |
|---|---|---|---|---|
| WCS152 | FlcD | R121A | F | GCGCAGTCC**GCC**GAGCGGCACCCG |
| WCS153 | FlcD | R121A | R | GGCGATTTCCTGTTCGAGACG |
| WCS158 | FlcD | Q147A | F | CCTCCGTCAC**GCG**TGGTTCCGCAC |
| WCS159 | FlcD | Q147A | R | AACACCTGGAGCTGCGCGCGG |
| WCS173 | FlcD | R150A | F | ACCAGTGGTTC**GCG**ACCTTCCGCC |
| WCS174 | FlcD | R150A | R | GACGGAGGAACACCTGGAGCT |
| AP9 | FlcD | Y177F | F | CGCTCGCGCGT**TTC**CTCTATGGCGAG |
| AP10 | FlcD | Y177F | R | CCGCGGCCTCGTCCACATCGGTC |
| AP1 | FlcD | E181A | F | CTCTATGGC**GCG**TTGGGCGAGGAAG |
| AP2 | FlcD | E181A | R | GTAACGCGCGAGCGCCGCGGCCTCGTC |
| AP3 | FlcD | H191A | F | GAAAAGGGTTCG**GCG**CCGCGCCTGCTG |
| AP4 | FlcD | H191A | R | GTCTTCCTCGCCCAACTCGCCATAG |
| WCS171 | FlcD | Y220F | F | GGAAATCGCC**TTC**CTGAACAATCG |
| WCS172 | FlcD | Y220F | R | TCGGGCATCGTCGACACGGCC |
| WCS162 | FlcD | R224A | F | CTGAACAAT**GCT**GCCCGTGCCTTC |
| WCS163 | FlcD | R224A | R | GTAGGCGATTTCCTCGGGCAT |
| AP5 | FlcD | E244A | F | CTACATCACC**GCG**CTGGTCGTGCC |
| AP6 | FlcD | E244A | R | AACACCGCCAGCCCCCAACCGAC |
| AP17 | FlcD | H251A | F | GTGCCGGGGAAC**GCC**GAAAAGCTCTATC |
| AP18 | FlcD | H251A | R | GACCAGTTCGGTGATGTAGAACAC |
| AP11 | FlcD | Y270F | F | CCAGGCGGAG**TTC**TACAAGGTCCAC |
| AP12 | FlcD | Y270F/Y271F | R/R | TCCTCGCTCAGGCCGGCCTGGAG |
| AP13 | FlcD | Y271F | F | CCAGGCGGAGTAC**TTC**AAGGTCCAC |
| AP7 | FlcD | H274A | F | CTACAAGGTC**GCG**ATCAGCCTCGTAC |
| AP8 | FlcD | H274A | R | TACTCCGCCTGGTCCTCGCTC |
| AP19 | FlcD | R281A | F | CTCGTACCGCCG**GCC**GCCAAGCGCGAATG |
| AP20 | FlcD | R281A | R | GCTGATGTGGACCTTGTAGTACTC |

**Table S4. Average Mössbauer parameters.**

| | Average Mössbauer parameters | |
|---|---|---|
| **Sample** | Isomer shift (mm/s) | $\Delta E_Q$ (mm/s) |
| **WT Anaerobic** | 1.22 | 2.74 |
| **WT 10 ms** | 1.30 | 3.03 |
| **WT 500 ms** | 1.30 | 2.95 |
| **WT 30 s** | 1.26 | 2.76 |
| **WT 5 min** | 1.24 | 2.76 |
| **WT 4 h** | 1.22 | 2.74 |

**Supplemental Figures:**

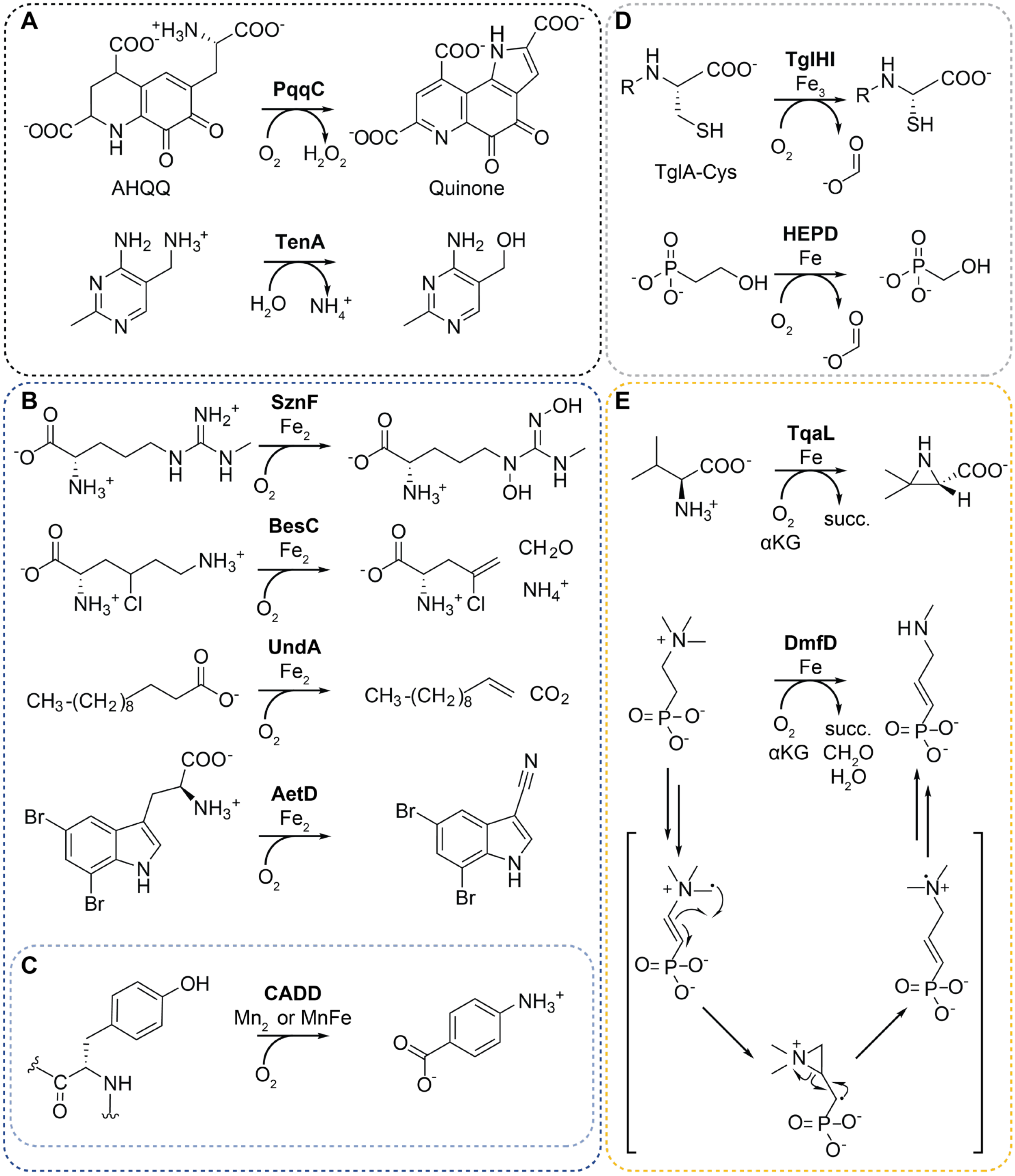


**Figure S1. Reactions discussed in main text.** Reactions of A) cofactor-less heme oxygenase-like enzymes,[15-21] B) iron dependent HDOs,[22-25] C) Mn/Fe dependent HDO,[26-28] D) methine excising enzymes,[18-20, 29, 30] and E) enzymes that are proposed to use an aziridium intermediate (DmfD) or that catalyze the formation of an aziridine product (TqaL).[31-33] α-KG, α-ketoglutarate; succ., succinate.

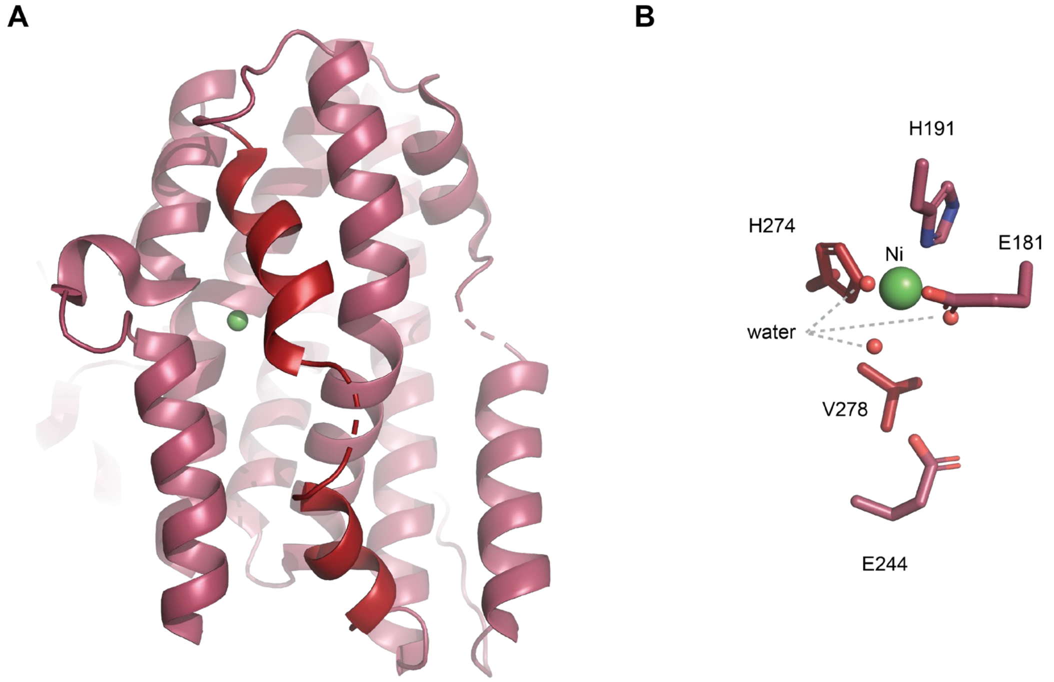


**Figure S2. FlcD structure bound to nickel.** A) Monomer A of FlcD•Ni (PDB:3BJD). Metal binding residues (sticks), nickel (green), partially folded α3 helix (bright red). B) FlcD•Ni active site. E181, H191, and H274 coordinate Ni in addition to three waters. R281 is not resolved in this structure.

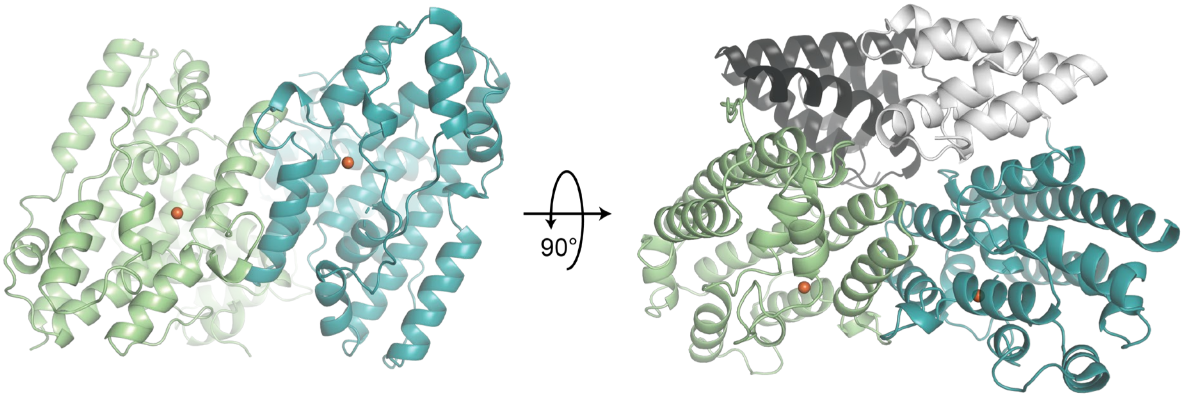


**Figure S3. Tertiary and quaternary structure of FlcD•Fe.** Two views of the FlcD•Fe structure (PDB: 9B9M) rotated 90°. HDO domain (lime green) and N-terminal domain (black) in monomer C interact with HDO domain (teal) and N-terminal domain (white) in monomer D, respectively. Iron in each monomer is shown (orange sphere).

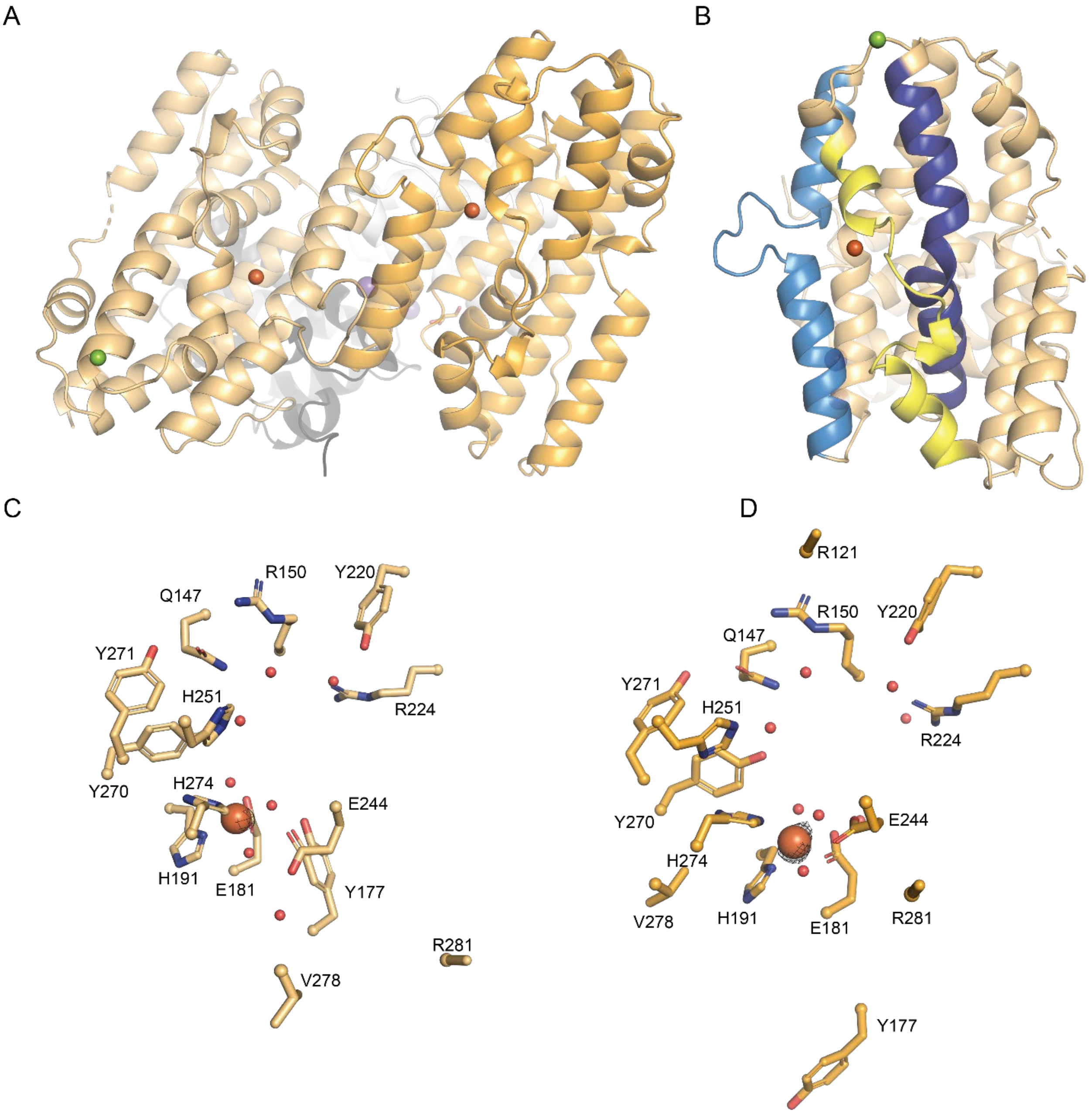


**Figure S4. Aerobic FlcD•Fe structure obtained by growing the crystal in Fe(II)-containing solution.** A) Dimer of FlcD•Fe (PDB: 8W1Q) showing monomer A (light orange/black) and monomer B (orange/white). B) View of one FlcD•Fe monomer. The α1 (light blue), α2 (dark blue) and α3 (yellow) helices are highlighted. In the absence of substrate, the α3 helix is not fully folded. C) Active site of FlcD•Fe monomer A. D) Active site of FlcD•Fe monomer B. Iron (orange), magnesium (lime green), sodium (purple), and water (small red spheres) are shown. Anomalous signal of iron (black mesh) is shown at 3.0 σ. The side chain of R281 is not resolved.

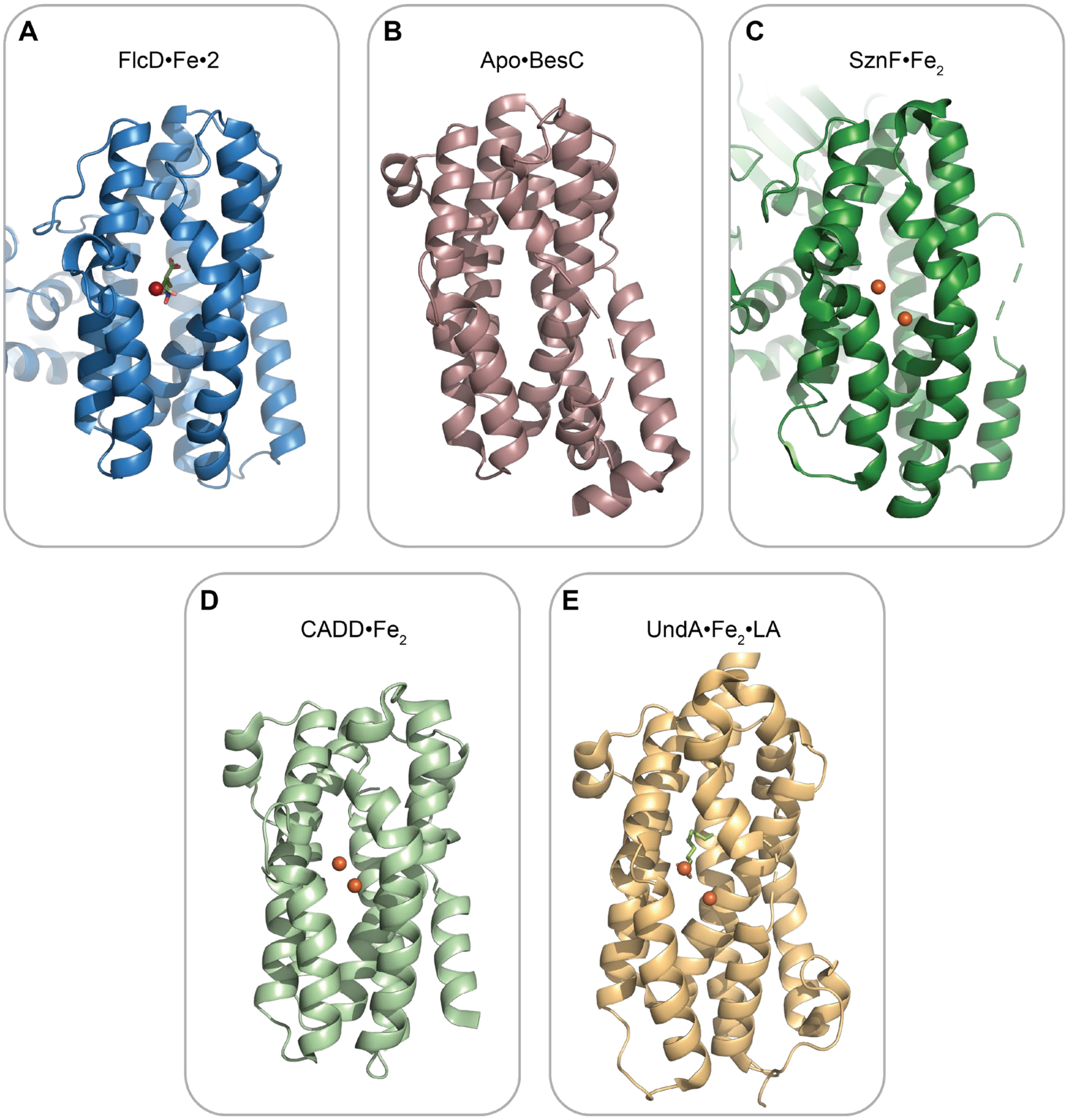


**Figure S5. Comparison of the FlcD•Fe•substrate structure with other HDOs.** Only one monomer of each HDO dimer is shown. A) FlcD•Fe•substrate (PDB: 9B9N) B) Apo-BesC (PDB: 7TWA),[34] C) SznF•$Fe_2$ (PDB: 6VZY),[35] D) CADD•$Fe_2$ (PDB: 1RCW),[36] and E) UndA•$Fe_2$•lauric acid (PDB: 6P5Q).[37] Lauric acid (LA), green. SznF•$Fe_2$ and CADD•$Fe_2$ both have a fully folded α3 helix in the presence of $Fe_2$, while α3 helix in UndA•$Fe_2$• lauric acid contains some disorder. Iron, orange.

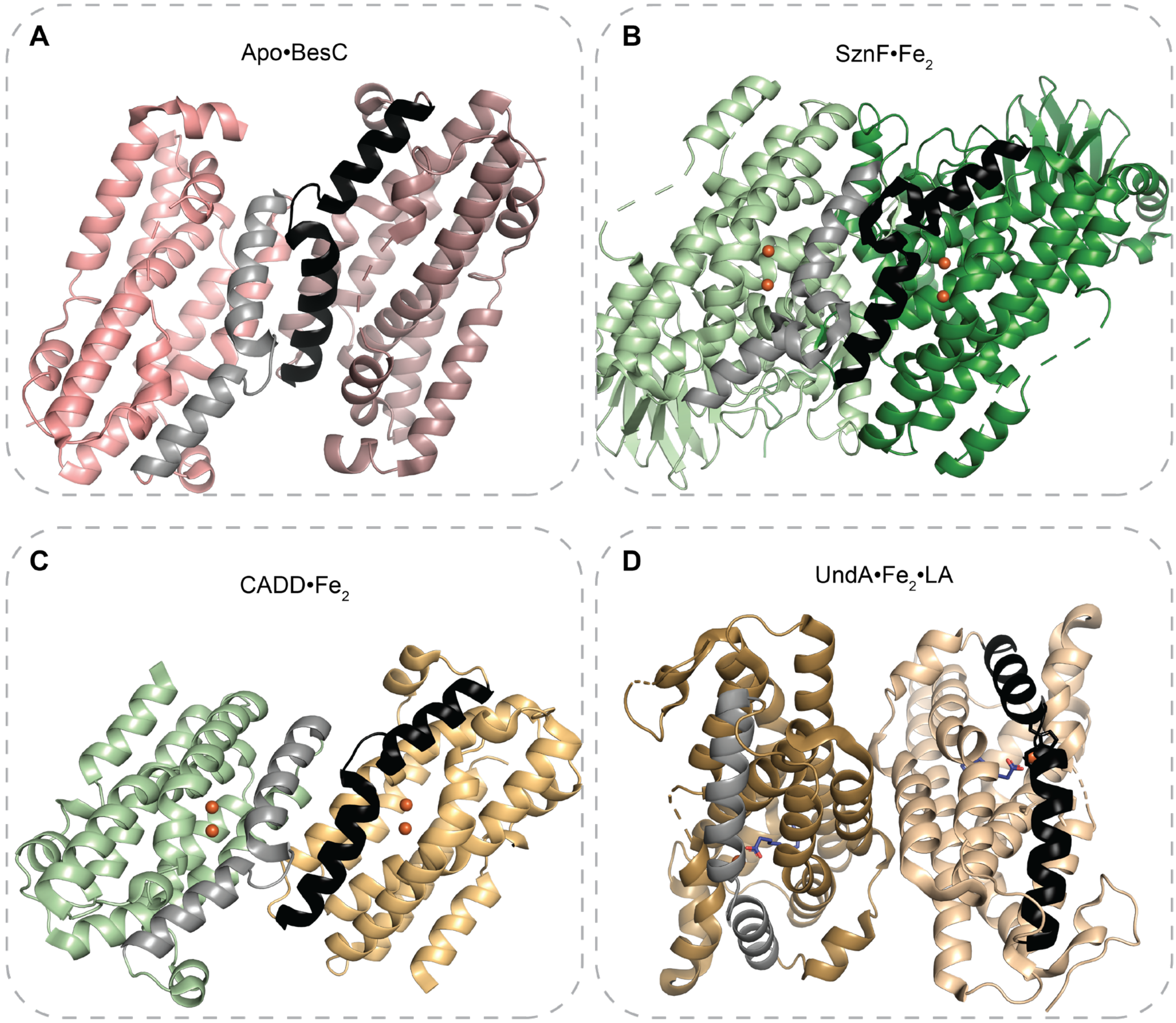


**Figure S6. Characterized HDOs crystalize as dimers.** Dimeric structures of HDOs are shown. A) Apo-BesC (PDB: 7TWA),[34] B) SznF•$Fe_2$ (PDB: 6VZY),[35] C) CADD•$Fe_2$ (PDB: 1RCW),[36] and D) UndA•$Fe_2$•lauric acid (LA) (PDB: 6P5Q).[37] The α1 helix (gray and black) is located at dimer interface of BesC, SznF, and CADD but not UndA.

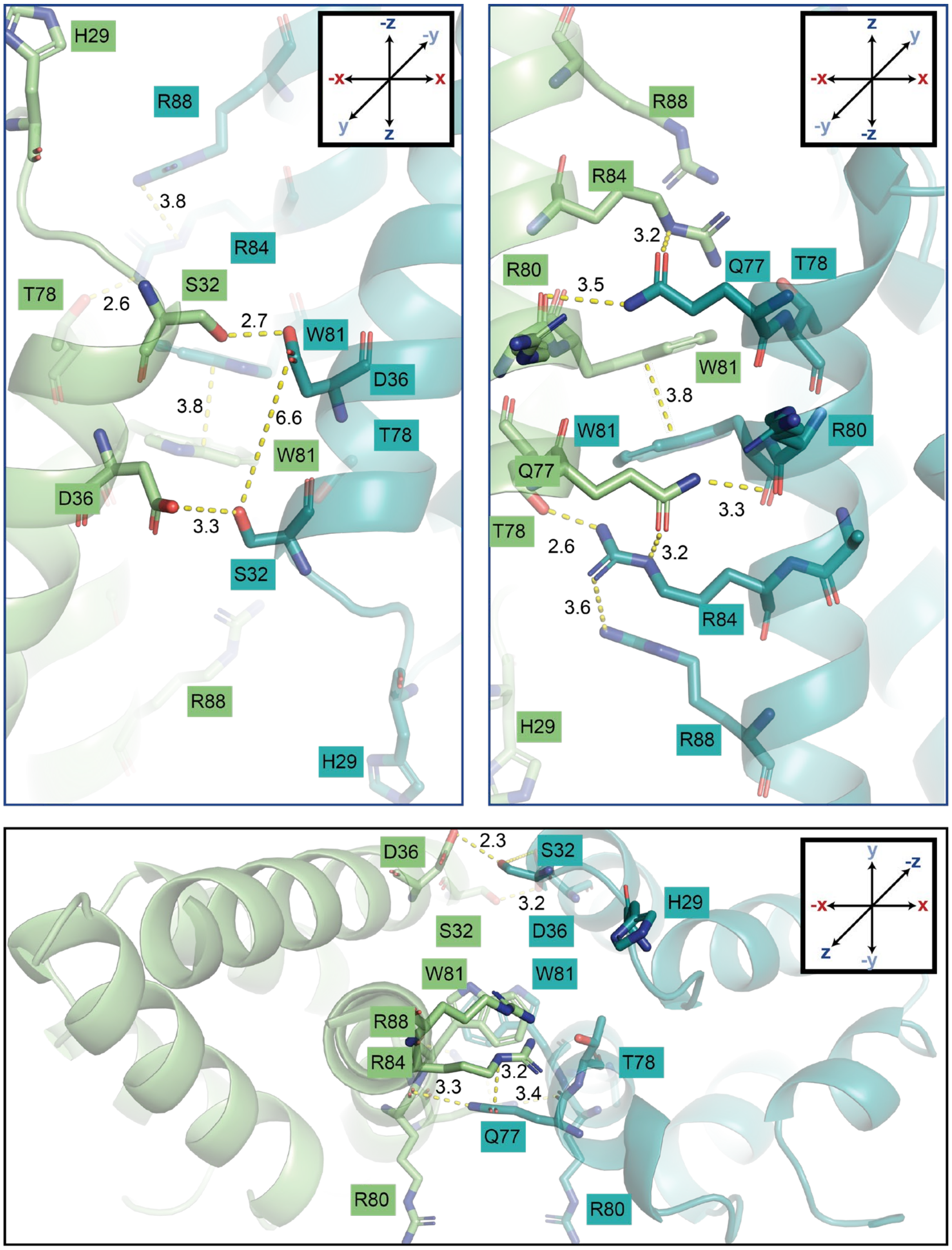


**Figure S7. The N-terminal domains of FlcD•Fe monomers exhibit electrostatic and hydrophobic interactions with each other.** Views of the interactions between the N-terminal domains of the FlcD monomer C (green) and D (teal) (PDB: 9B9M). Distances are labeled in angstroms (Å). Coordinate diagram in each panel (top right) shows the relative orientation of each panel. Similar interactions are observed in the other FlcD structures (PDB: 9B9O, 9B9N, and 8W1Q). Polar, charge-charge, and hydrophobic interactions are shown as yellow dotted lines.

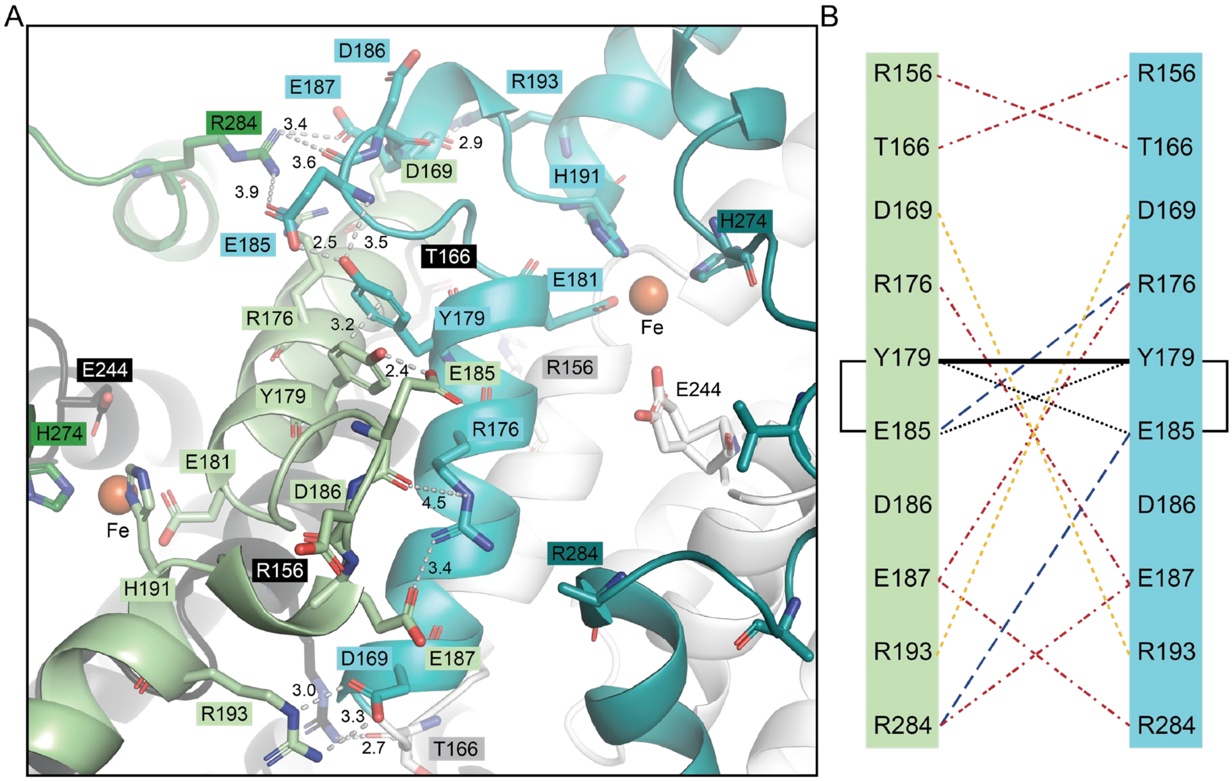


**Figure S8. The HDO domains of FlcD•Fe monomers exhibit extensive interactions with each other.** A) Views of the interactions between the HDO domains of the FlcD monomer A (green) and B (teal) (PDB: 9B9M). Interaction distances are labeled. The α1 helix (pale green – monomer C, teal – monomer D) and α3 helix (forest green – monomer C, deep teal – monomer D) contribute multiple favorable interactions between the two monomers. B) Schematic of the interactions shown in (A) between residues from monomer C (pale green bar) and monomer D (teal bar). Colored lines depict the interactions between these residues (arbitrary colors are used to track the lines). Similar interactions are observed in the other FlcD structures (PDB: 9B9O, 9B9N, and 8W1Q).

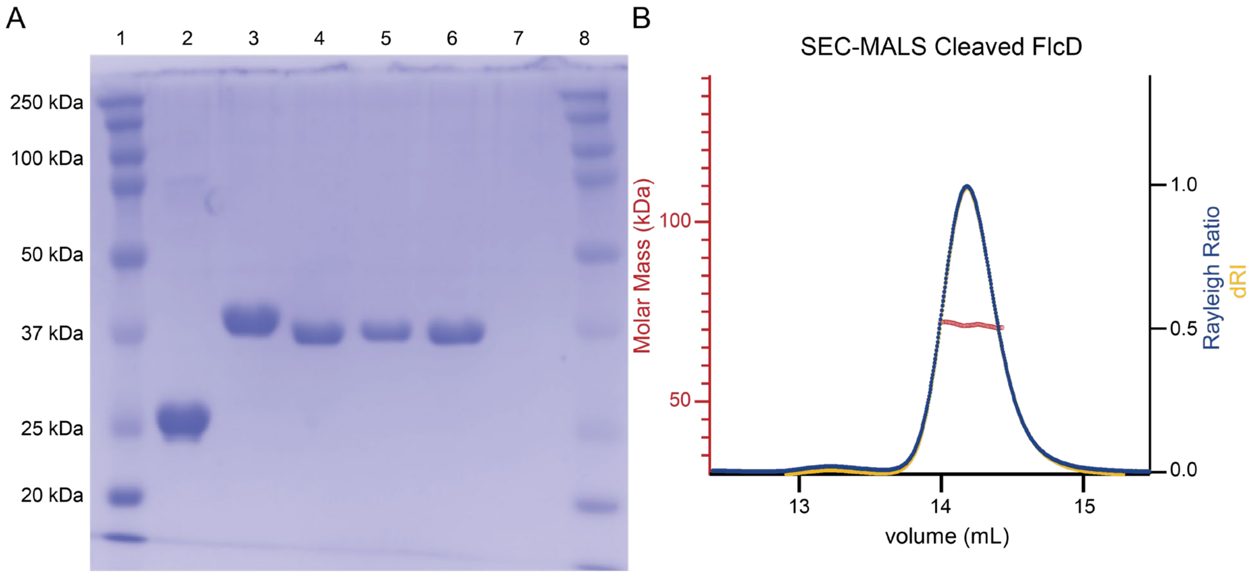


**Figure S9. FlcD is a stable homodimer.** A) Coomassie stained SDS-PAGE gel. Lanes contain ladder (1,8), TEV-$His_6$ protease (2), $His_6$-TEV-FlcD (3), crude TEV cleavage reaction of $His_6$-TEV-FlcD (4), and purified $FlcD_{tagless}$ (5). A sample of purified $FlcD_{tagless}$ (5) was loaded on a 5 mL HisTrap nickel affinity column. The concentrated flowthrough (6) and imidazole elution (7) were analyzed to verify complete cleavage of $His_6$-TEV-FlcD, noted by the absence of a band (7). B) Representative SEC-MALS trace of purified $FlcD_{tagless}$. The weight average molecular weight of $FlcD_{tagless}$ ($M_w$, red line) is 71531 Da (mean of two replicates, expected 37792 Da for monomer and 75584 Da for dimer). $FlcD_{tagless}$ exhibits a number average molecular weight ($M_n$) of 71516 (mean of two replicates). Using the dispersity index (*Đ*) formula $Đ = \frac{M_w}{M_n}$, the average *Đ* is calculated to be 1.0002, indicating $FlcD_{tagless}$ is a monodispersed homodimer. The differential refractive index (dRI, yellow line) shows where $FlcD_{tagless}$ elutes and the normalized Rayleigh ratio (scattered to incident light intensity, blue line) shows maximal scattering during the $FlcD_{tagless}$ elution (dRI maxima). SEC-MALS was conducted in technical duplicate.

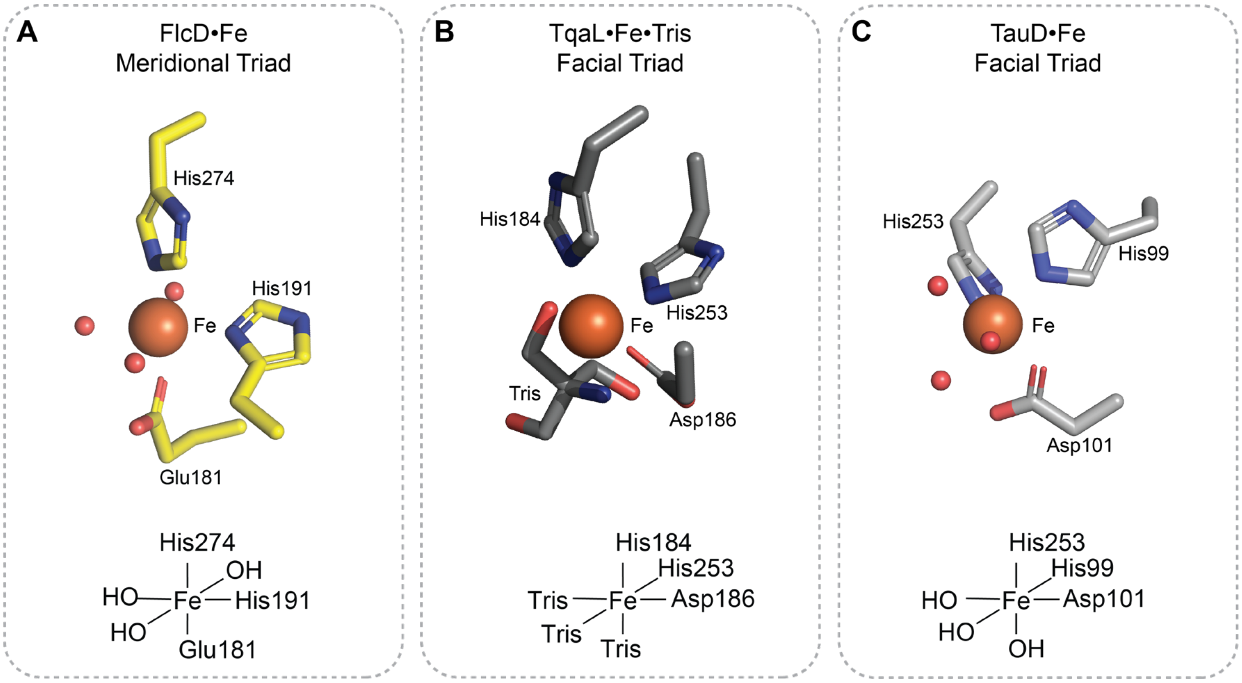


**Figure S10. Iron coordination geometry of FlcD is distinct from that of Fe/α-ketoglutarate-dependent enzymes.** A) Active site of FlcD•Fe (PDB: 9B9M). The 2-His-1-carboxylic triad exhibits meridional coordination geometry. B) Active site of the Fe/α-ketoglutarate-dependent oxygenase, TqaL (PDB: 7EEH),[31] with iron and Tris bound. C) Active site of the Fe/α-ketoglutarate dependent taurine dioxygenase, TauD (PDB: 3R1J).[38] Both TqaL and TauD contain a 2-His-1-carboxylic facial triad representative of the iron-binding mode in Fe/α-ketoglutarate oxygenases. Iron (orange), water (red).

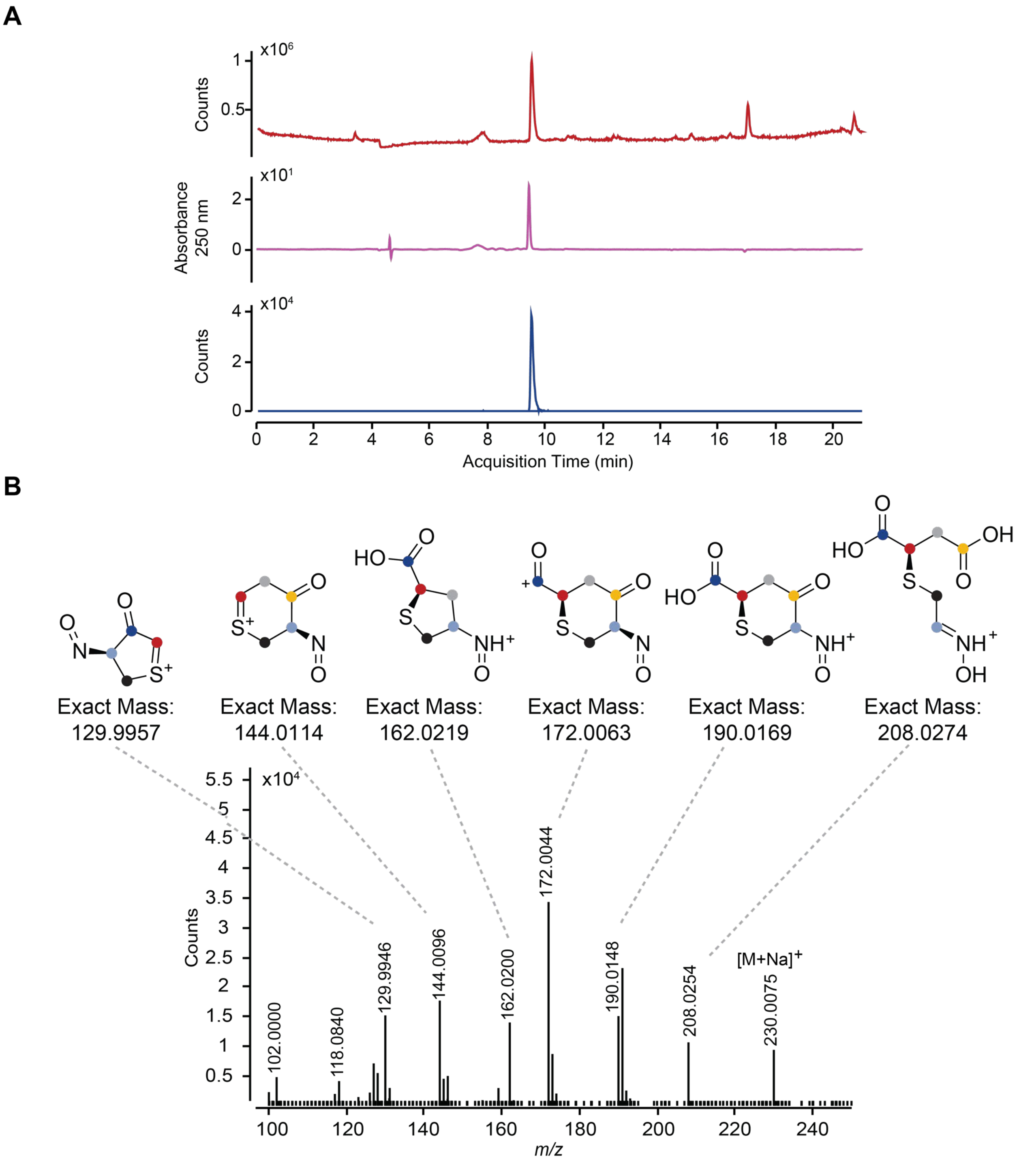


**Figure S11. LC-HRMS analysis of the purity of the FlcD substrate, 2.** A) Representative blank subtracted total ion chromatogram (TIC), UV 250 nm, and extracted ion chromatogram (EIC) of purified **2** (*m*/z $[M + H]^+ = 208.0274$). B) Representative blank subtracted mass spectra of **2** at 9.5–10 min retention time. Proposed structures for the in-source fragmentation ions are shown. Colored dots are used for tracking carbon atoms. LC-HRMS analysis was performed using electrospray ionization under positive ion mode.

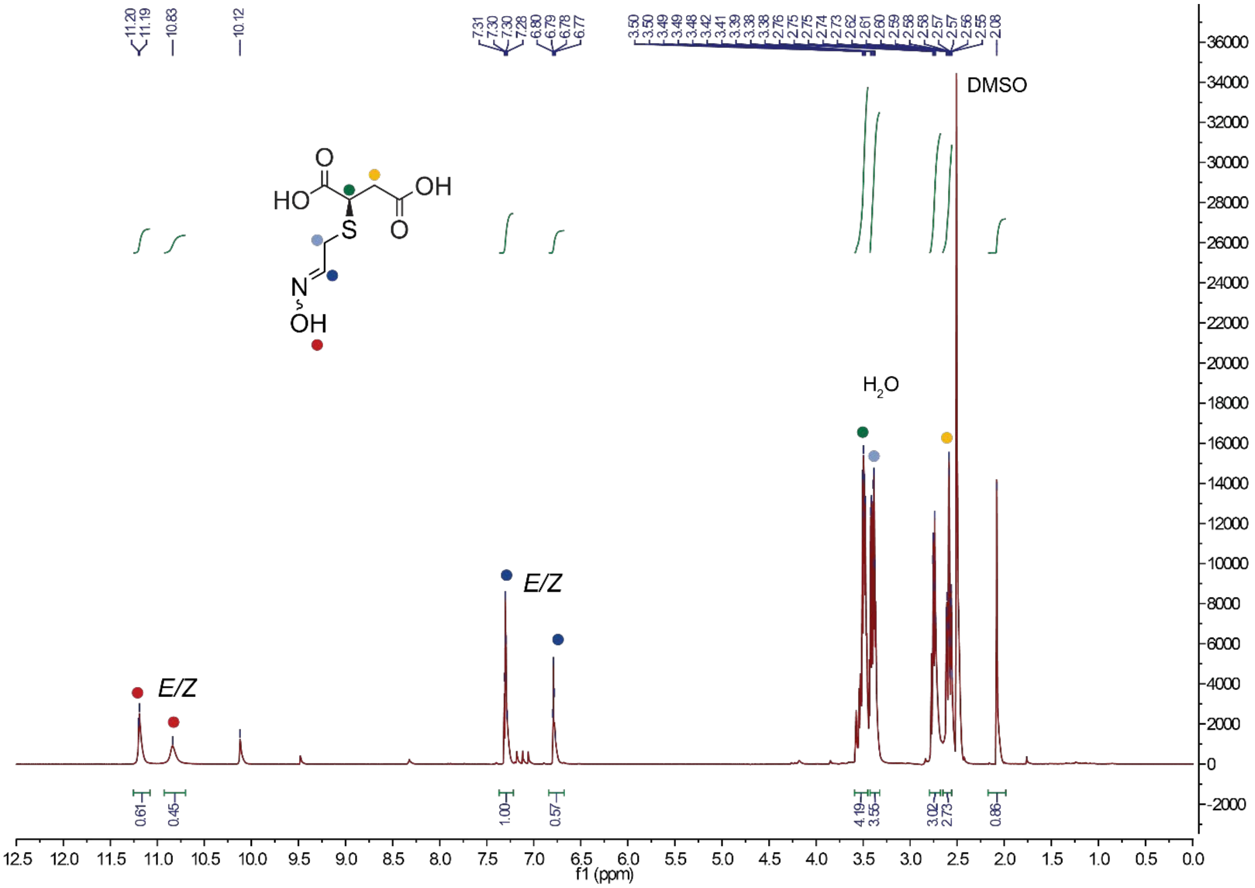


**Figure S12. Representative $^1$H NMR spectrum of purified 2 in $D_6$-DMSO (850 MHz).**

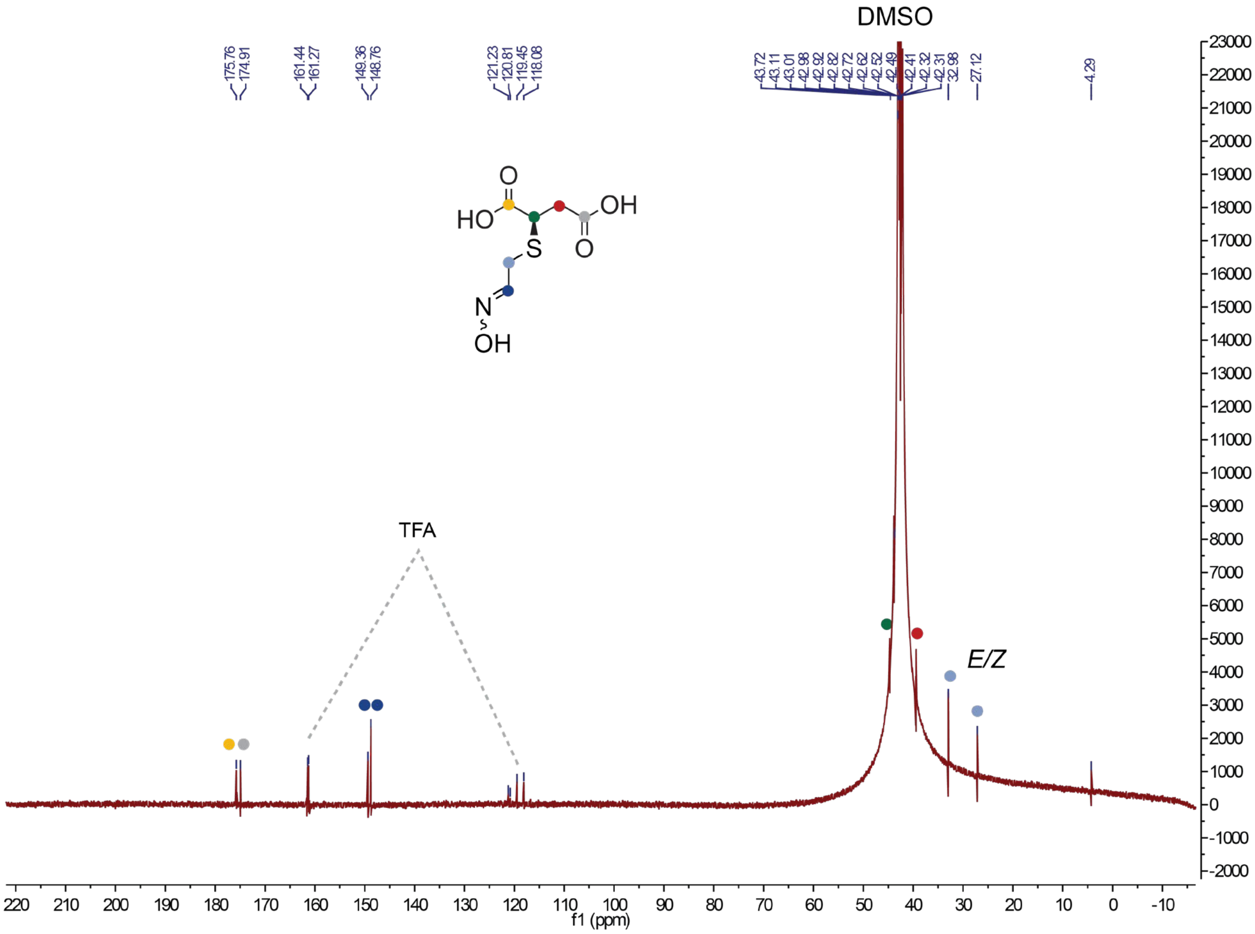


**Figure S13. Representative $^{13}$C NMR spectrum of purified 2 in $D_6$-DMSO (850 MHz).**

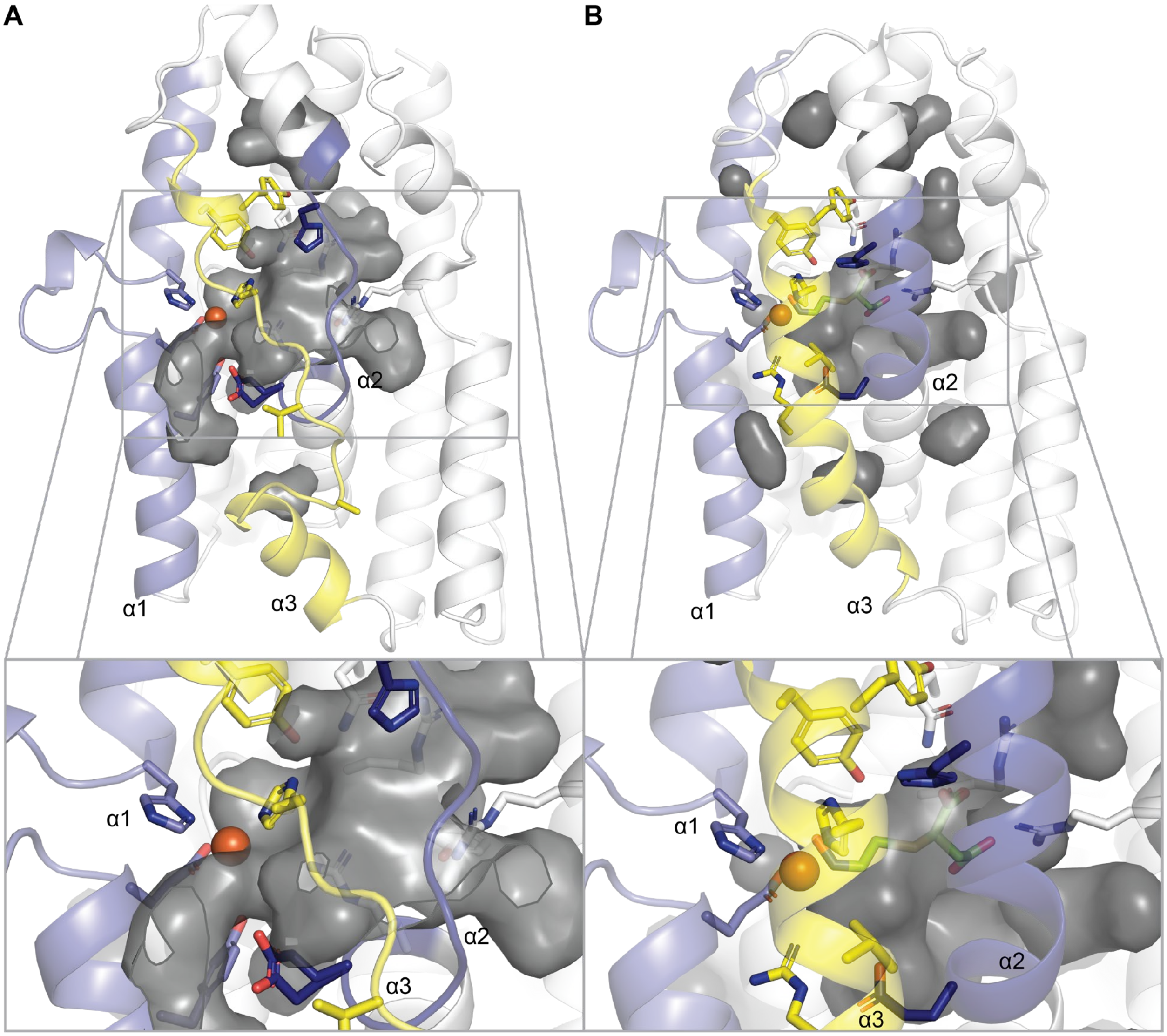


**Figure S14. Active site pocket of FlcD closes upon substrate binding.** A) Monomer D of FlcD•Fe (PDB: 9B9M) contains partial loops in the α2 and α3 helices that leave the active site pocket open in the absence of substrate. B) Binding of substrate (Monomer B of FlcD•Fe•substrate, PDB: 9B9N), **2**, results in the reorganization of the loops of α2 and α3 into complete helices, which closes off the active site pocket. α2 (indigo), α3 (yellow). The active site pocket (dark gray) is detected with a 7 Å radius and cutoff of 8 solvent radii.

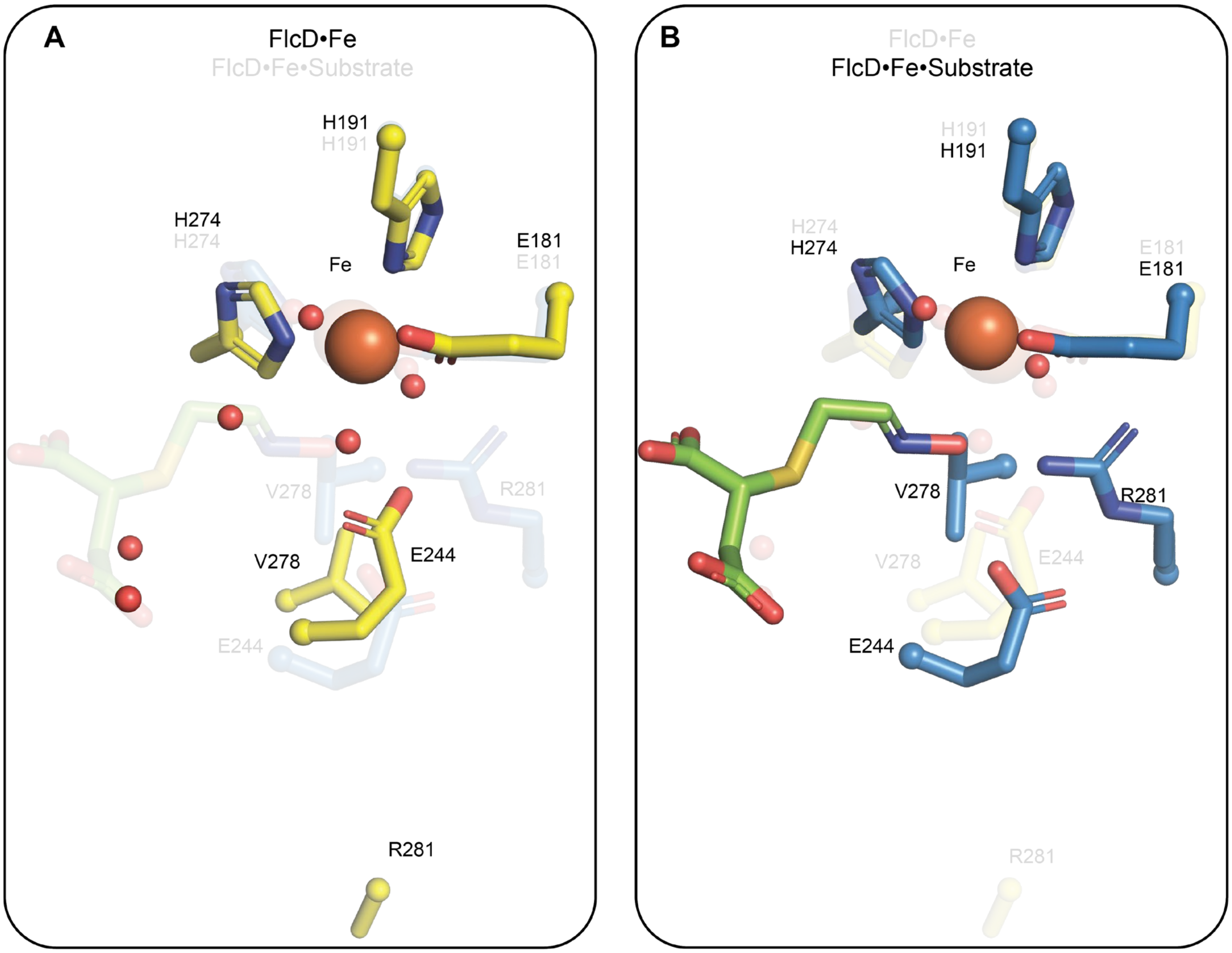


**Figure S15. The substrate replaces waters in monomer C of the FlcD•Fe active site.** Overlay of FlcD•Fe (PDB: 9B9M) and FlcD•Fe•substrate (PDB: 9B9N) structures. The residues that align in sequence with the metal-binding motif in other HDOs are shown in sticks. Fe (orange), water (red), substrate (bright green). A) Focus on the FlcD•Fe structure. B) Focus on FlcD•Fe•substrate showing movement of the active site residues and displacement of two water molecules including an iron-coordinating water by the substrate.

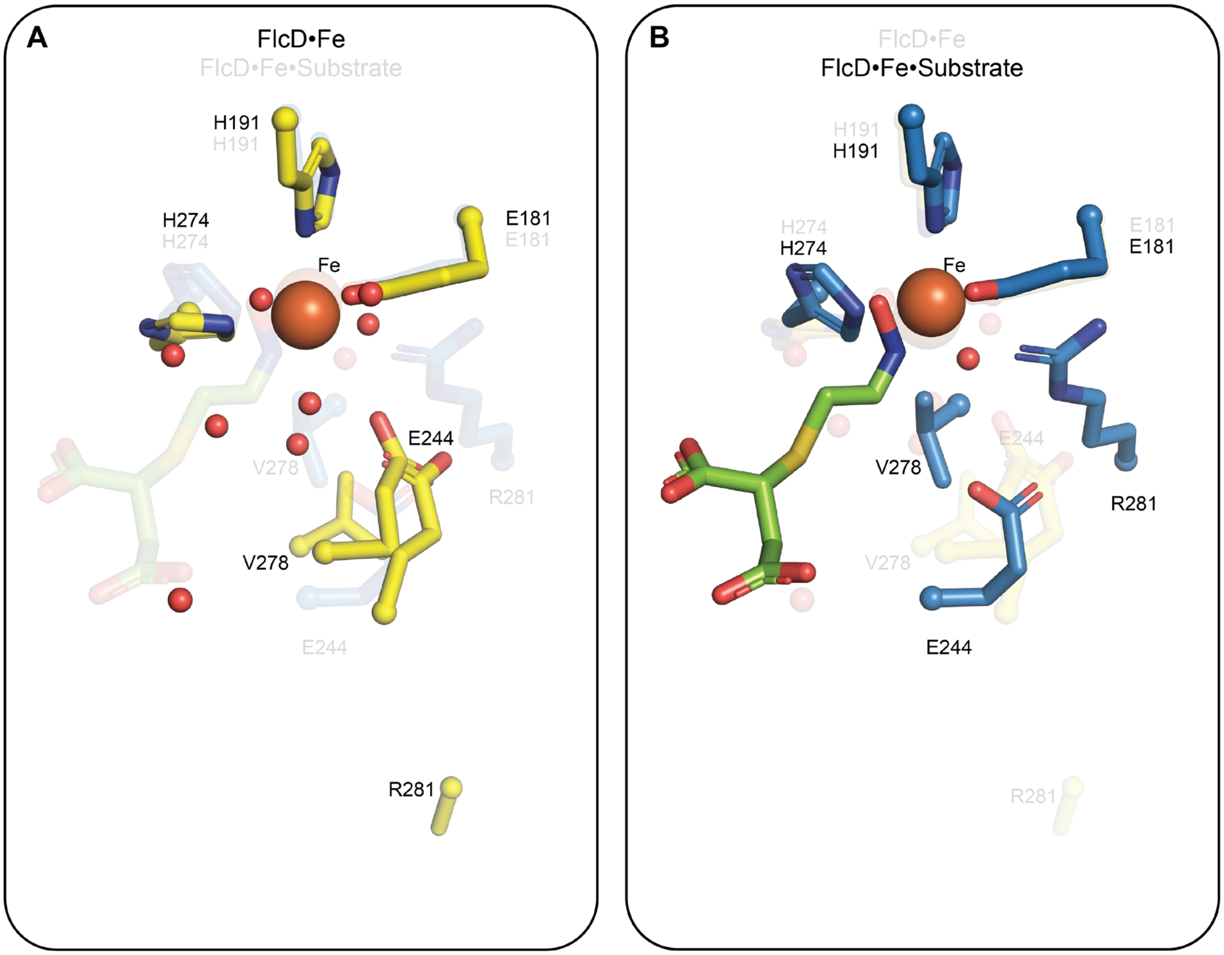


**Figure S16. The substrate replaces waters in monomer D of the FlcD•Fe active site.** Overlay of FlcD•Fe (PDB: 9B9M) and FlcD•Fe•substrate (PDB: 9B9N) structures. The residues that align in sequence with the metal-binding motif in other HDOs are shown in sticks. Fe (orange), water (red), substrate (bright green). A) Focus on the FlcD•Fe structure. B) Focus on FlcD•Fe•substrate showing movement of the active site residues and displacement of an iron-coordinating water by the substrate.

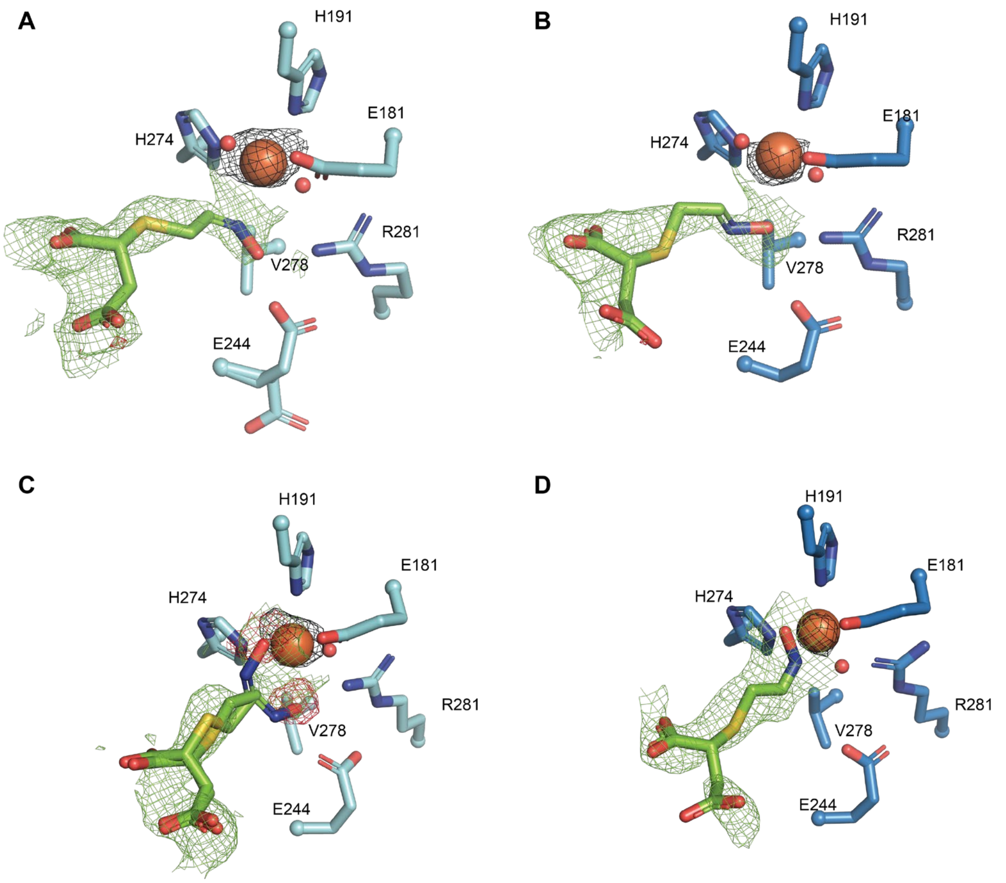


**Figure S17. Two crystals of FlcD•Fe•substrate exhibit similar substrate binding modes.** A) Monomer A of crystal (PDB: 9B9O) shares a similar substrate binding mode to B) monomer A of crystal (PDB: 9B9N) where Fe is coordinated by two waters and the substrate. C) Monomer B of crystal (PDB: 9B9O) shares a similar substrate binding mode to D) monomer B of crystal (PDB: 9B9N) where Fe is coordinated by one water and the substrate oxime. Iron anomalous signal (black mesh) is shown at a σ radius of 3 and carve of 2. The Fo-Fc map (red mesh) for substrate is shown at a 3.0 σ. OMIT map (green mesh) is shown at 1 σ. Two conformations of substrate are modeled in C) (PDB: 9B9O).

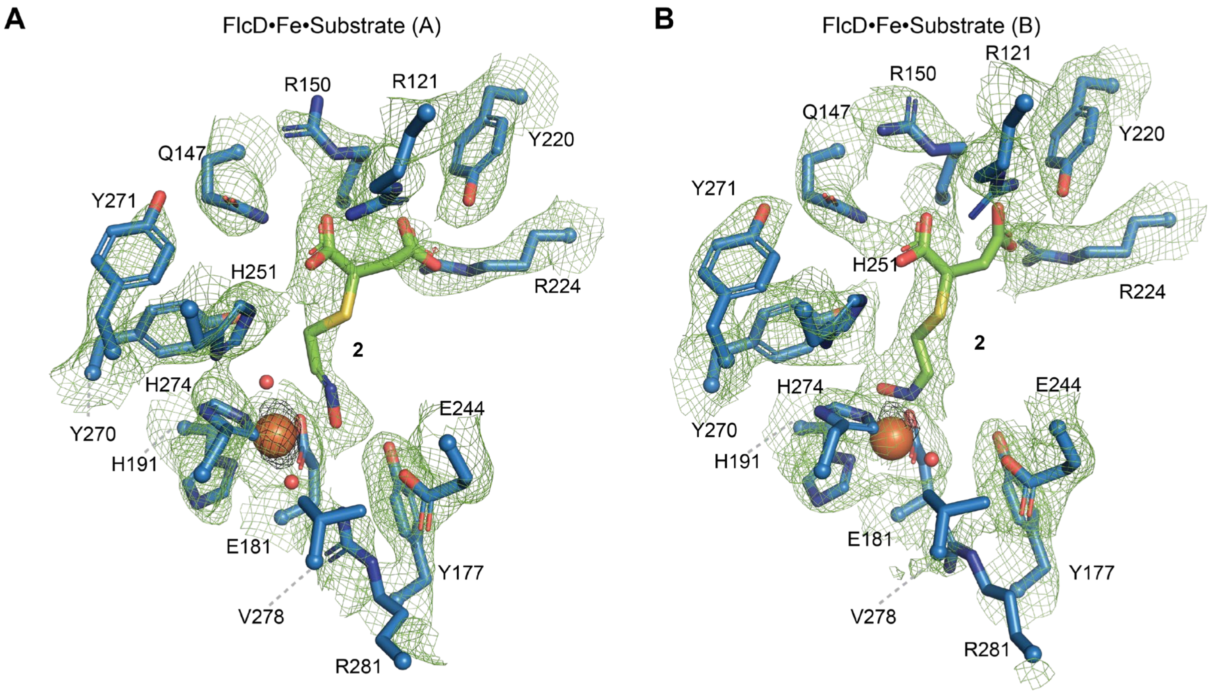


**Figure S18. FlcD•Fe•substrate Fo-Fc, OMIT, and anomalous maps.** A) FlcD•Fe•substrate active site for A) monomer A and B) monomer B (PDB: 9B9N). Fo-Fc (red mesh) map of substrate and anomalous signal of iron (black mesh) is shown at 3 σ, the OMIT (green mesh) at 1 σ. Key residues (sticks) are shown, including iron-binding and substrate-binding residues. Fe (orange), water (red), substrate (green).

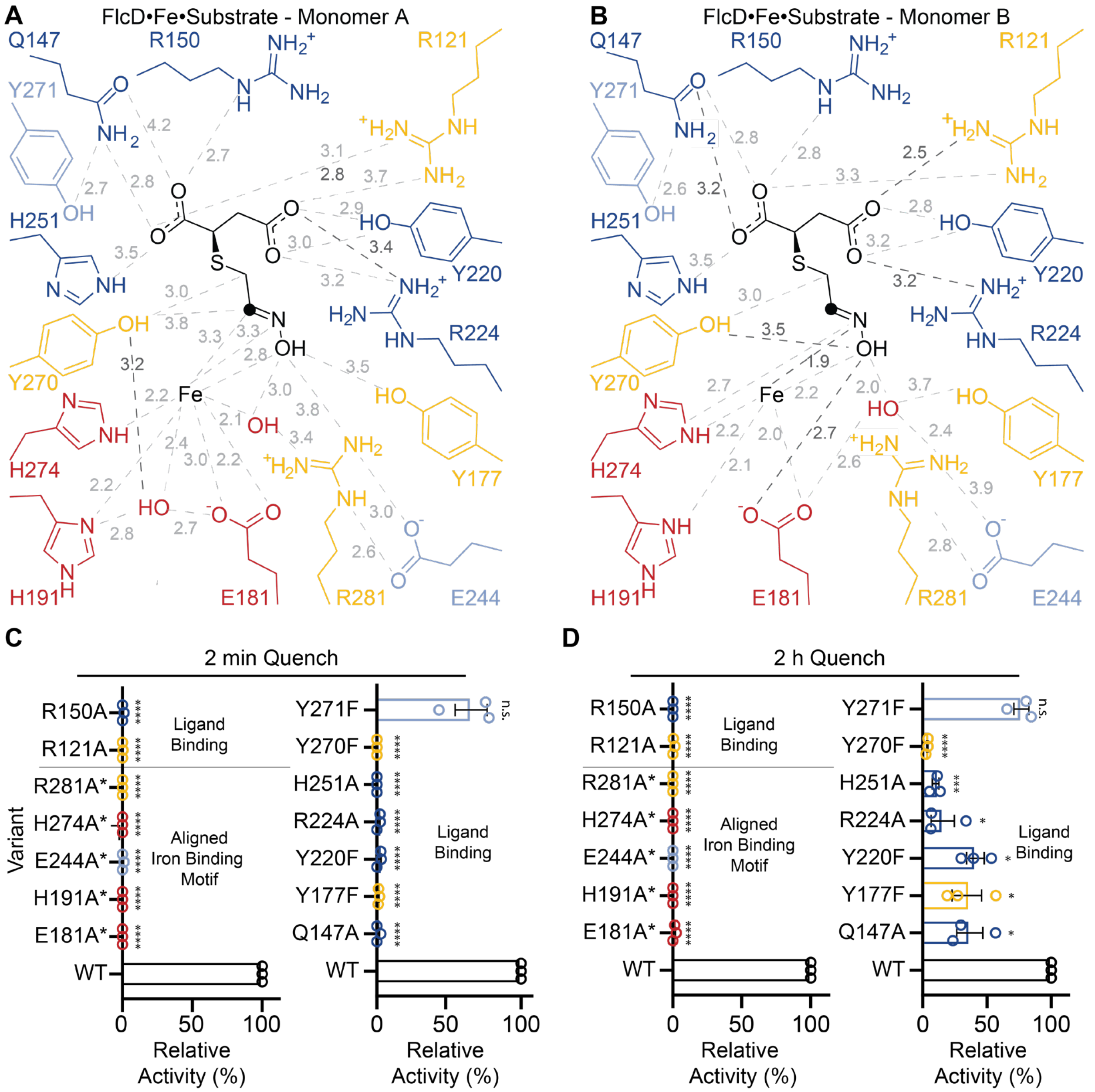


**Figure S19. Active site schematic of the FlcD•Fe•substrate structure and identification of essential residues for enzyme activity.** A) Two-dimensional schematic of FlcD bound to substrate and Fe(II) (PDB: 9B9N) in A) monomer A and B) monomer B. Iron–coordinating residues (red), substrate-coordinating residues (dark blue), residues that change in coordination from monomer A to B (yellow), and residues with no ligand coordination (light blue). Gray dotted lines represent metal-ligand, polar, and charge-charge interactions. C–D) Site-directed mutagenesis studies of FlcD active site residues. Relative activity of each variant to WT enzyme is quantified based on product formation using LC-HRMS. Activities of FlcD WT and variants from C) 2-min reactions and D) 2-h reactions. Data from triplicate experiments are plotted (error bars are standard error of the mean). Colors in C–D are consistent with those in A–B. Excised carbon highlighted (black dot, A–B). Statistical significance was calculated using an uncorrected

Fisher's Least Significant Difference test that compares each variant to wildtype (n.s. = no significant difference, * = P < 0.05, *** = P < 0.001, and **** = P < 0.0001). LC-HRMS analysis was performed using electrospray ionization under positive ion mode.

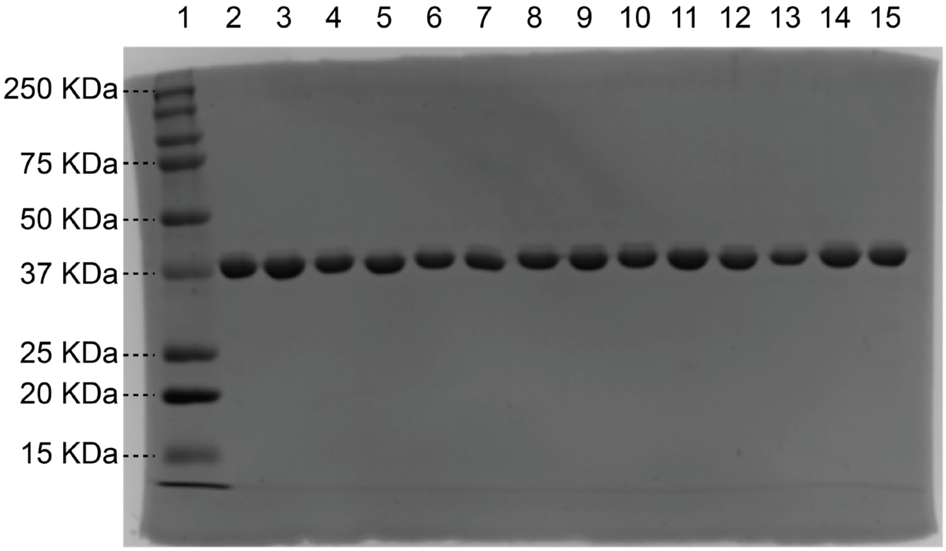


**Figure S20. Purity of FlcD WT and variants assessed by sodium dodecyl sulfate-polyacrylamide gel electrophoresis (SDS-PAGE).** Lanes contained protein standard ladder (1), 5 μM of FlcD WT (2), E181A (3), H191A (4), H274A (5), R281A (6), E244A (7), Q147A (8), R121A (9), R150A (10), Y177F (11), Y220F (12), H251A (13), R224A (14), or Y270F (15).

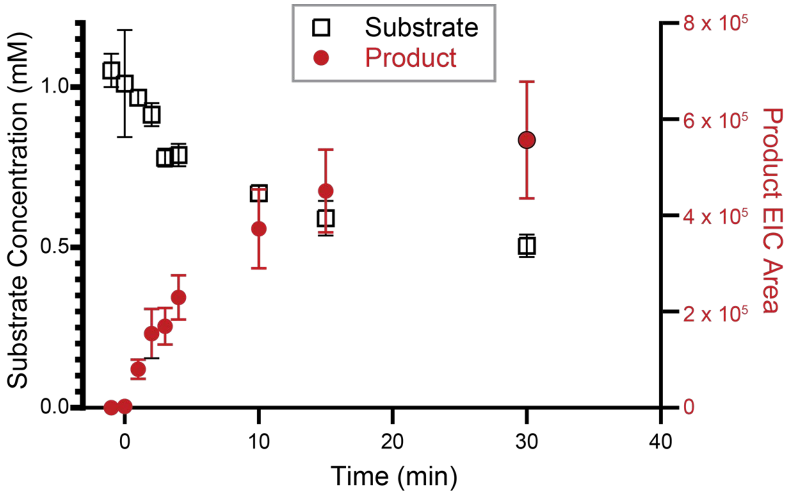


**Figure S21. Time course of FlcD activity over 30 min.** FlcD reaction was conducted to identify the linear range under steady-state conditions. Substrate consumption in mM (black, left y-axis) and product formation in extracted chromatogram area (EIC area) (red, right y-axis) are plotted over time. Values shown are the average of two experiments with standard deviation. FlcD activity measured at 2 min was used to estimate the apparent initial velocity. The −1 min timepoint corresponds to concentrations in the reaction master mix before the addition of FlcD. LC-HRMS analysis was performed using electrospray ionization under positive ion mode.

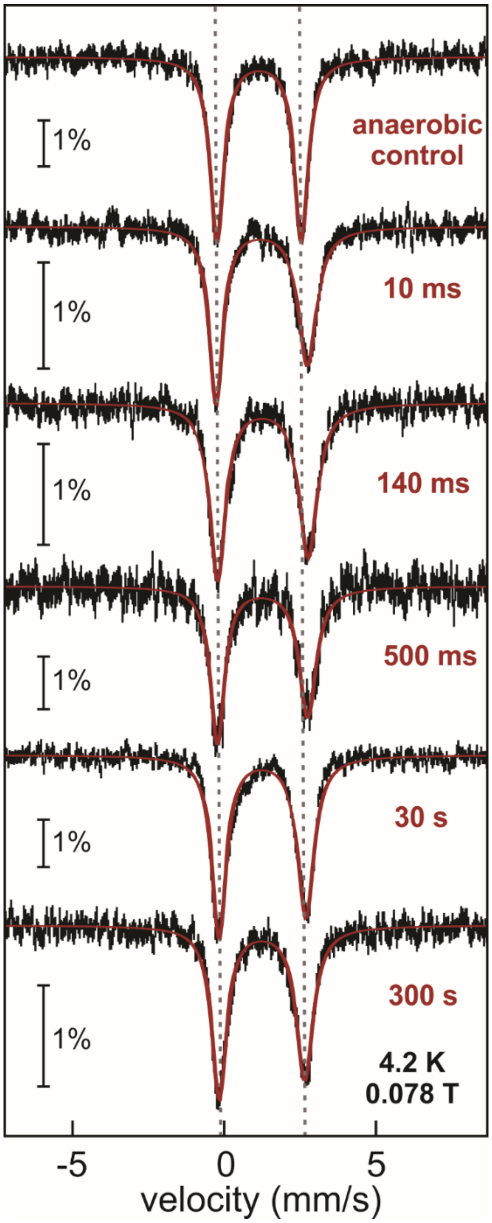


**Figure S22. The 4.2 K Mössbauer spectra of FlcD.** Samples of 1 molar eq of Fe(II) and 3 eq of substrate were added to an anoxic solution of FlcD (1.7 mM) as is or reacted with a solution saturated with $O_2$ (1.8 mM) and quenched at selected time points. The spectra were acquired in the presence of a small external magnetic field (0.078 T) applied parallel to the direction of the $\gamma$-beam. The experimental spectra are shown as black vertical lines, the quadrupole doublets corresponding to the fits of the Fe(II) component are shown as red solid lines.

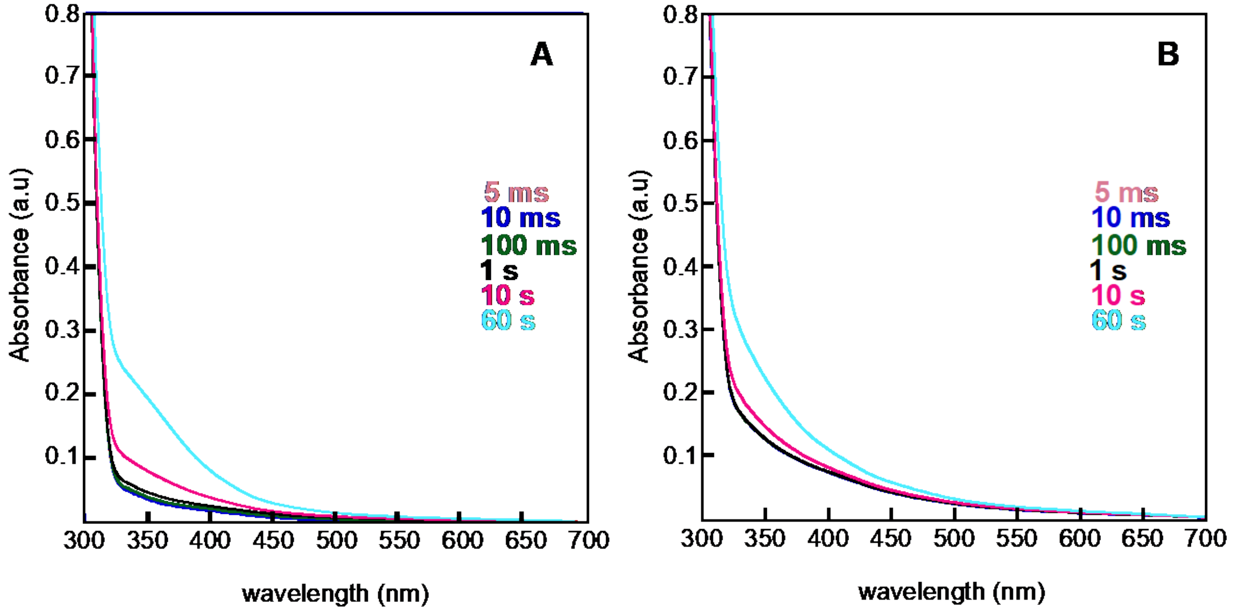


**Figure S23. FlcD single mixing stopped-flow-absorption spectroscopy experiments.** A) A solution containing 0.75 mM FlcD, 7.5 mM Fe(II), and 3 mM substrate, **2**, was reacted in a 1:1 mixing ratio with $O_2$ (1.8 mM) saturated buffer. B) A solution containing 0.5 mM FlcD, 5 mM Fe(II), and 2.5 mM deuterated substrate, [$D_2$]-**2**, was reacted in a 1:1 mixing ratio with $O_2$ (1.8 mM) saturated buffer. The experiments were performed at 5 °C and a photodiode array was used for detection. The Fe(II) in these experiments was used in 10-fold excess to allow for accumulation of any transient optically detectable species in case Fe serves as a co-substrate in the reaction as reported for BesC.[34]

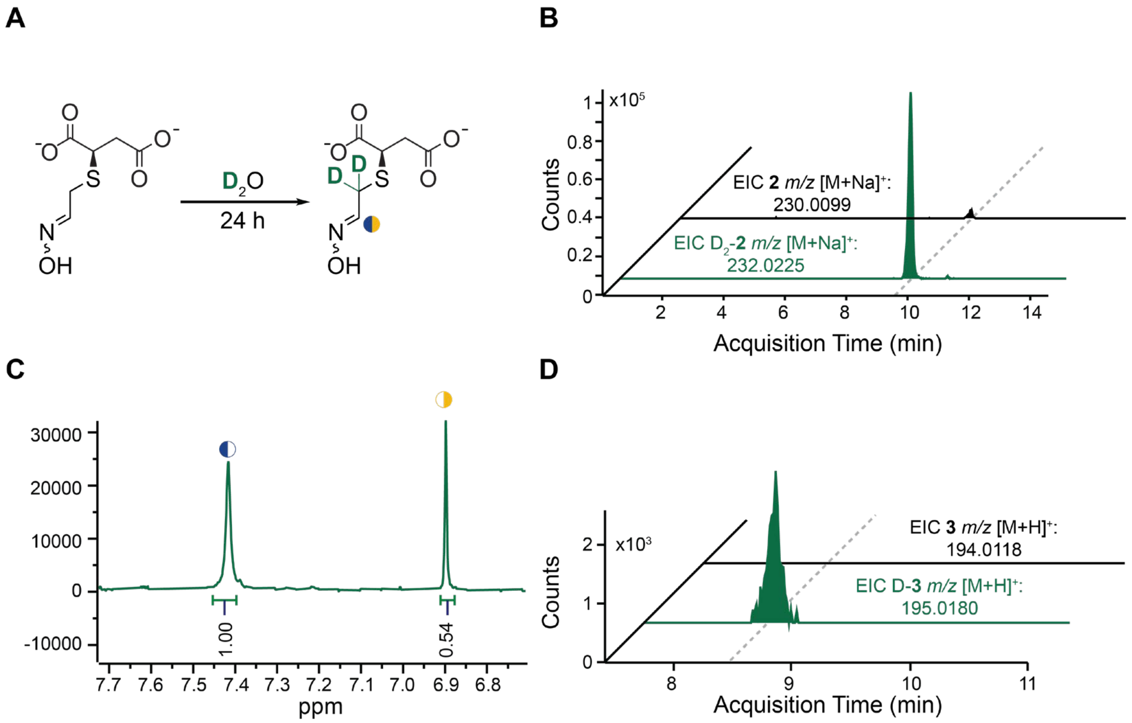


**Figure S24. Preparation of [$D_2$]-2 and its modification by FlcD.** A) Scheme of deuterium exchange of the FlcD substrate, **2**. B) LC-HRMS analysis of **2** after soaking in $D_2O$ for ~24 h. EICs are shown for unlabeled **2** and [$D_2$]-**2**. Minimal amounts of unlabeled species remained. C) $^1$H NMR analysis of [$D_2$]-**2** in $D_2O$. The non-exchangeable oxime proton (blue and yellow circle) was detected as a major (blue half circle) and minor (yellow half circle) species with a loss in multiplicity compared to unlabeled **2**. D) LC-HRMS analysis of a FlcD reaction using [$D_2$]-**2** as substrate. EICs are shown for unlabeled **3** and [D]-**3**. Only [D]-**3** was produced. LC-HRMS analysis was performed using electrospray ionization under positive ion mode.

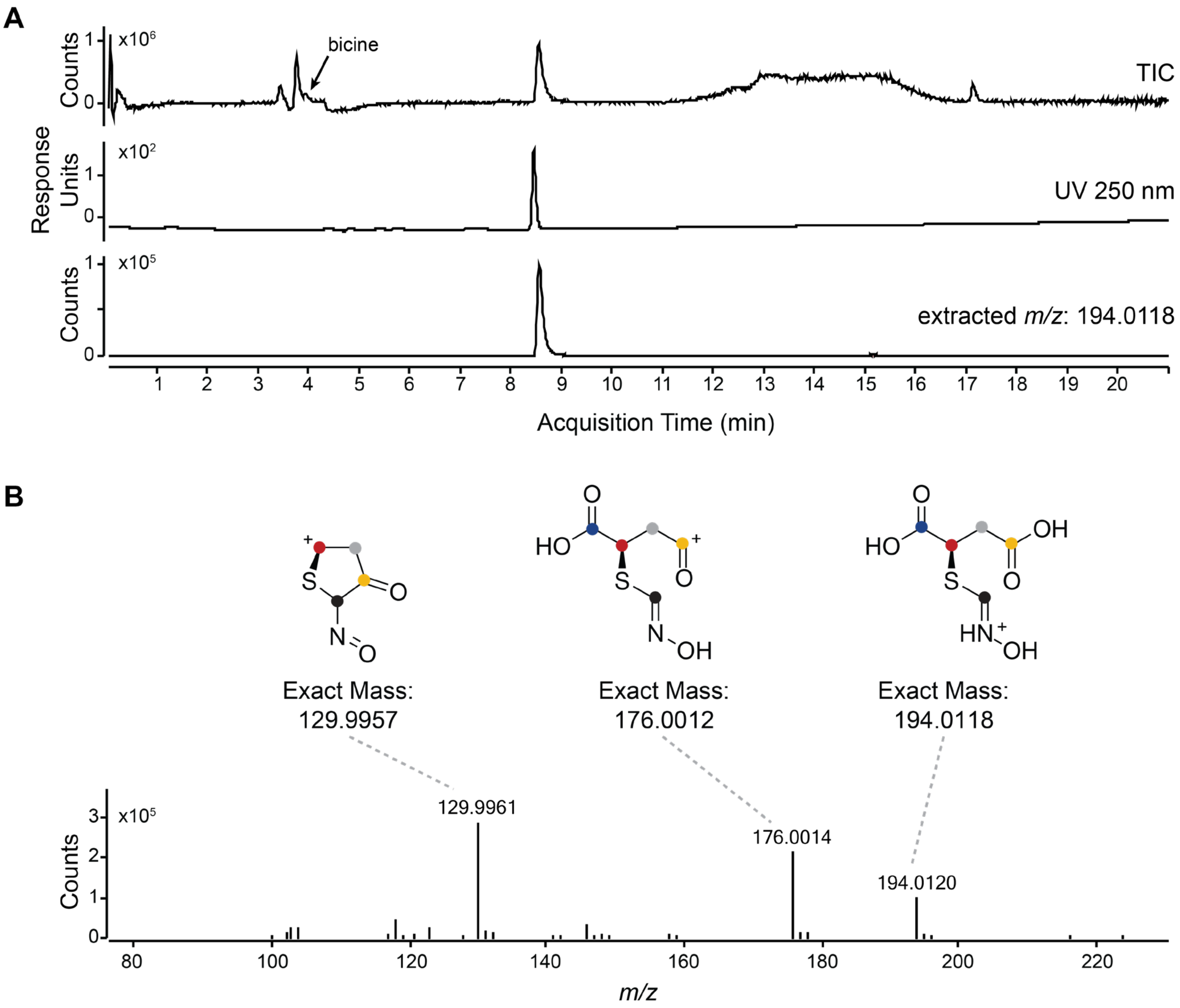


**Figure S25. LC-HRMS analysis of the purity of the FlcD product, 3.** A) Representative blank subtracted total ion chromatogram (TIC), UV 250 nm, and extracted ion chromatogram (EIC) of purified **3** (*m*/z $[M + H]^+$ = 194.0118). B) Representative mass spectra of **3** at 8.5–9 min retention time. Proposed structures for in-source fragmentation ions are shown. Colored dots are used for tracking carbon atoms. LC-HRMS analysis was performed using electrospray ionization under positive ion mode.

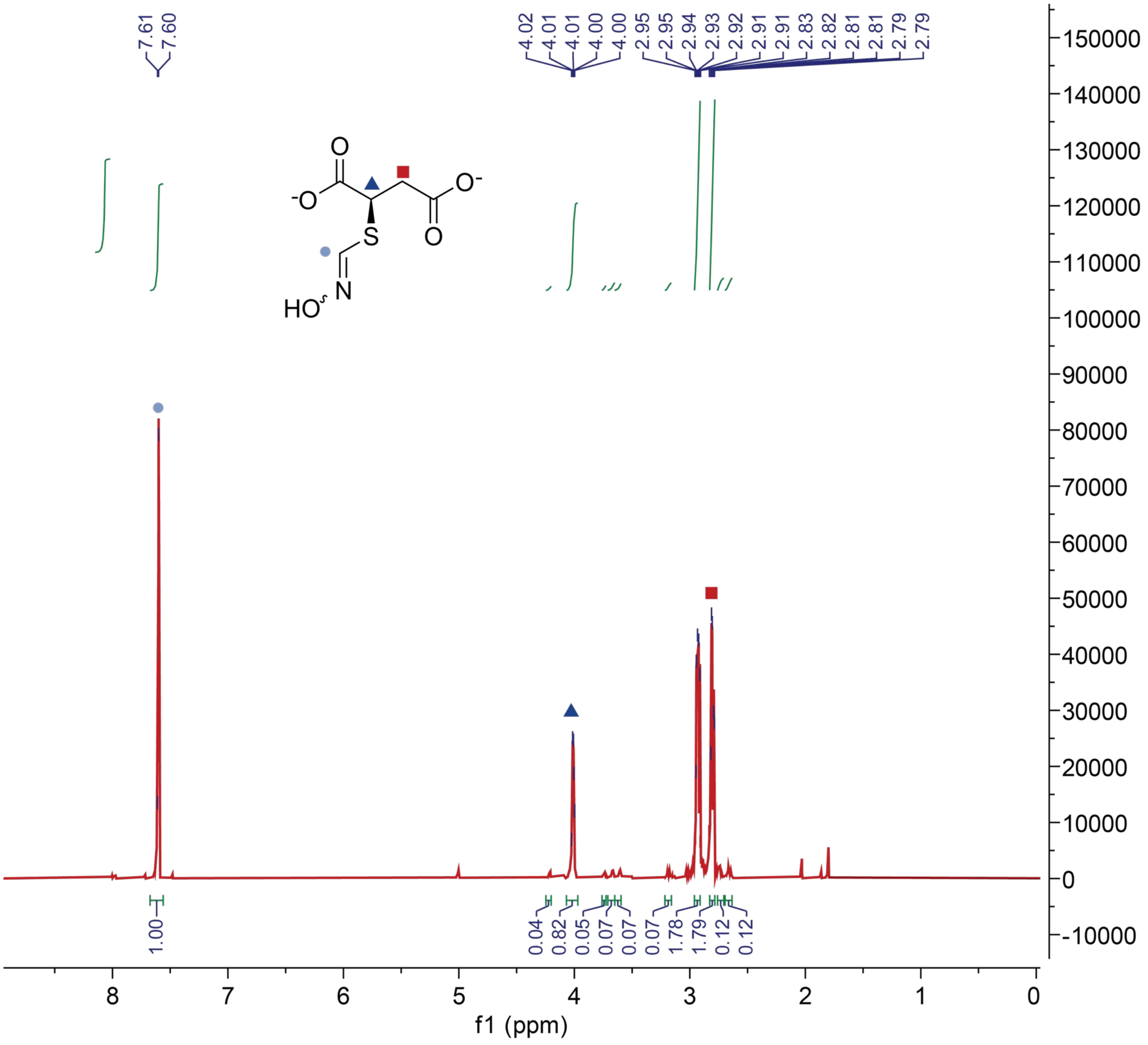


**Figure S26. $^1$H NMR spectrum of purified 3 in $D_2O$ (850 MHz).** The peaks at 2.9 ppm likely correspond to impurities from bicine and HEPES buffers.

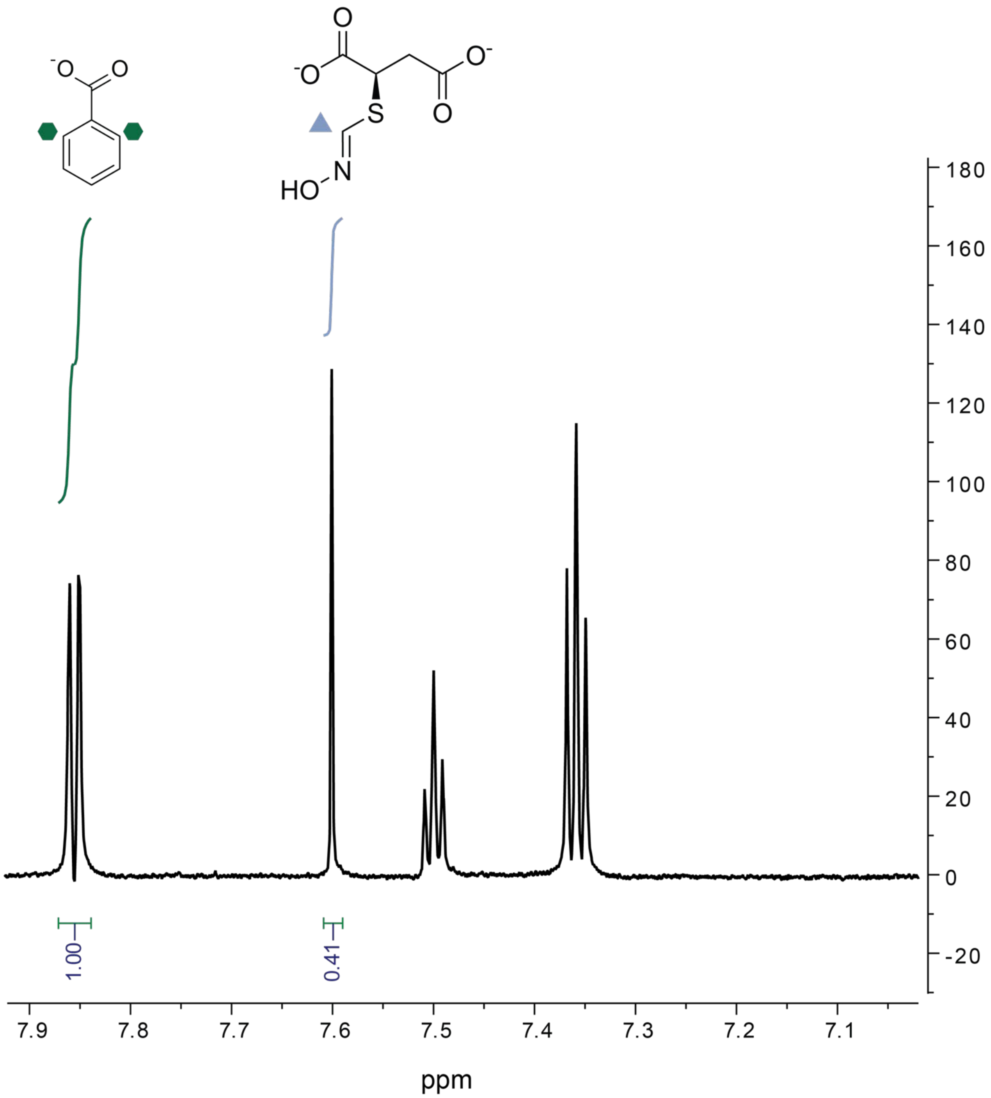


**Figure S27. $^1$H NMR spectrum of 1 mM purified 3 in $D_2O$ with 1 mM of benzoic acid as an internal standard (850 MHz).** The purity of FlcD product is estimated by quantifying the integrated peak areas of the protons of benzoic acid (green hexagons) and the oxime proton in **3** (blue triangle). Product purity is estimated to be 82%.

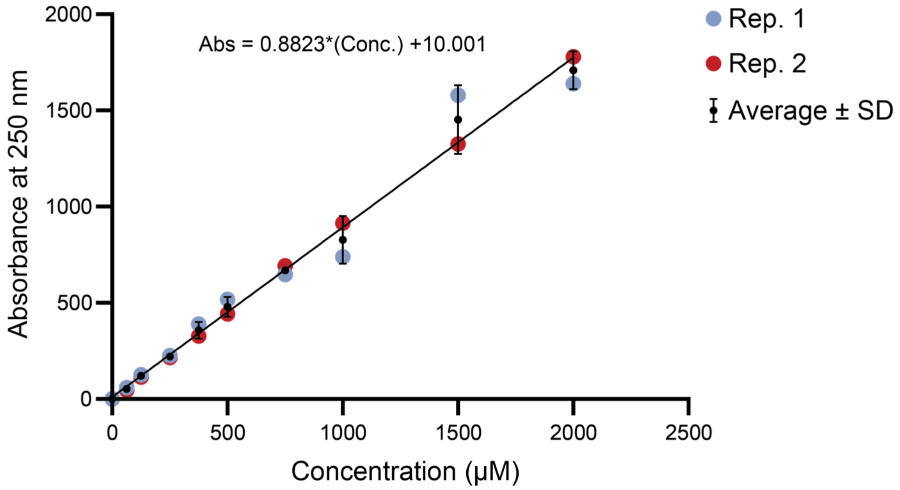


**Figure S28. Standard curve of FlcD product, 3.** The absorbance of varying concentration of a FlcD product standard, **3**, was measured on LC. Peak integration of absorbance at 250 nm is plotted as a function of product concentration in duplicate (blue and red). The average of two replicates was analyzed using linear regression (black). Electrospray ionization MS analysis under positive ion mode was used to verify the retention time of FlcD product.

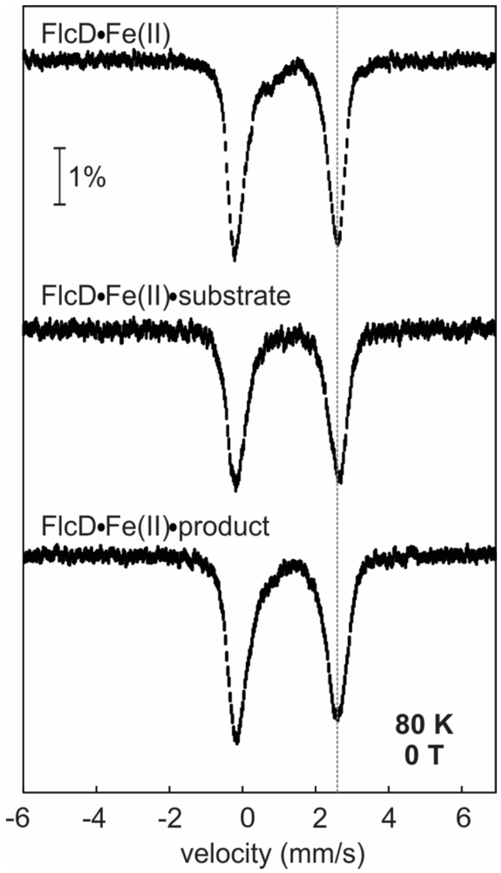


**Figure S29. The 80 K Mössbauer spectra of anoxic FlcD•Fe(II), FlcD•Fe(II)•substrate, and FlcD•Fe(II)•product.** Spectra of an anoxic solution of FlcD (1.7 mM) to which 1 molar eq of Fe(II) was added: as is (top), after addition of 3 eq of substrate, **2** (middle), after addition of 3 eq of product, **3** (bottom). The experimental spectra are shown as black vertical bars and were acquired in the absence of an external magnetic field.

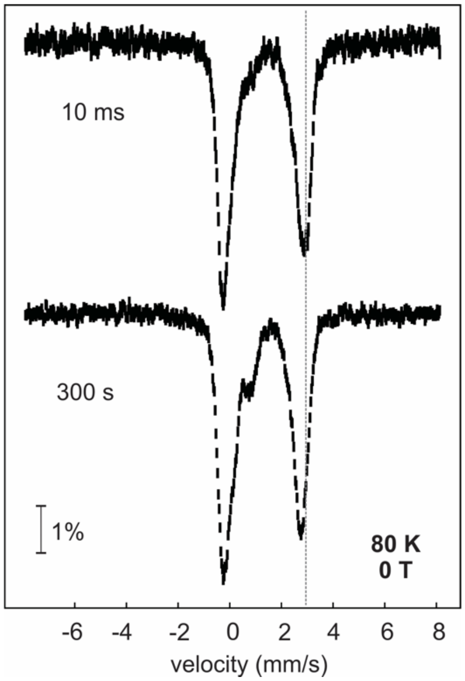


**Figure S30. The 80 K Mössbauer spectra of the FlcD reaction.** Samples of 2 molar eq of Fe(II) and 3 eq of substrate, **2**, were added to an anoxic solution of FlcD (1.7 mM), which was subsequently reacted with $O_2$ -saturated buffer (1.8 mM) in a 1:1 ratio and freeze-quenched at 10 ms (top) and 5 min (bottom), respectively, in liquid isopentane. The experimental spectra are shown as black vertical bars and were acquired in the absence of an external magnetic field.

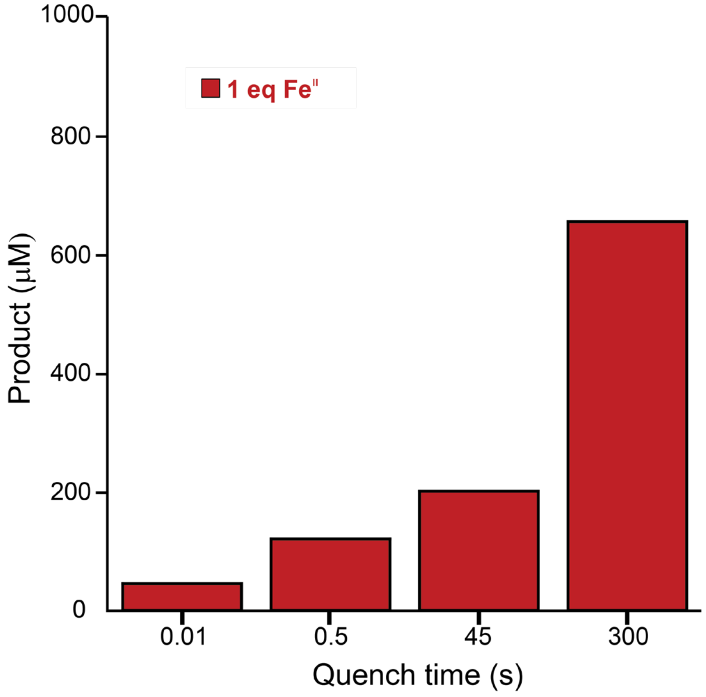


**Figure S31. Time-dependent product formation in reactions of the Y270F variant reconstituted with one molar eq of Fe(II).** Single turnover conditions are identical to those of the WT (main text Figure 3). Reactions were quenched by addition of an equal volume of 3.5% $H_2SO_4$. Product formation was quantified using integrated UV peak area from LC analysis. Electrospray HRMS analysis under positive ion mode was used to verify the retention time of the FlcD product.

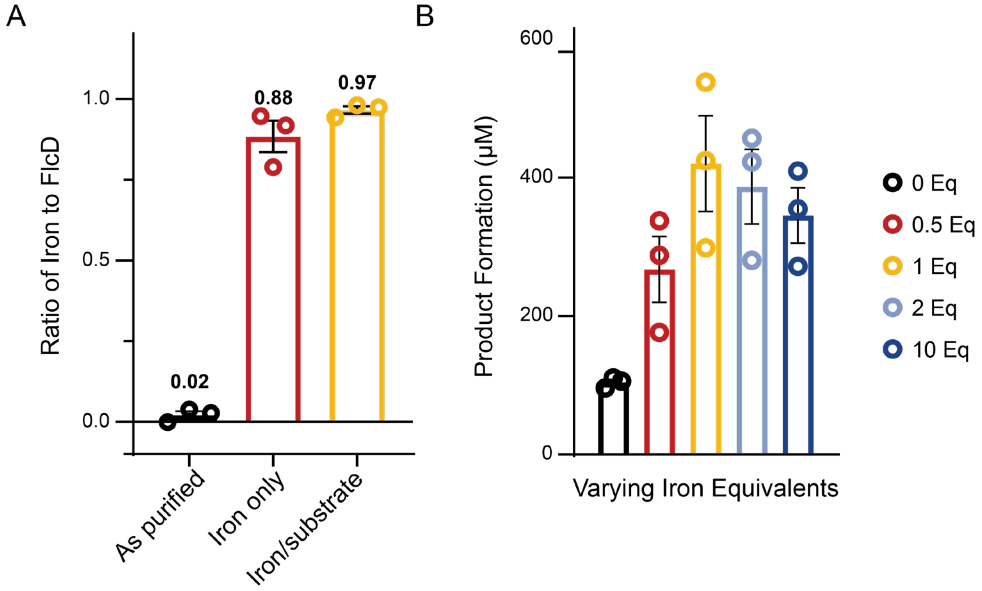


**Figure S32. Iron content analysis of FlcD supports the presence of a mononuclear iron cofactor.** A) Ratio of iron to FlcD determined by a ferrozine assay. $FlcD_{tagless}$ was reconstituted in the anaerobic chamber with 13.3 equivalents of Fe(II) in the absence or presence of 13.3 equivalents of substrate, **2**. Excess iron was removed by buffer exchange. Very little iron was detected in $FlcD_{tagless}$ as purified. $FlcD_{tagless}$ reconstituted with Fe(II) only or both Fe(II) and substrate contains an average of 0.88 and 0.97 equivalents of iron, respectively. The experiment was performed in triplicate with the average (numbers above the bars) and SEM (error bars) shown. B) Activity of $FlcD_{tagless}$ with varying equivalents of Fe(II) added. $FlcD_{tagless}$ was incubated with **2**, sodium ascorbate, and 0, 0.5, 1, 2, or 10 equivalents of Fe(II). The reaction was quenched at 2 min using 1.75% TFA. Product formation was calculated based on UV peak areas at 250 nm on LC and product retention time was confirmed by electrospray ionization HRMS under positive ion mode. Reactions were performed in triplicate with the average and SEM shown. SEM bars for 0 eq are hidden behind the black circles.

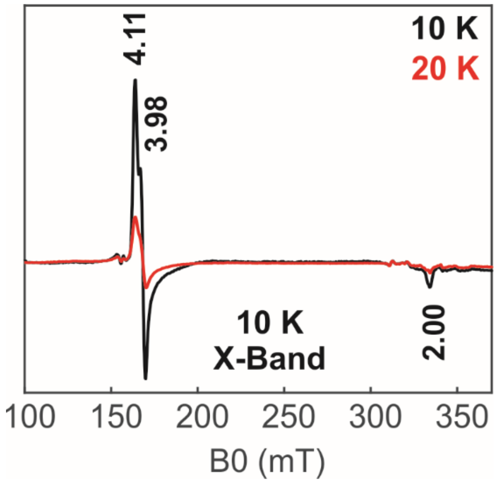


**Figure S33. CW EPR spectra of anoxic FlcD.** An anoxic solution of FlcD (1.7 mM) was reconstituted with 1 molar eq of Fe(II) and 3 eq of substrate, **2**, and then reacted with a solution saturated with $O_2$ (1.8 mM) and rapidly frozen after 5 min in liquid isopentane. The inverse temperature dependence of the $S$ = 3/2 signal is consistent with a positive sign for the axial zero-field splitting parameter D and the proposal that the transition originates from the ±1/2 manifold. The anaerobic control was subtracted from these spectra to remove any background signals due to adventitious high-spin Fe(III) at g ~ 4.3 and of a small amount of Mn(II) that copurifies with the protein and interferes with the g = 2 signal. Experimental conditions: T = 10 K (black trace), T = 20 K (red trace), microwave frequency 9.36 GHz, microwave power 2 mW, and modulation amplitude 1 mT.

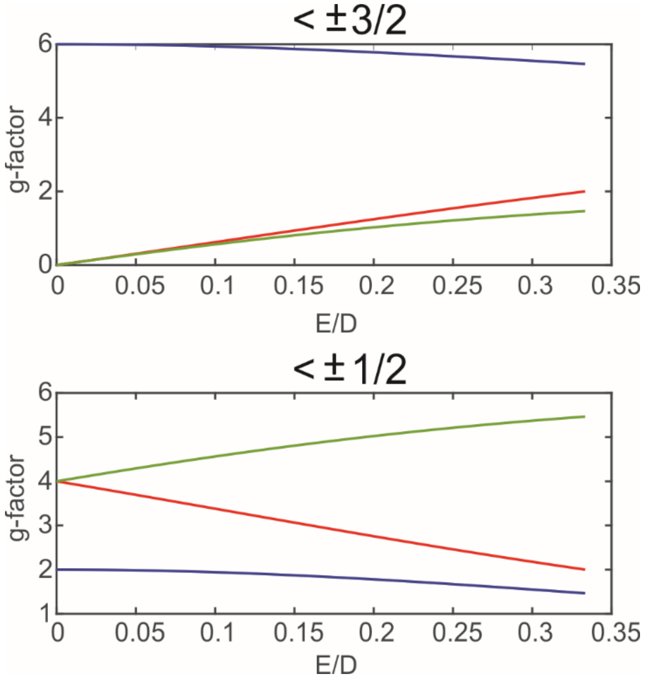


**Figure S34. Rhombogram for an S = 3/2 system with a positive axial zero-field splitting value D.** The rhombogram describes various electronic g-factor groupings for a given rhombicity E/D. The red, green, and blue lines correspond to the x (green), y (red), and z (blue) g-factor components in the three coordinate axes.

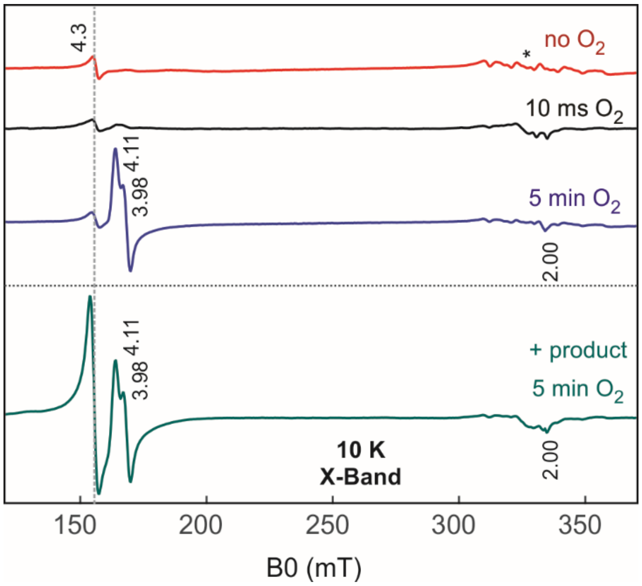


**Figure S35. CW EPR spectra of FlcD•Fe(II)•substrate and FlcD•Fe(II)•product reacted in the absence and presence of $O_2$.** The CW EPR spectrum of the anoxic FlcD•Fe(II)•substrate sample is shown in red, of the 10 ms reaction with an $O_2$-saturated buffer is shown in black, of the 5 min reaction with $O_2$-saturated buffer is shown in blue, and the spectrum of the FlcD•Fe(II)•product complex reacted with $O_2$-saturated buffer for 5 min is shown in dark green. The asterisk denotes the hexa-line signal corresponding to Mn(II) that copurifies with the apo form of FlcD. Reactant concentrations prior to 1:1 mixing: [FlcD] = 1.7 mM, [Fe(II)] = 1.7 mM, [substrate/product] = 5.1 mM, [$O_2$] =1.8 mM. Experimental conditions: T = 10 K, microwave frequency 9.36 GHz, microwave power 2 mW, and modulation amplitude 1 mT.

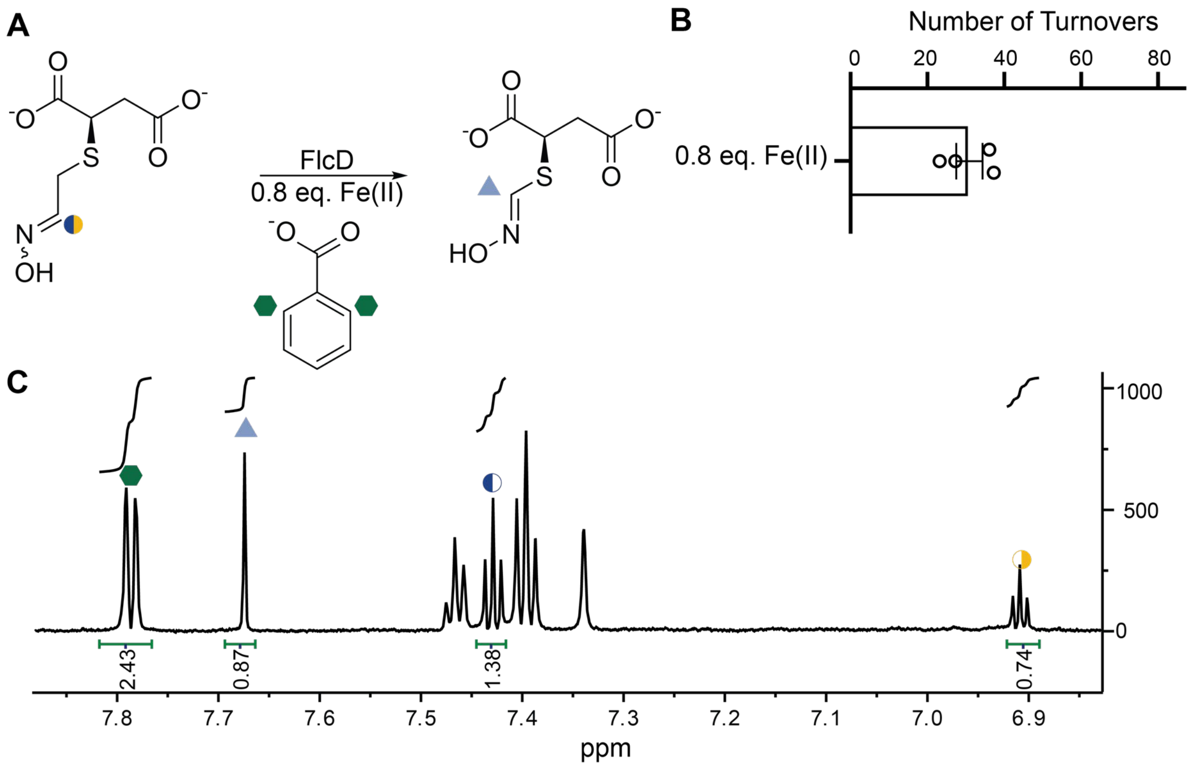


**Figure S36. $^{1}$H NMR quantification of product formation in FlcD reactions containing 0.8 Fe(II) equivalents.** A) Reaction scheme of FlcD reconstituted with 0.8 equivalents of Fe(II) and reacted for 3 h. Benzoic acid was added to the reaction as an internal standard immediately before analysis using an 850 MHz spectrometer. Integrated protons are labeled with shapes. FlcD substrate is a mixture of *E* and *Z* isomers of the oxime. B) Number of turnovers in the FlcD reaction. C) Representative NMR spectrum showing integrated peaks for the reaction. Labeled peaks correspond to protons shown in (A).

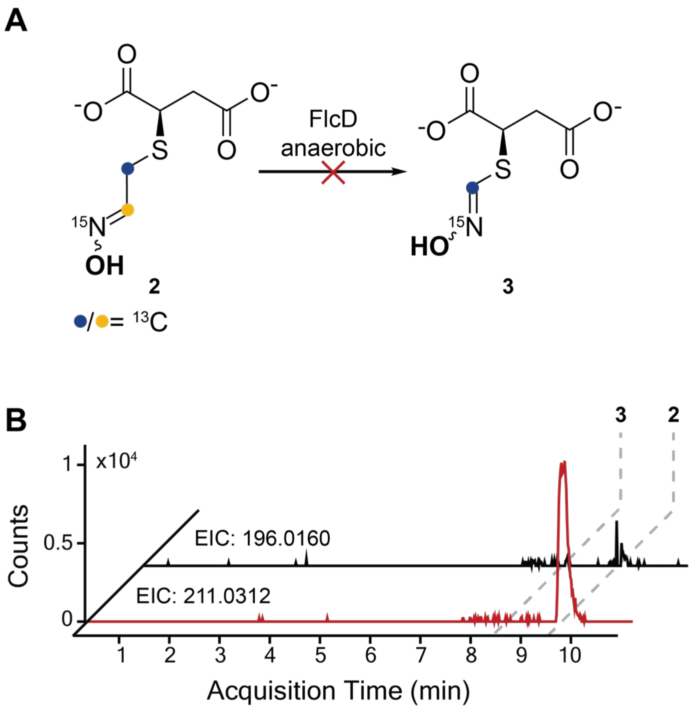


**Figure S37. The FlcD reaction requires $O_2$.** A) Scheme of FlcD-catalyzed reaction, which converts [$^{13}C_2$, $^{15}N$]-**2** to [$^{13}C$, $^{15}N$]-**3** ($^{13}C$ carbons labeled with blue/yellow dots). B) LC-HRMS analysis of the FlcD reaction under anaerobic environment. EICs of the substrate [$^{13}C_2$, $^{15}N$]-**2** (red, $[M + H]^+$ = 211.0312) and product (black, $[M + H]^+$ = 196.0160) are shown with the expected retention times highlighted (dotted gray line). No product (**3**) was formed in the absence of $O_2$. Spikes in the spectra are background noise. LC-HRMS analysis was performed using electrospray ionization under positive ion mode.

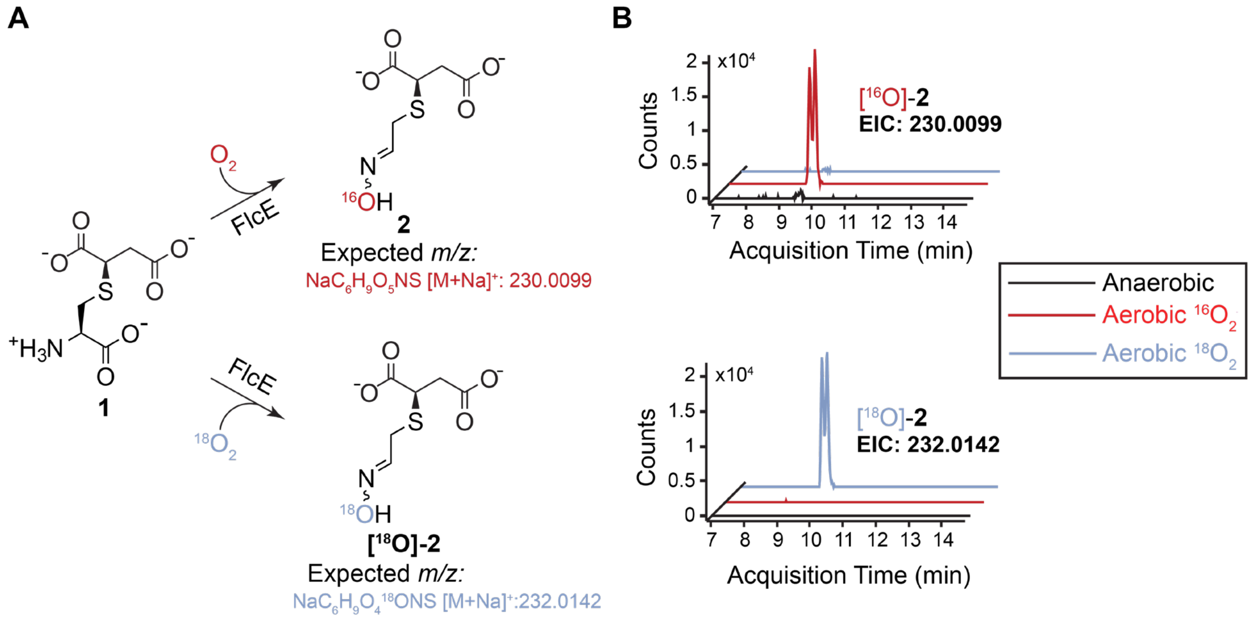


**Figure S38. FlcE incorporates one oxygen from $O_2$ into the oxime hydroxyl.** A) Scheme of the FlcE reaction. A FlcE reaction was incubated in either a $^{16}O_2$ (top, red) or an $^{18}O_2$ (bottom, blue) environment, or no $O_2$. B) EICs of the FlcE product in the $^{16}O_2$ reaction (red), $^{18}O_2$ reaction (blue), or anaerobic control (black) from LC-HRMS analysis. Top, EIC of $^{16}$O-**2**; bottom, EIC of $^{18}$O-**2**. LC-HRMS analysis was performed using electrospray ionization under positive ion mode.

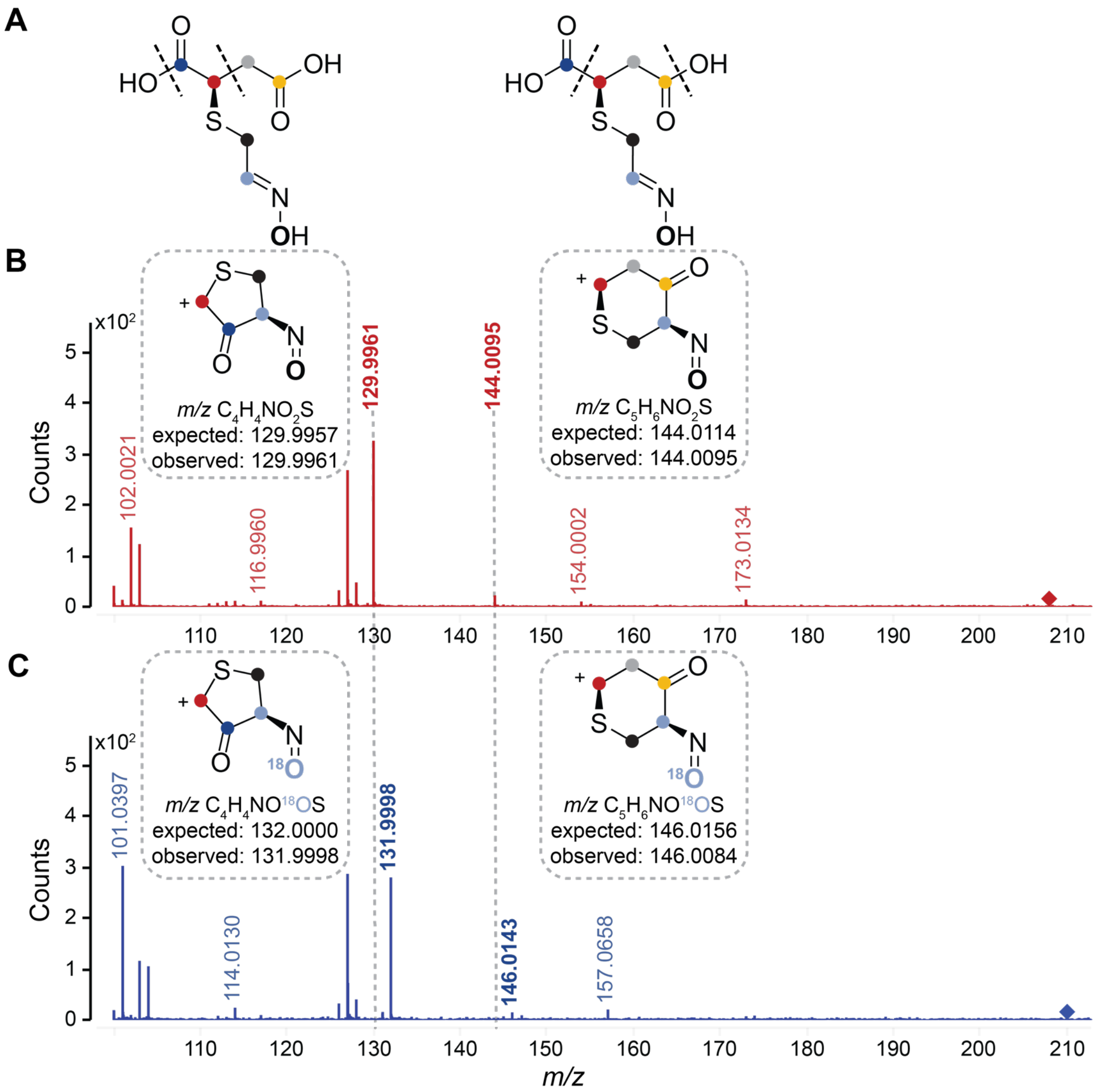


**Figure S39. FlcE incorporates oxygen from $O_2$ into the oxime hydroxyl.** A) Proposed ionization of FlcE product with each carbon labeled with a different color circle. Black dotted lines indicate fragments lost. Tandem mass spectra of the product of the FlcE reaction in a $^{16}O_2$ (B) or $^{18}O_2$ (C) environment from LC-HRMS analysis. Dotted gray lines are used to indicate fragments of interest in (B) with a shift of +2 Da in (C). Dotted gray boxes show the proposed structure, formula, and *m/z* of the ions of interest. The fragments are consistent with an oxygen atom from $O_2$ being incorporated into the oxime. LC-HRMS analysis was performed using electrospray ionization under positive ion mode.

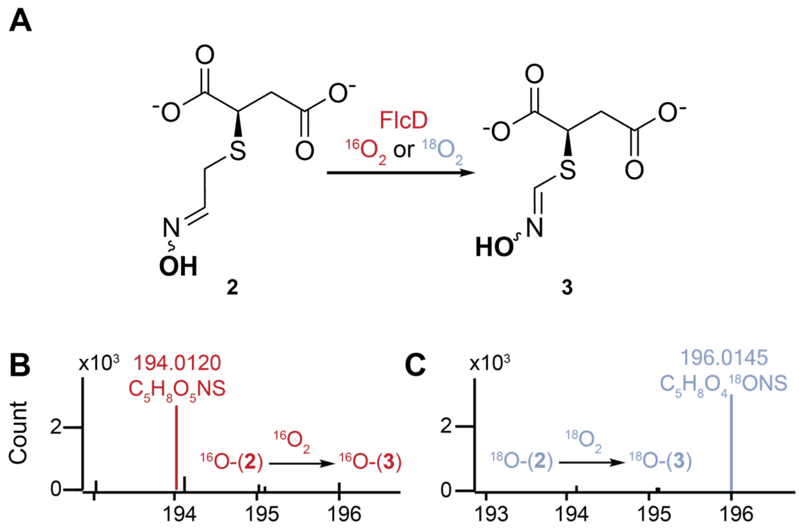


**Figure S40. The FlcD-catalyzed reaction retains the oxime oxygen.** A) Scheme of the FlcD-catalyzed reaction in a $^{16}O_2$ or $^{18}O_2$ environment. The bolded hydroxyl indicates the oxygen being tracked. B) Mass spectra of **3** after reacting $^{16}$O-**2** with FlcD in a $^{16}O_2$ environment. Only $^{16}$O-**3** was observed. C) Mass spectra of **3** after reacting $^{18}$O-**2** with FlcD in an $^{18}O_2$ environment. Only $^{18}$O-**3** was observed. LC-HRMS analysis was performed using electrospray ionization under positive ion mode.

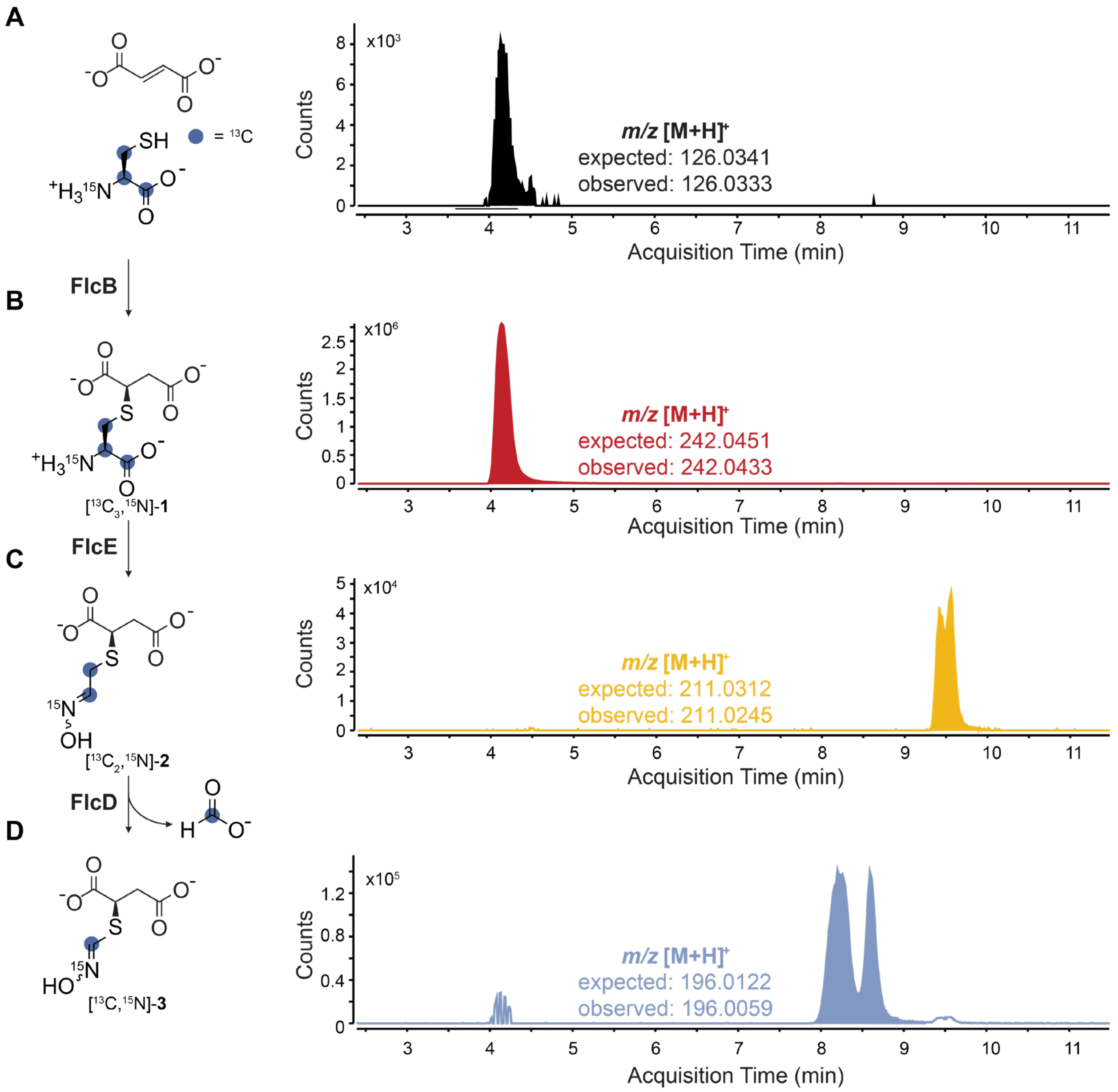


**Figure S41. Biosynthesis of [$^{13}$C, $^{15}$N]-labeled metabolites in the fluopsin C pathway.** A) The starting materials fumarate and [$^{13}C_3$, $^{15}$N]-L-cysteine with $^{13}$C shown in blue dots (left). EIC of [$^{13}C_3$, $^{15}$N]-L-cysteine (right). B) FlcB catalyzes the conversion of [$^{13}C_3$, $^{15}$N]-L-cysteine and fumarate to [$^{13}C_3$, $^{15}$N]-**1** (left). EIC of [$^{13}C_3$, $^{15}$N]-**1** (right). C) FlcE catalyzes the conversion of [$^{13}C_3$, $^{15}$N]-**1** to [$^{13}C_2$, $^{15}$N]-**2** (left). EIC of [$^{13}C_2$, $^{15}$N]-**2** (right). D) FlcD catalyzes the conversion of [$^{13}C_2$, $^{15}$N]-**2** to [$^{13}$C, $^{15}$N]-**3** (left) and [$^{13}$C]-formic acid. EIC of [$^{13}$C, $^{15}$N]-**3** (right). LC-HRMS analysis was performed using electrospray ionization under positive ion mode.

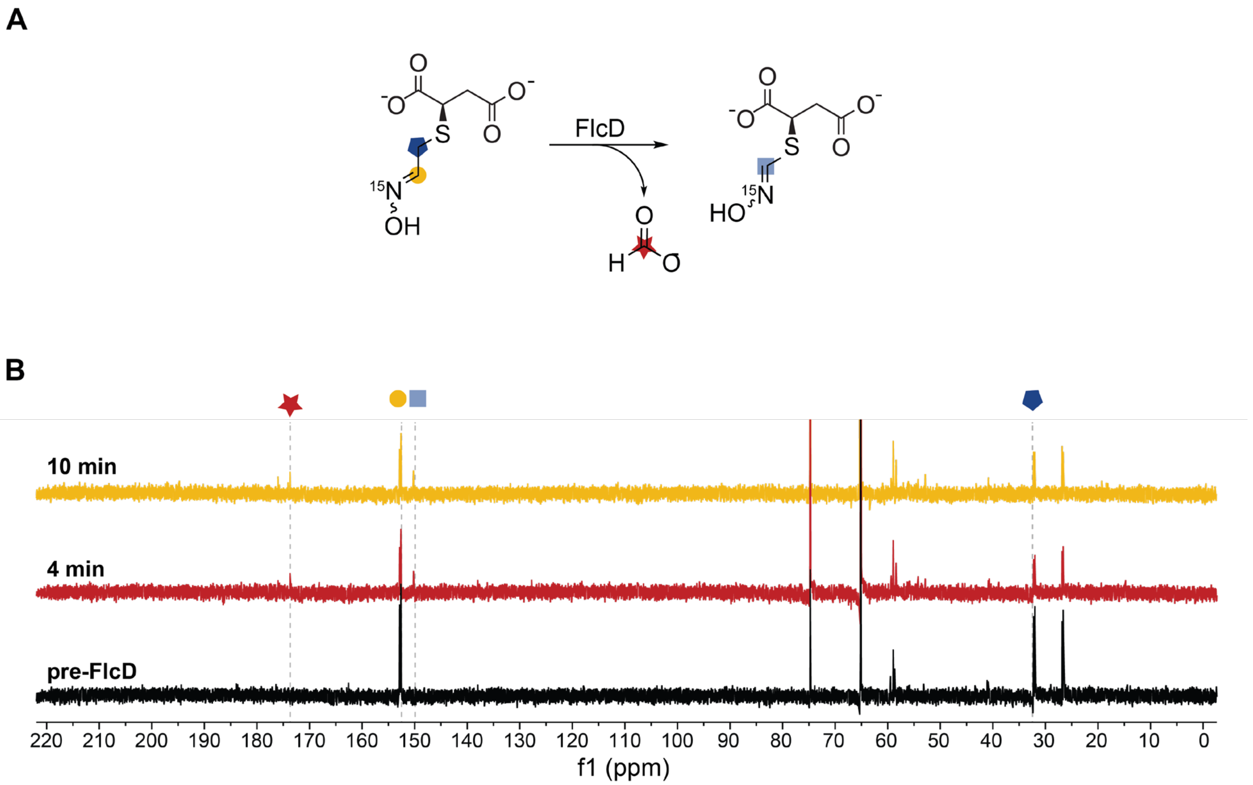


**Figure S42. $^{13}$C NMR verifies production of formic acid in the reaction of FlcD.** A) FlcD reaction scheme, [$^{13}C_2$,$^{15}$N]-**2** ($^{13}$C-circle and pentagon) is converted to formic acid ($^{13}$C-star) and [$^{13}$C,$^{15}$N]-**3** ($^{13}$C-square). B) $^{13}$C NMR spectra of a reaction containing [$^{13}C_2$, $^{15}$N]-**2** before addition of FlcD (black) or after incubation with FlcD for 4 min (red) and 10 min (yellow). Peaks labeled with various shapes correspond to the carbons shown in the reaction scheme (A).

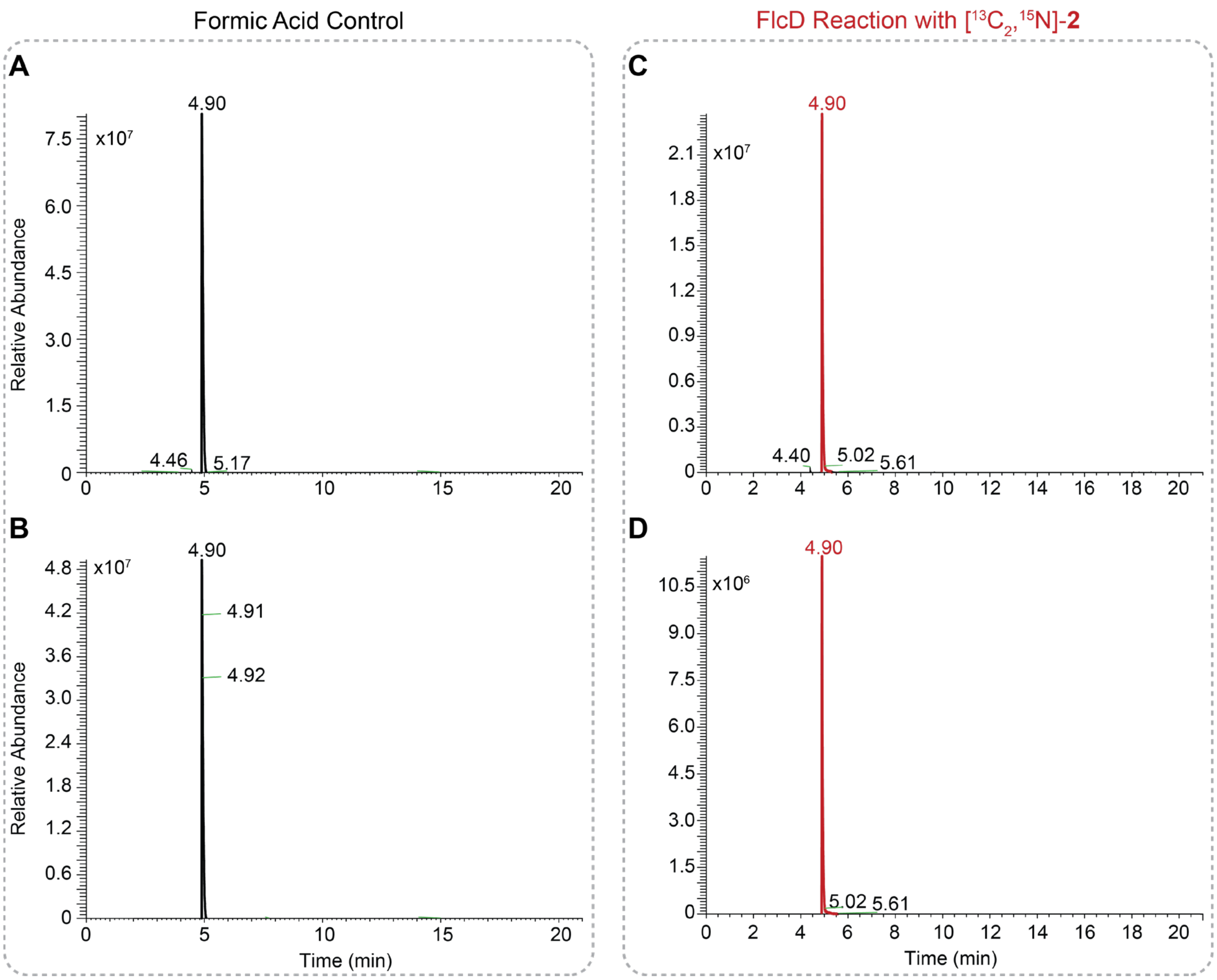


**Figure S43. GC-HRMS analysis showed that formic acid produced by the reaction of FlcD possesses the same retention time as a formic acid standard.** A–B) EICs of A) $CHO_2$ $[M]^+$ = 44.99711 and B) $CH_2O_2$ $[M]^{+\bullet}$ = 46.00493 of a 3.2 mM formic acid control. C–D) EICs of C) $^{13}CHO_2$ $[M]^+$ = 46.00046 and D) $^{13}CH_2O_2$ $[M]^{+\bullet}$ = 47.00826 produced in a reaction where [$^{13}C_2$, $^{15}N$]-**2** was incubated with FlcD in air. EICs were generated using a 1.5 ppm error window. The $^{13}C$-formic acid produced in the FlcD reaction elutes at 4.90 min, which is identical to the formic acid control. GC-HRMS analysis was performed using electron ionization under positive ion mode.

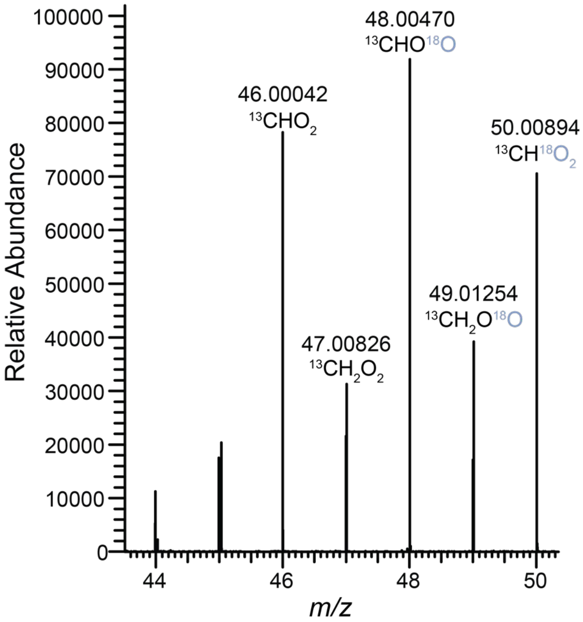


**Figure S44. Representative GC mass spectrum of formic acid produced in a reaction where [$^{13}C_2$, $^{15}N$]-2 was incubated with FlcD in an $^{18}O_2$ environment for 10 min.** Labeled peaks are within 1.5 ppm error of the calculated *m*/*z*. GC-HRMS analysis was performed using electron ionization under positive ion mode.

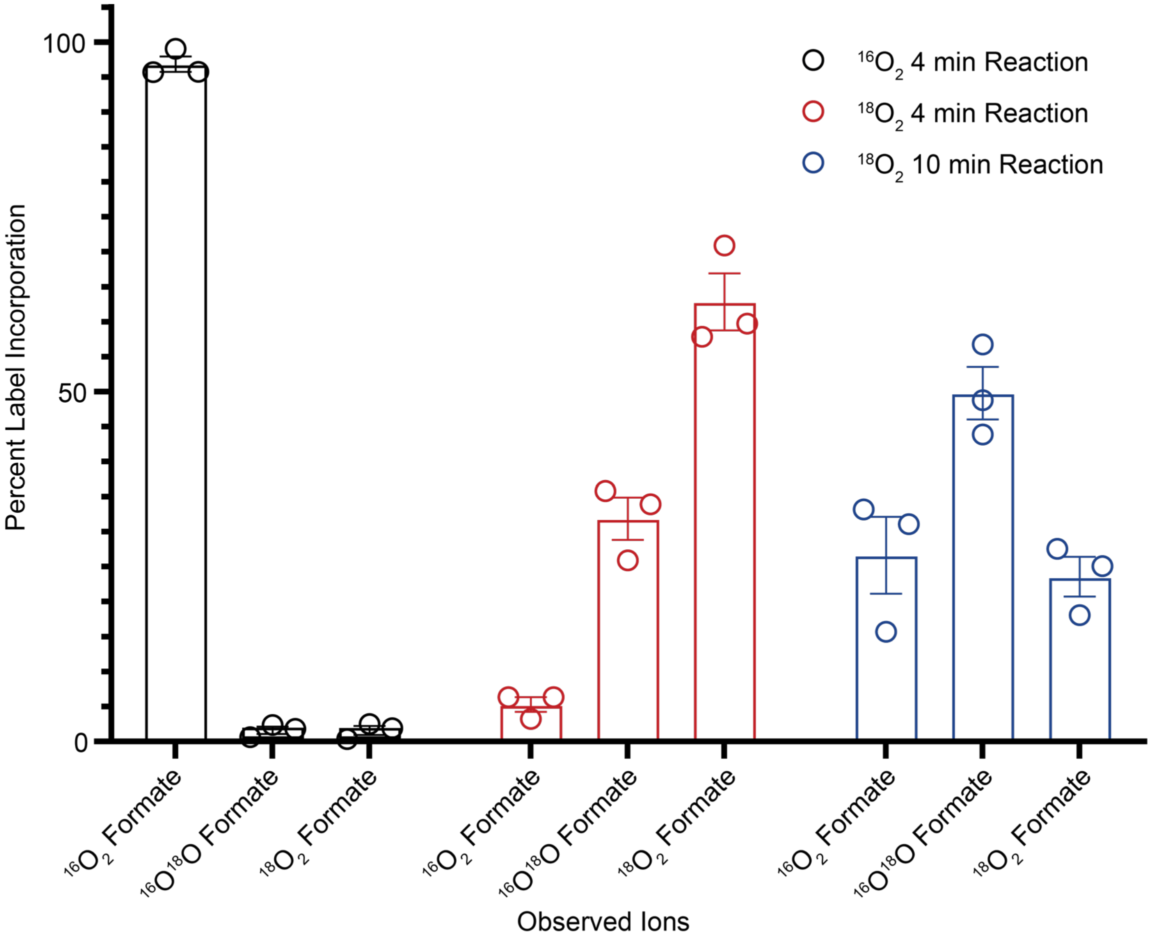


**Figure S45. GC-HRMS quantification of labeled formic acid.** EIC peaks of ions corresponding to $^{16}O_2$-formic acid, $^{16}O^{18}O$-formic acid, or $^{18}O_2$-formic acid were separately added and the fraction of each formic acid species is plotted for a 4 min $^{16}O_2$ reaction (black), 4 min $^{18}O_2$ reaction (red), or 10 min $^{18}O_2$ reaction (blue). The average of triplicate experiments and standard error of the mean (SEM) are shown. GC-HRMS analysis was performed using electron ionization under positive ion mode.

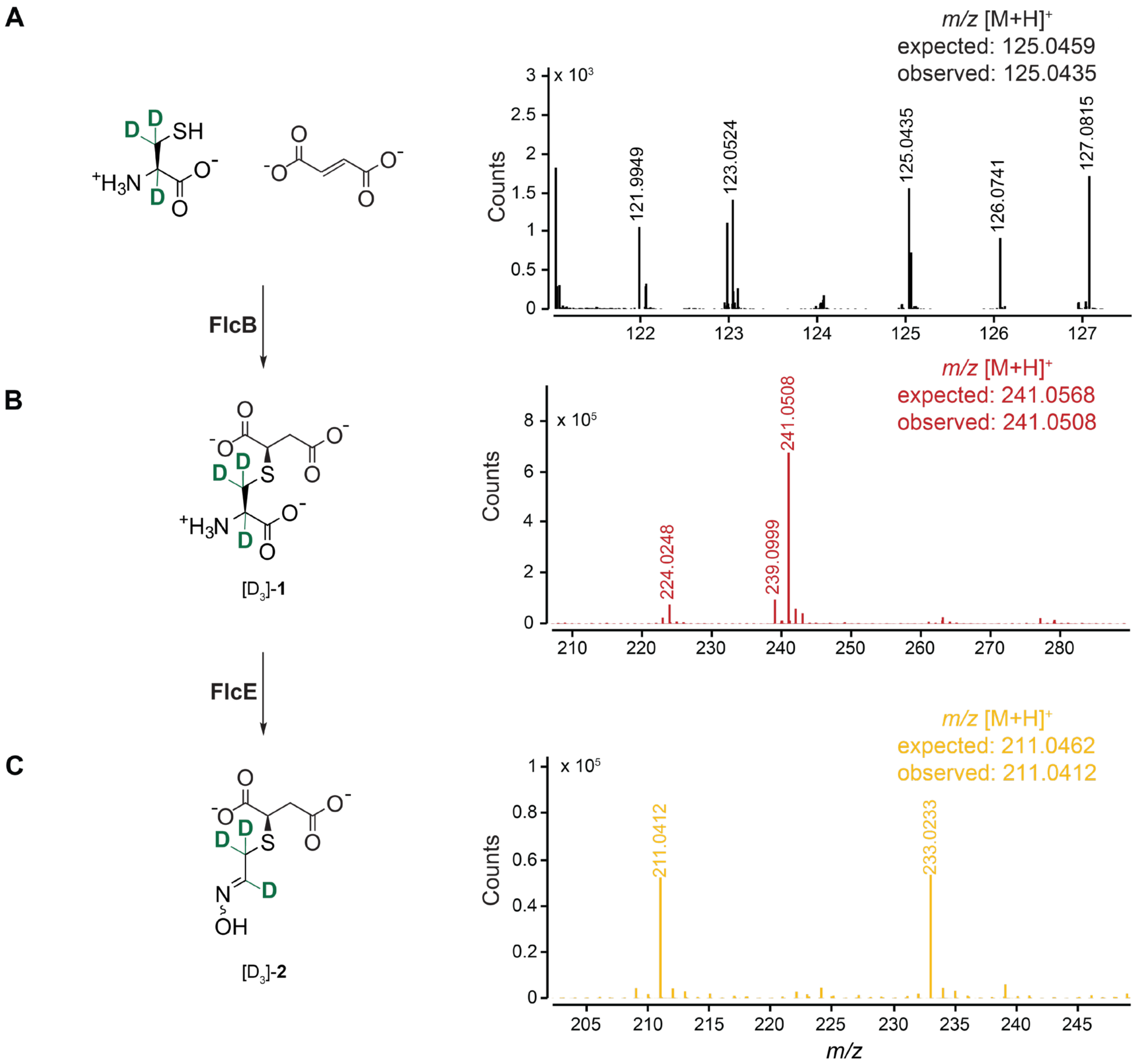


**Figure S46. Biosynthesis of [$D_3$]-labeled metabolites in the fluopsin C pathway.** A) Reactants fumarate and $D_3$-L-cysteine (left). Extracted mass spectrum (MS) of [$D_3$]-L-cysteine (right). B) FlcB reaction with [$D_3$]-L-cysteine and fumarate yields [$D_3$]-**1** (left). MS of [$D_3$]-**1** (right). C) FlcE reaction with [$D_3$]-**1** yields [$D_3$]-**2** (left). MS of [$D_3$]-**2** (right). LC-HRMS analysis was performed using electrospray ionization under positive ion mode.

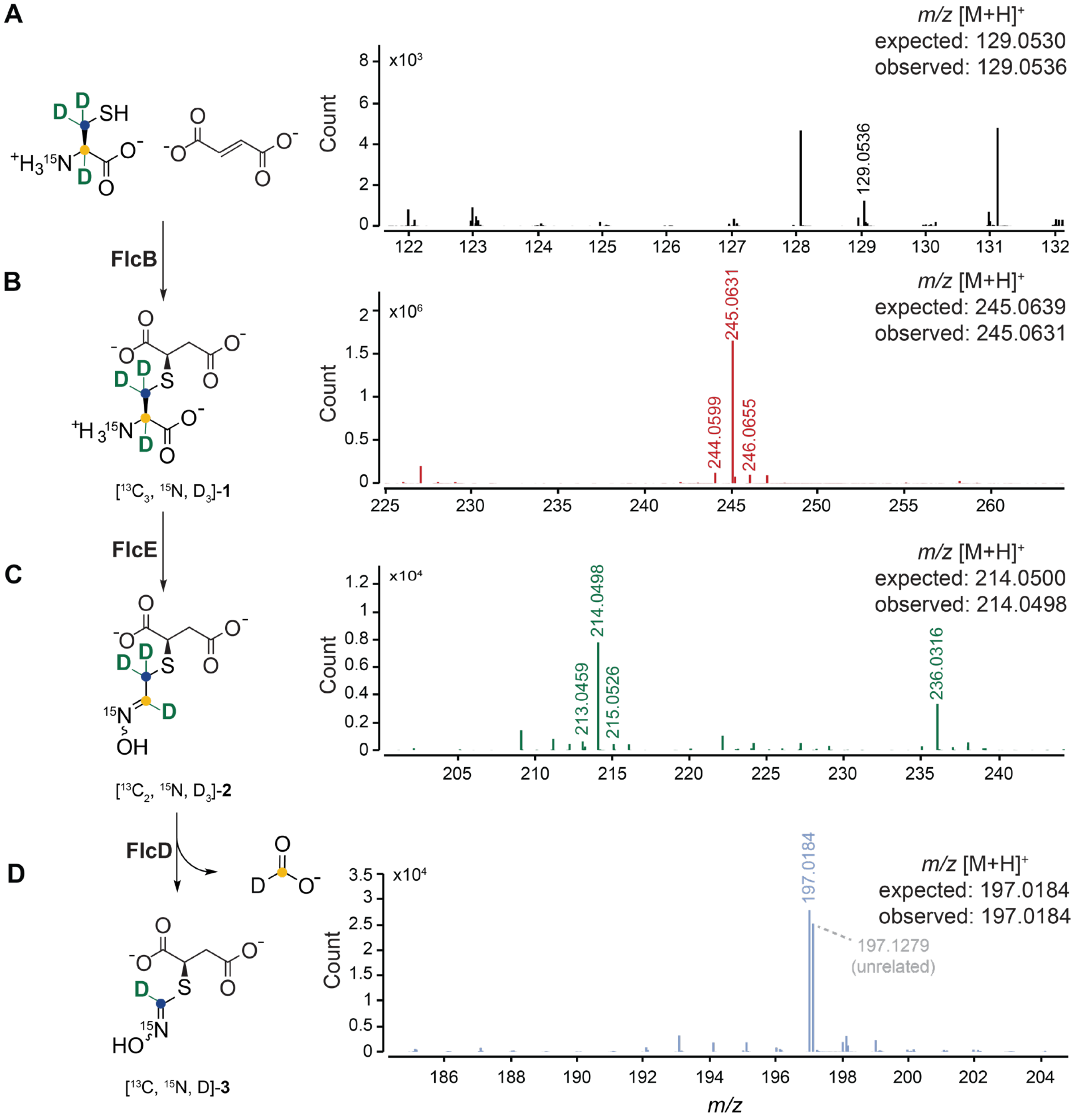


**Figure S47. Biosynthesis of [$^{13}C$, $^{15}N$, $D_3$]-labeled metabolites in the fluopsin C pathway.** A) Reactants fumarate and [$^{13}C_3$, $^{15}N$, $D_3$]-L-cysteine (left). Extracted mass spectrum (MS) of [$^{13}C_3$, $^{15}N$, $D_3$]-L-cysteine (right). B) FlcB reaction with [$^{13}C_3$, $^{15}N$, $D_3$]-L-cysteine and fumarate yields [$^{13}C_3$, $^{15}N$, $D_3$]-**1** (left). MS of [$^{13}C_3$, $^{15}N$, $D_3$]-**1** (right). C) FlcE reaction with [$^{13}C_3$, $^{15}N$, $D_3$]-**1** yields [$^{13}C_2$, $^{15}N$, $D_3$]-**2** (left). MS of [$^{13}C_2$, $^{15}N$, $D_3$]-**2** (right). D) FlcD reaction with [$^{13}C_2$, $^{15}N$, $D_3$]-**2** yields [$^{13}C$, $^{15}N$, D]-**3** (left) and [D]-formic acid. MS of [$^{13}C$, $^{15}N$, D]-**3** shows one deuterium is retained in the FlcD reaction (right). LC-HRMS analysis was performed using electrospray ionization under positive ion mode.

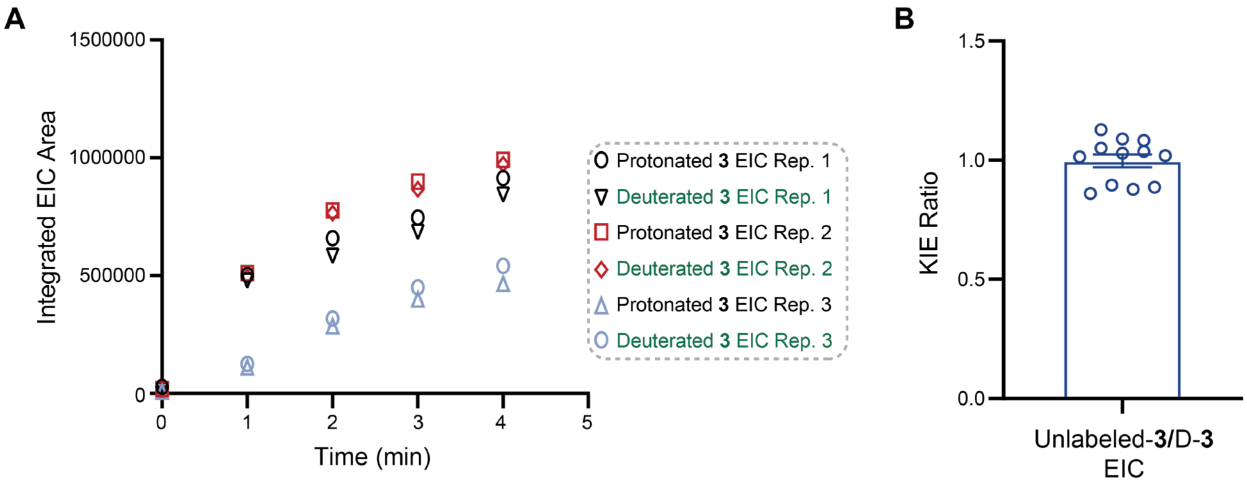


**Figure S48. The reaction of FlcD did not exhibit an apparent kinetic isotope effect (KIE) toward the dideuterated substrate, $D_2$-2.** A) FlcD reaction incubated with equal molar concentrations of unlabled-2 and $D_2$-2 and analyzed by LC-HRMS for product formation at each timepoint (1–4 min). The EIC areas are shown for triplicate experiments. B) Average KIE ratio with SEM shown for all three replicates. The KIE ratio was obtained by dividing the amount of unlabeled 3 by that of [D]-3 formed at each time point (blue circles). LC-HRMS analysis was performed using electrospray ionization under positive ion mode.

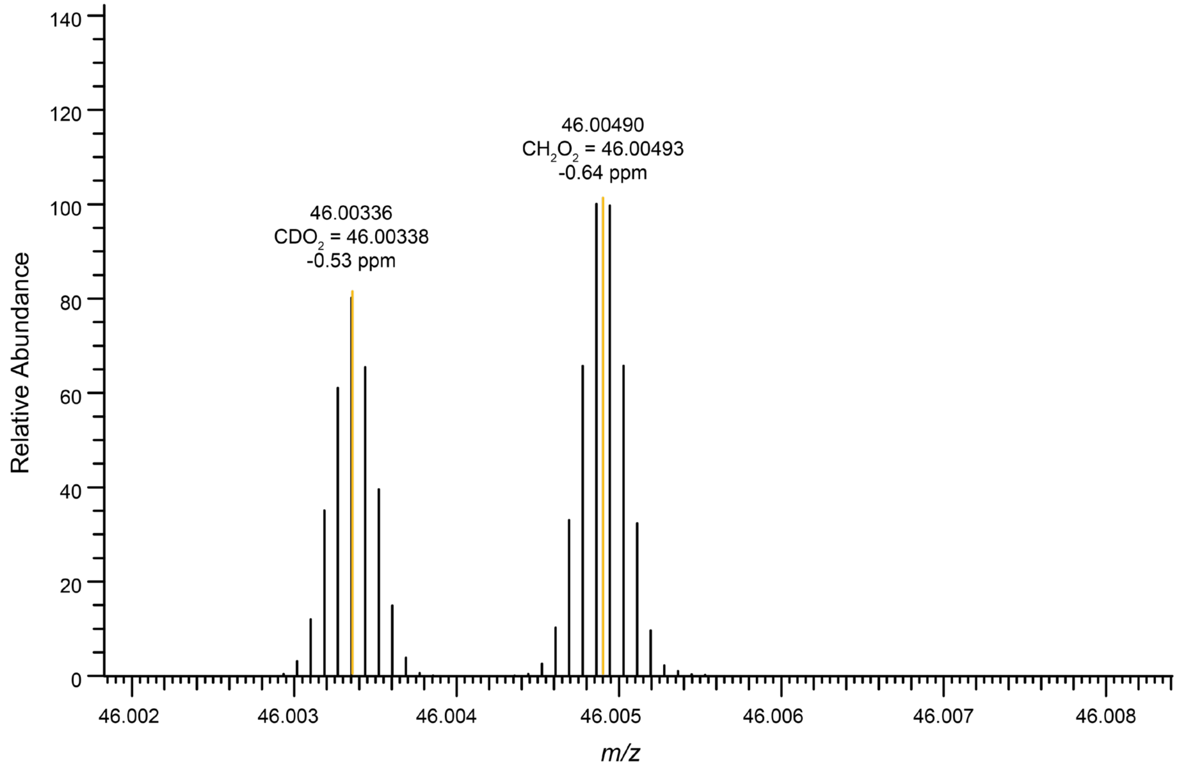


**Figure S49. GC-HRMS can differentiate ions with high accuracy.** Example of a zoomed-in mass spectrum of formic acid produced in a 10-min FIcD reaction. Isotope peaks, such as $CDO_2$ and $CH_2O_2$, are resolved with less than 1 ppm error. GC-HRMS analysis was performed using electron ionization under positive ion mode.

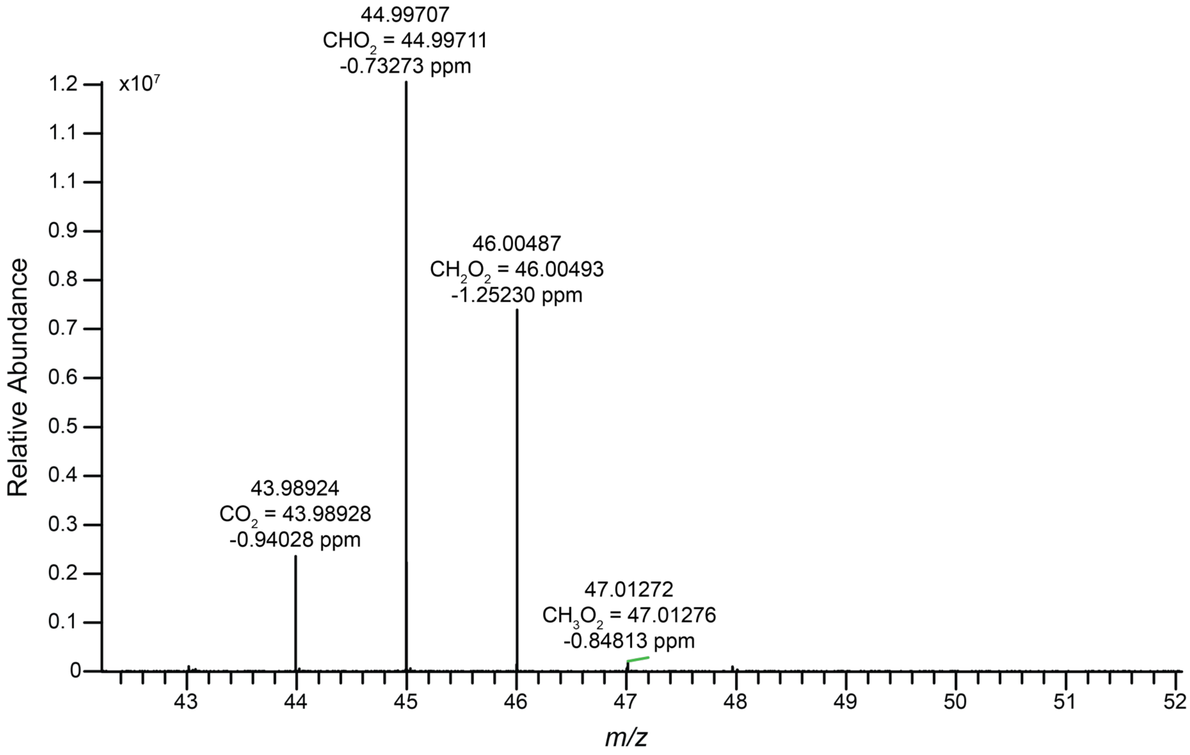


**Figure S50. Mass spectrum of a formic acid standard.** Example of the ionization patterns observed for formic acid. GC-HRMS analysis was performed using electron ionization under positive ion mode.

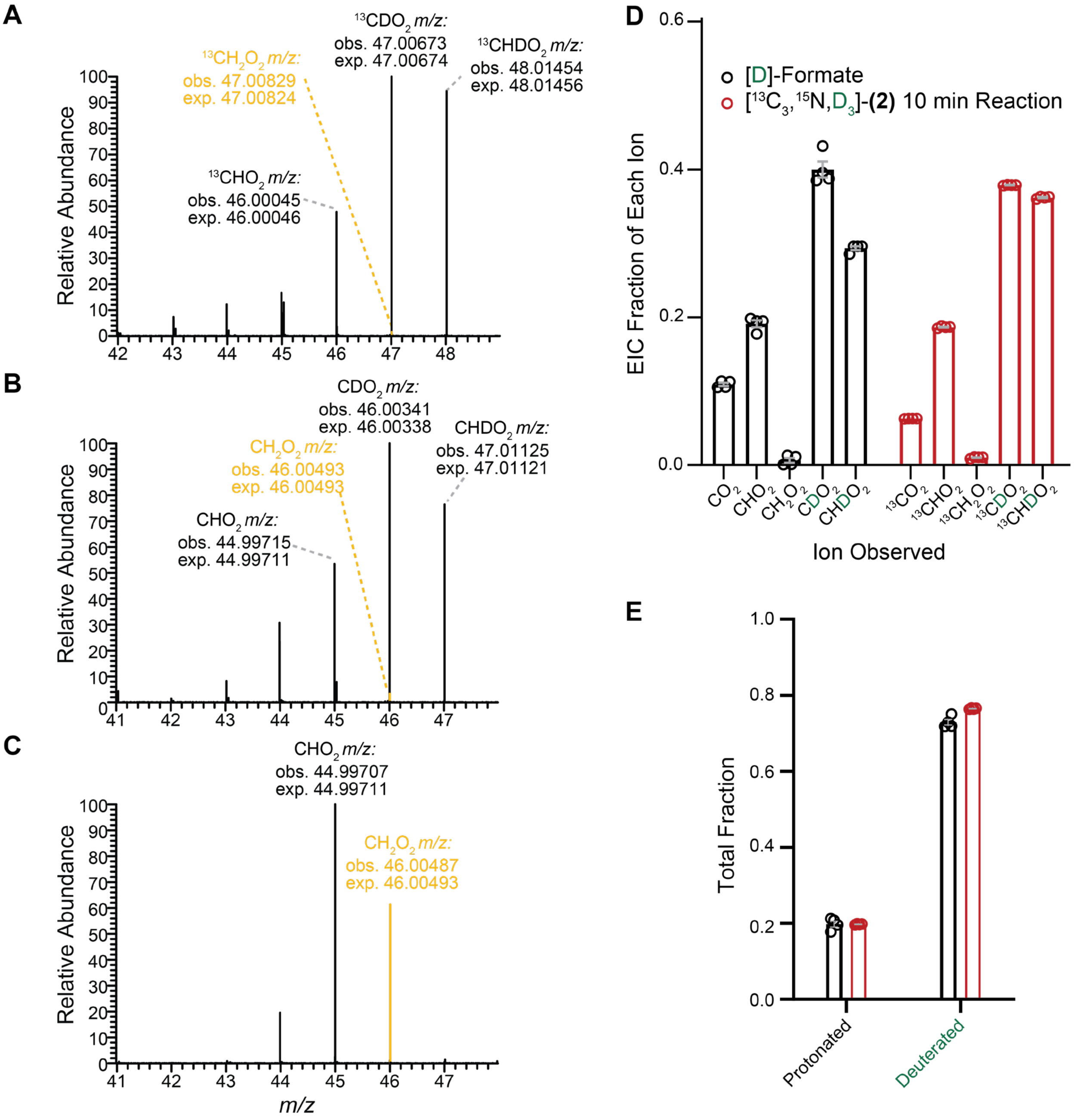


**Figure S51. GC-HRMS analysis of formate produced in the FlcD reaction containing [$^{13}C_2$, $^{15}N$, $D_3$]-2.** Mass spectrum of A) formate produced in a 10-min FlcD reaction incubated with [$^{13}C_2$, $^{15}N$, $D_3$]-**2,** of B) a 3.2 mM formic acid standard, and of C) a 3.2 mM sodium D-formate standard. Peaks are labeled with the proposed ion formula, observed (obs.) *m/z*, and the expected (exp.) *m/z*. D) Fraction EIC for each ion observed for a D-formate standard (black) and formate produced in the FlcD reaction containing [$^{13}C_2$, $^{15}N$, $D_3$]-**2** (red). E) Summation of the protonated species ($CHO_2$, $CH_2O_2$ or $^{13}CHO_2$, $^{13}CH_2O_2$) and deuterated species ($CDO_2$, $CHDO_2$ or $^{13}CDO_2$, $^{13}CHDO_2$) shown in (D). The average of four replicates (circles) and SEM are shown in D) and E). GC-HRMS analysis was performed using electron ionization under positive ion mode.

| Protein | NCBI accession ID |
|---|---|
| UndA | WJN79797.1 |
| BesC | WP_014151496.1 |
| CADD | AAC68213.1 |
| SznF | QBA82042.1 |
| FlcE | AAG06907.1 |
| FlcD | AAG06906.1 |

**Figure S52. NCBI accession IDs of HDOs used in bioinformatic analysis.**

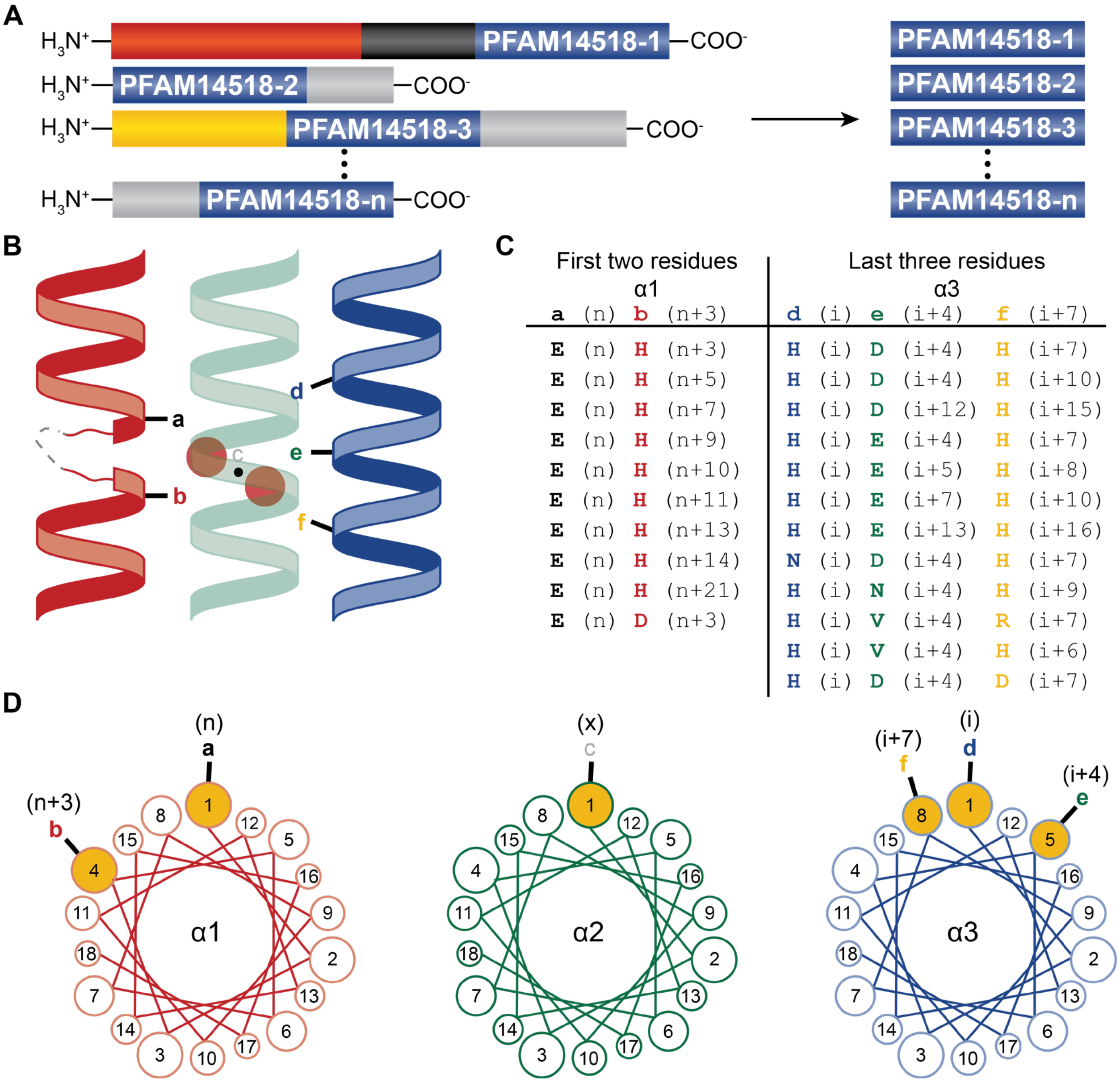


| First two residues α1 | | Last three residues α3 | | |
|---|---|---|---|---|
| a (n) | b (n+3) | d (i) | e (i+4) | f (i+7) |
| E (n) | H (n+3) | H (i) | D (i+4) | H (i+7) |
| E (n) | H (n+5) | H (i) | D (i+4) | H (i+10) |
| E (n) | H (n+7) | H (i) | D (i+12) | H (i+15) |
| E (n) | H (n+9) | H (i) | E (i+4) | H (i+7) |
| E (n) | H (n+10) | H (i) | E (i+5) | H (i+8) |
| E (n) | H (n+11) | H (i) | E (i+7) | H (i+10) |
| E (n) | H (n+13) | H (i) | E (i+13) | H (i+16) |
| E (n) | H (n+14) | N (i) | D (i+4) | H (i+7) |
| E (n) | H (n+21) | H (i) | N (i+4) | H (i+9) |
| E (n) | D (n+3) | H (i) | V (i+4) | R (i+7) |
| | | H (i) | V (i+4) | H (i+6) |
| | | H (i) | D (i+4) | D (i+7) |

**Figure S53. Bioinformatic approach to identify potential iron-binding motif of uncharacterized HDOs.** A) HDO sequences containing multiple domains or regions were trimmed to contain only the HDO domain (blue). B) Three core α-helices involved in iron-binding with residues 1 and 2 on α1 (red), residue 3 on α2 (gray), and residues 4–6 on α3 (blue). C) List of potential locations of iron-binding residues on the α1 and α3 helices. (i/n) is the location of the first residue on α1 or α3 with the remaining residues found at varying distances. For example, an (n+3) label indicates that residue is identified three residues after the first residue on α1. D) Helical representation of the location of potential iron-binding residues in HDOs. The single residue on α2 was not analyzed. Loop insertions are found in some helices based on AlphaFold2 predictions, thus the spacing of residues on the same face of the helix do not follow the canonical residue spacing of α-helices.

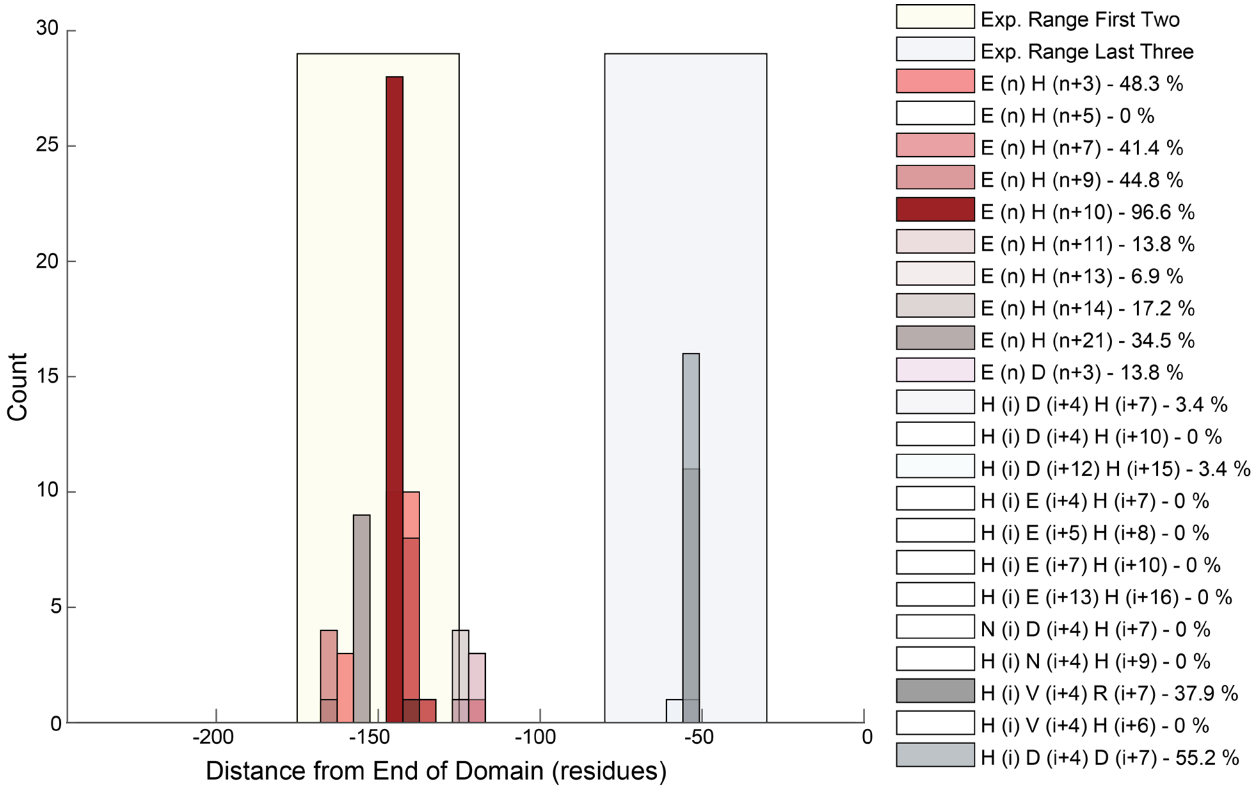


**Figure S54. Histogram analysis of potential iron-binding motif for SSN group 15 containing FlcD and FlcE.** Potential motifs are listed on the right. Color intensity indicates the frequency of each motif within the group. The first two residues are expected to fall within the yellow shaded box (left) and the last three residues are expected to fall within the blue shaded box (right). This analysis predicts the iron-binding motif follows E (n) H (n+10) for the first two residues (96.6% frequency), and H (i) V (i+4) R (i+7) (37.9% frequency) or H (i) D (i+4) D (i+7) (55.2% frequency) for the last three residues, which corresponds to the motif found in FlcD and predicted for FlcE, respectively.

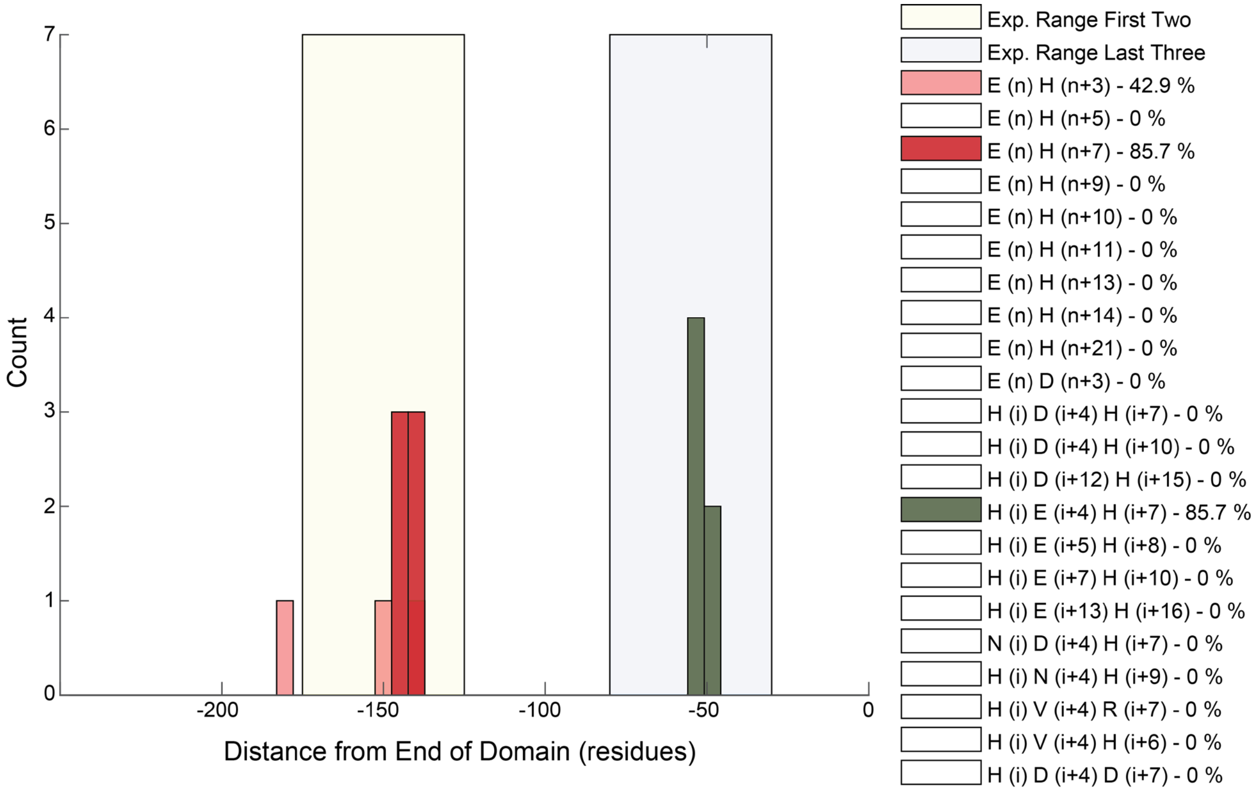


**Figure S55. Histogram analysis of potential iron-binding motif for SSN group 65 containing BesC.** Potential motifs are listed on the right. Color intensity indicates the frequency of each motif within the group. The first two residues are expected to fall within the yellow shaded box (left) and the last three residues are expected to fall within the blue shaded box (right). This analysis predicts the iron-binding motif follows E (n) H (n+7) (85.7% frequency) for the first two residues and H (i) E (i+4) H (i+7) (85.7% frequency) for the last three residues, which is consistent with the iron-binding motif found in BesC.

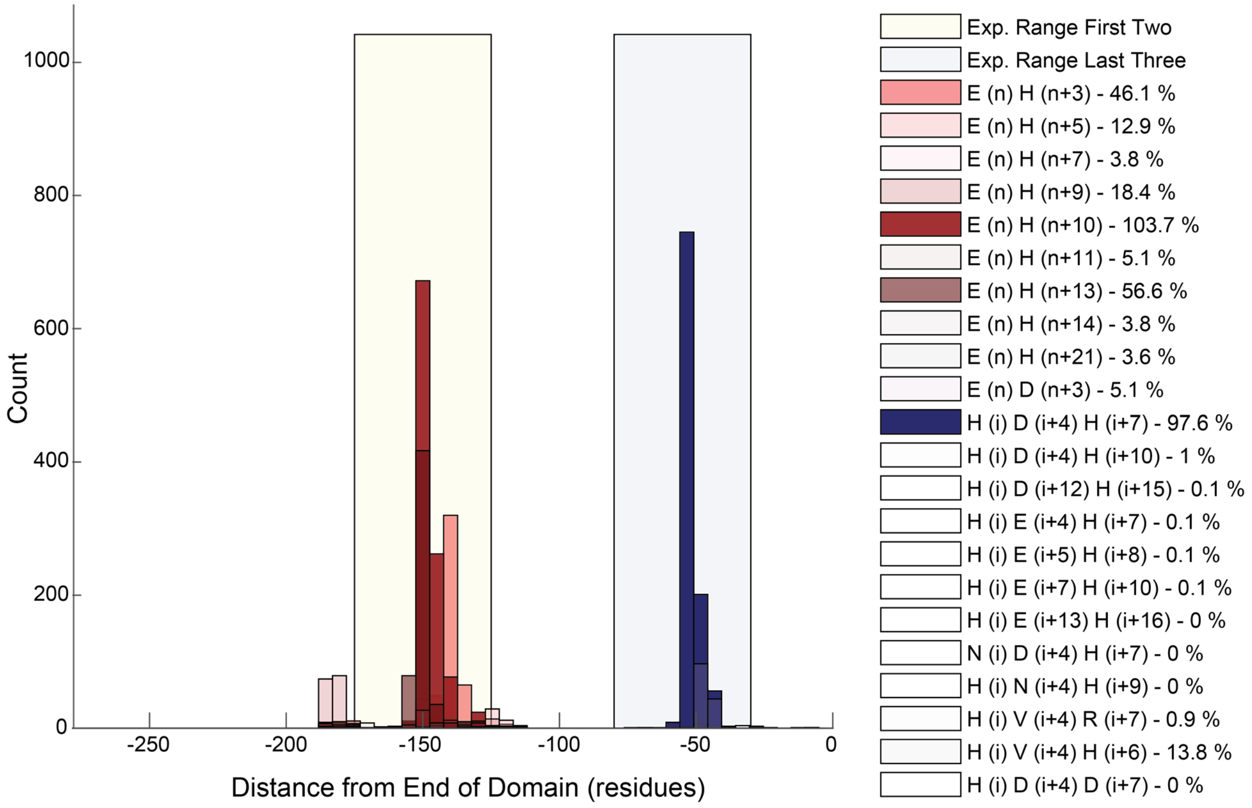


**Figure S56. Histogram analysis of potential iron-binding motif for SSN group 1 containing uncharacterized HDOs.** Potential motifs are listed on the right. Color intensity indicates the frequency of each motif within the group. The first two residues are expected to fall within the yellow shaded box (left) and the last three residues are expected to fall within the blue shaded box (right). This analysis predicts the iron-binding motif follows E (n) H(n+10) for the first two residues (103.7% frequency) and H (i) D (i+4) H (i+7) for the last three residues (97.6% frequency). The frequency of 103.7% suggests more than one E (n) H (n+10) motif in some sequences.

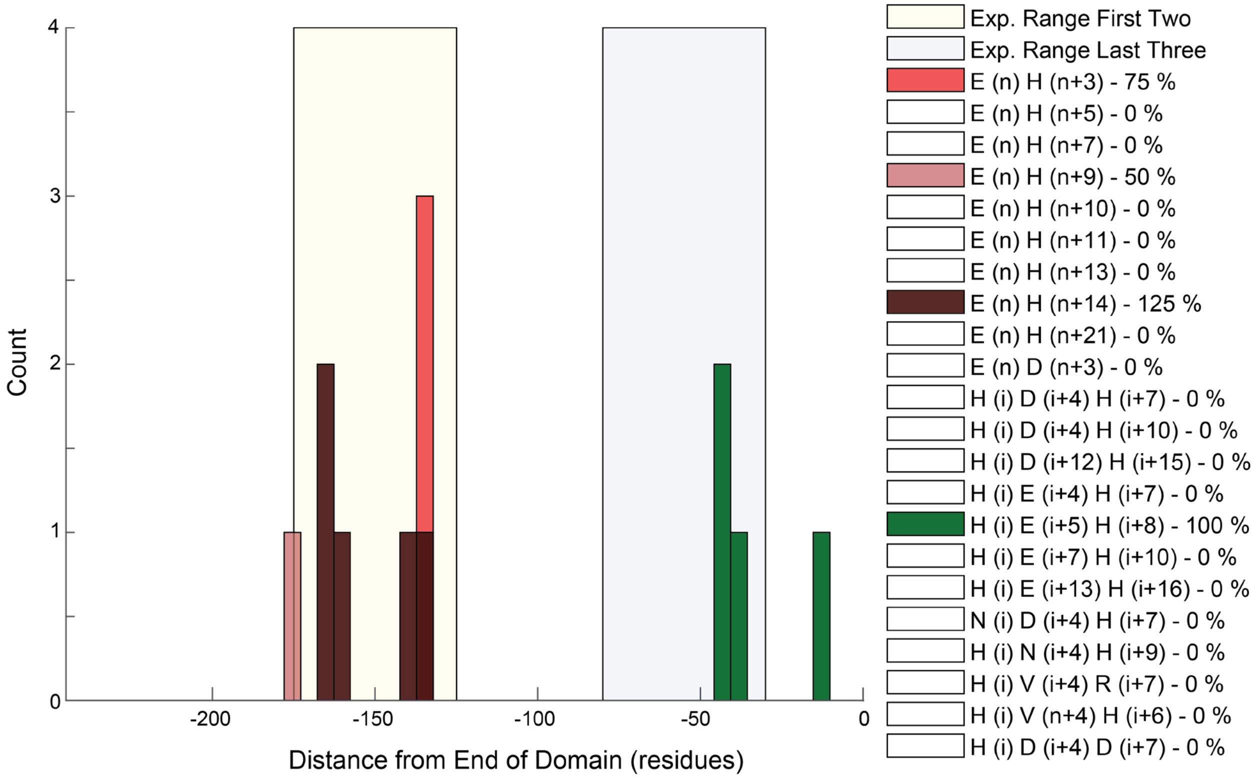


**Figure S57. Histogram analysis of potential iron-binding motif for SSN group 120 containing uncharacterized HDOs.** Potential motifs are listed on the right. Color intensity indicates the frequency of each motif within the group. The first two residues are expected to fall within the yellow shaded box (left) and the last three residues are expected to fall within the blue shaded box (right). This analysis predicts the iron-binding motif follows E (n) H (n+14) for the first two residues (125% frequency) and H (i) E (i+5) H (i+8) for the last three residues (100% frequency). The frequency of 125% suggests more than one E (n) H (n+14) motif in some sequences.

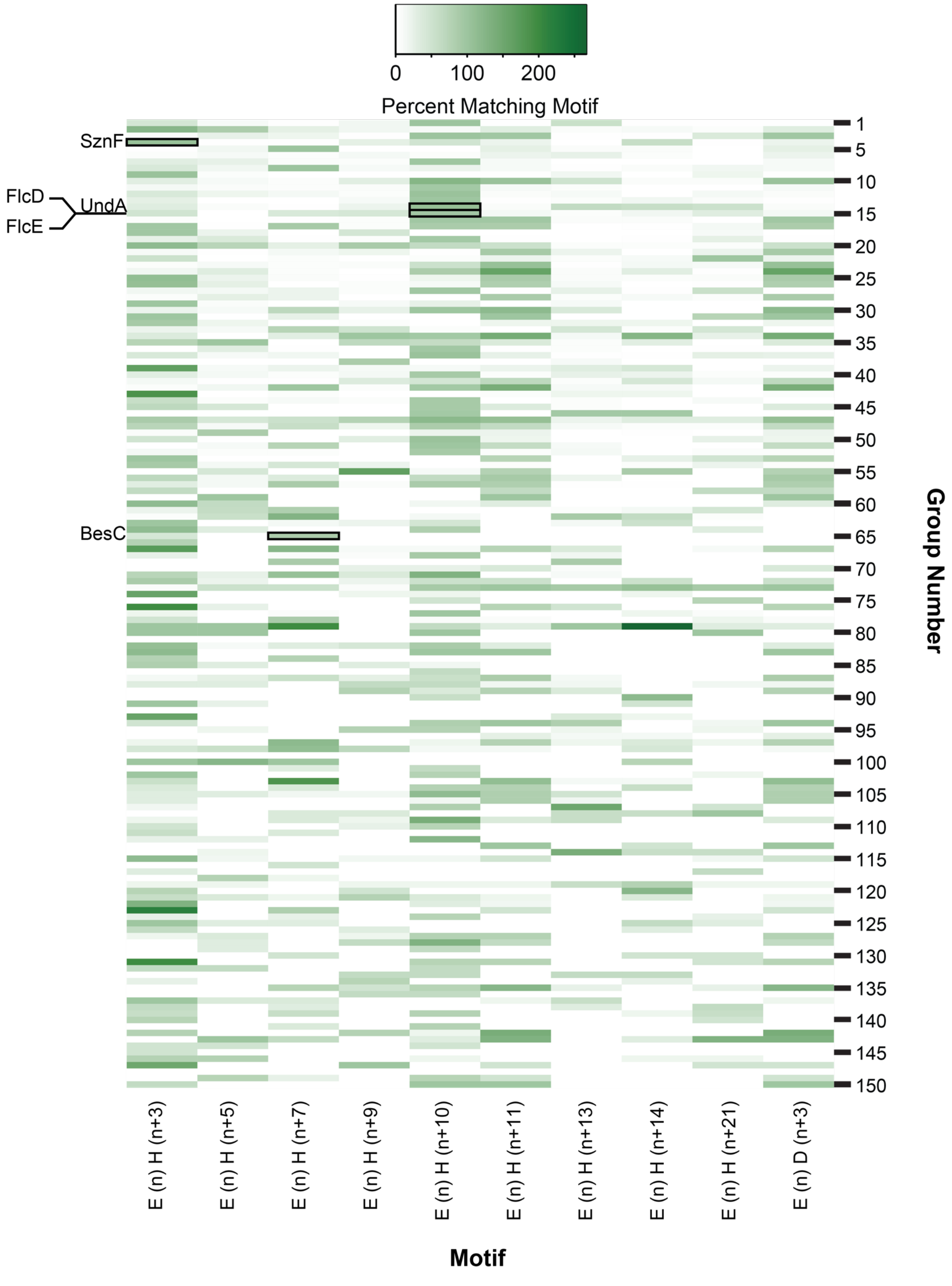


**Figure S58. Heatmap of the predicted first two iron-binding residues of HDOs.** For the first 150 groups of the SSN, the frequency of each motif within a group is plotted in a heatmap. A more intense green color indicates higher frequency. Characterized HDOs are boxed in their respective groups as references.

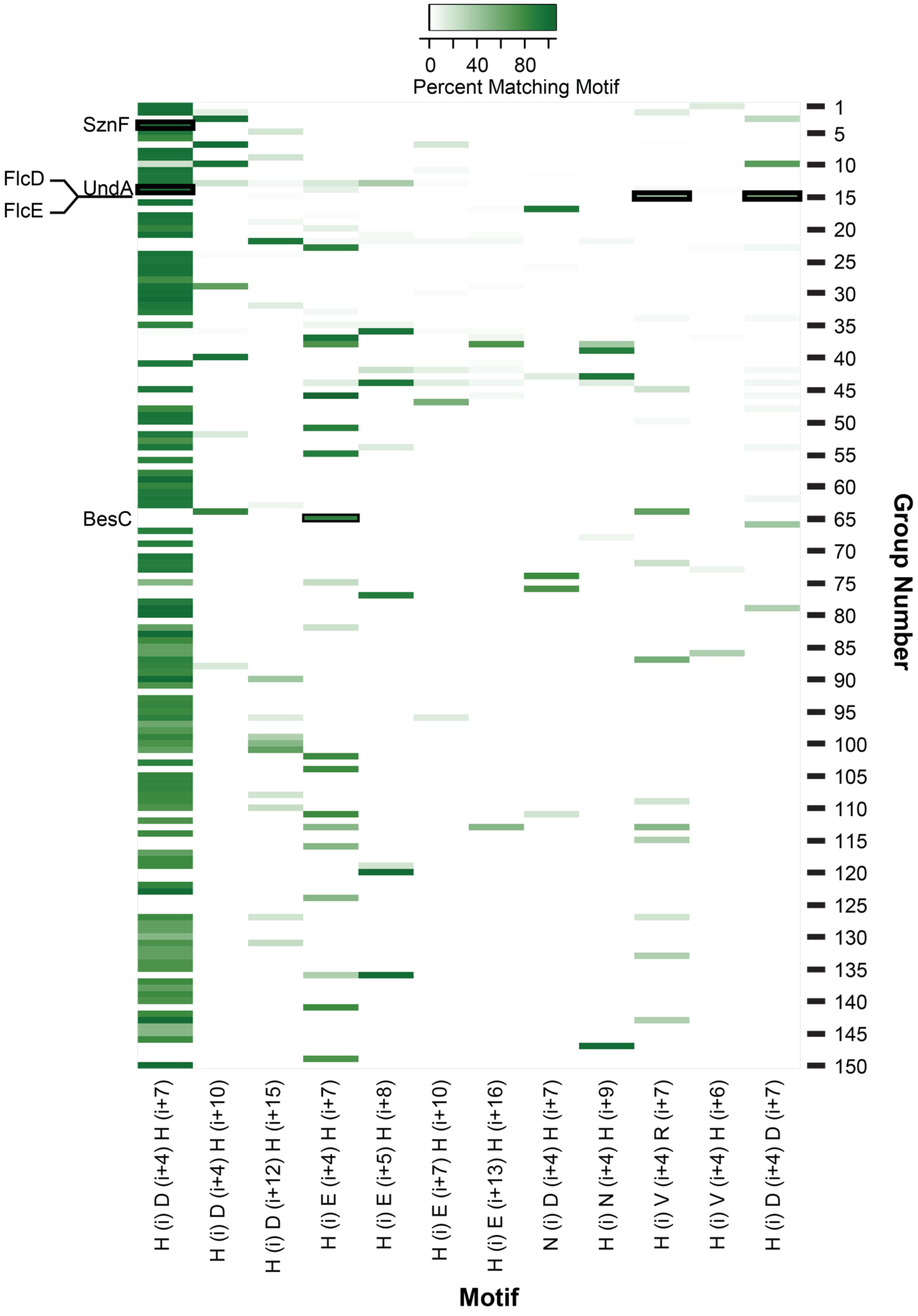


**Figure S59. Heatmap prediction of the last three iron-binding residues of HDOs.** For the first 150 groups of the SSN, the frequency of each motif within a group is plotted in a heatmap. A more intense green color indicates higher frequency. Characterized HDOs found within the SSN are boxed in their respective groups as references.

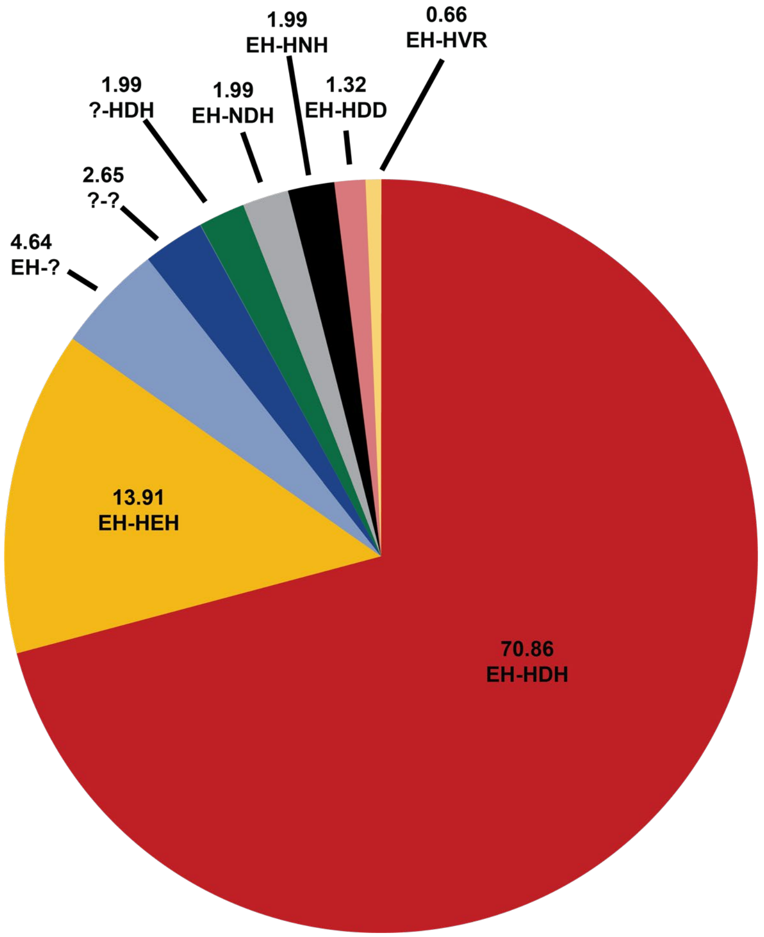


**Figure S60. Pie chart of the predicted first two and last three iron-binding residues for the first 150 groups of HDOs in the SSN.** The dash indicates that the third residue on α2 could not be predicted using the current algorithm. The number above each motif indicates the percentage of groups that were predicted to use this motif.

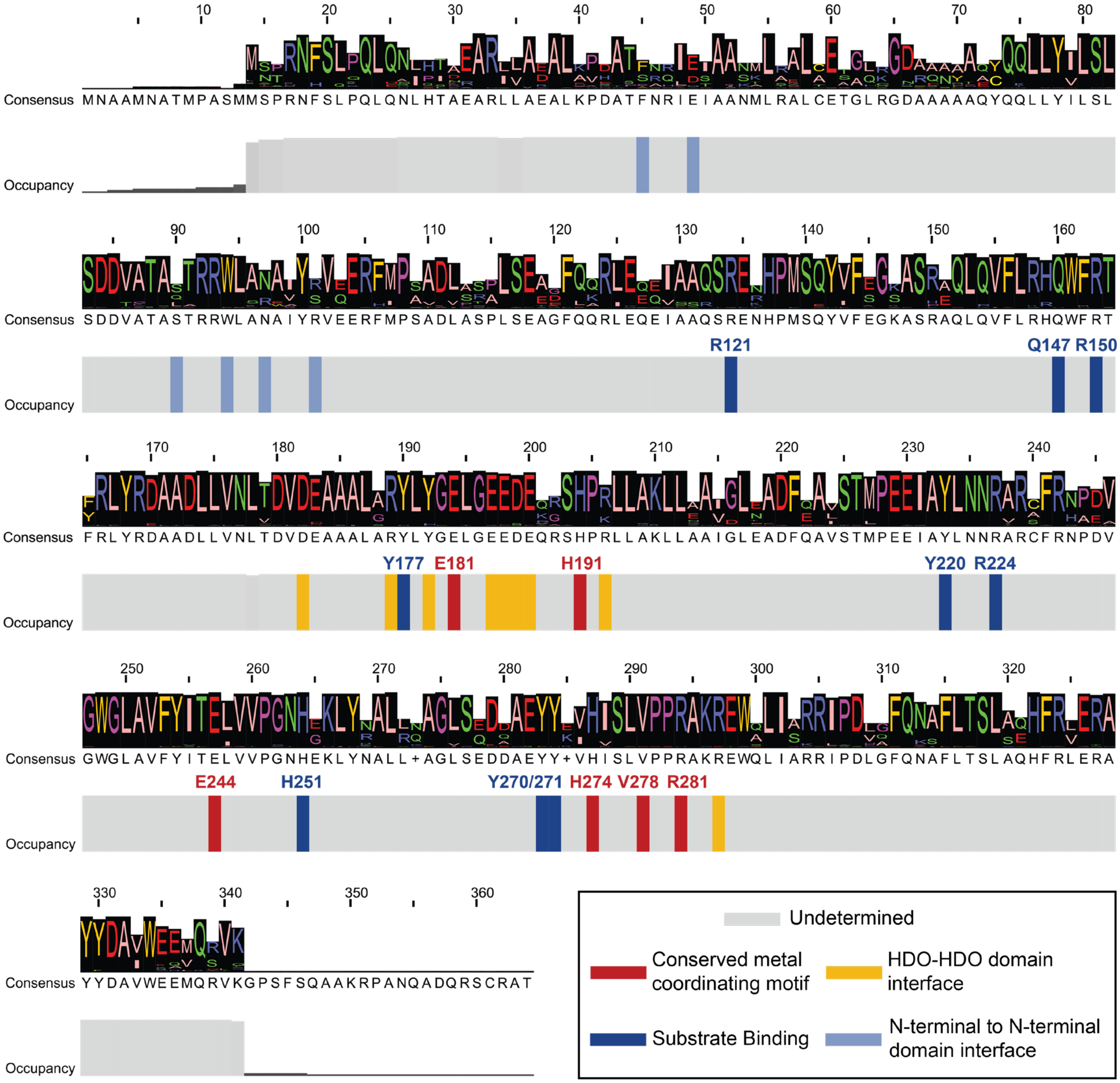


**Figure S61. Consensus sequence of the FlcD subgroup shows the iron- and substrate-binding residues are conserved.** A random member from each node of the FlcD subgroup was aligned using Clustal Omega (EMBL-EBI) and viewed in Jalview. The consensus logo is shown for each residue (black bars) and the occupancy is displayed for each residue. Colors of the occupancy bar indicate if the residue is aligned to the E-H-E-H-V-R motif of FlcD (red), substrate binding residues of FlcD (blue), or found at the HDO-HDO domain interface (yellow) or N-terminal domain interface (light blue) in the FlcD dimer.

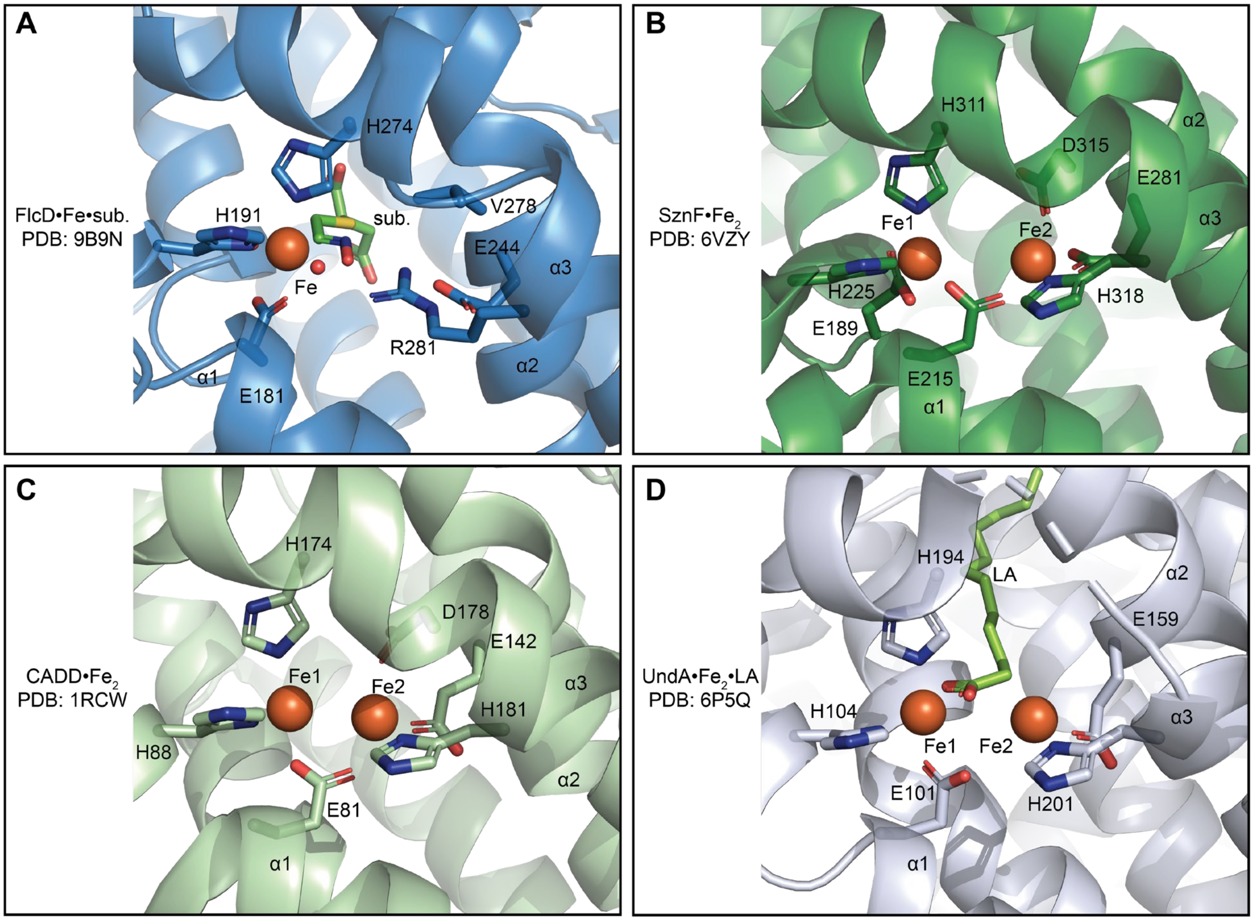


**Figure S62. Comparison of the active site of FlcD•Fe•substrate to other characterized HDOs.** Each iron-bound HDO structure is aligned to FlcD. Iron, orange. A) The active site of FlcD•Fe•substrate (PDB: 9B9N) monomer A, substrate in green. B) SznF•$Fe_2$ (PDB: 6VZY),[35] C) CADD•$Fe_2$ (PDB: 1RCW),[36] and D) UndA•$Fe_2$•LA (PDB: 6P5Q).[37] LA, lauric acid; sub., substrate (**2**). FlcD•Fe•substrate has a fully formed α3 helix in the presence of a single iron and substrate, with R281 sterically occupying the position where Fe2 could bind. Both SznF•$Fe_2$ and CADD•$Fe_2$ have a fully formed α3 helix in the presence of two irons, while UndA•$Fe_2$•LA contains some disorder in α3.

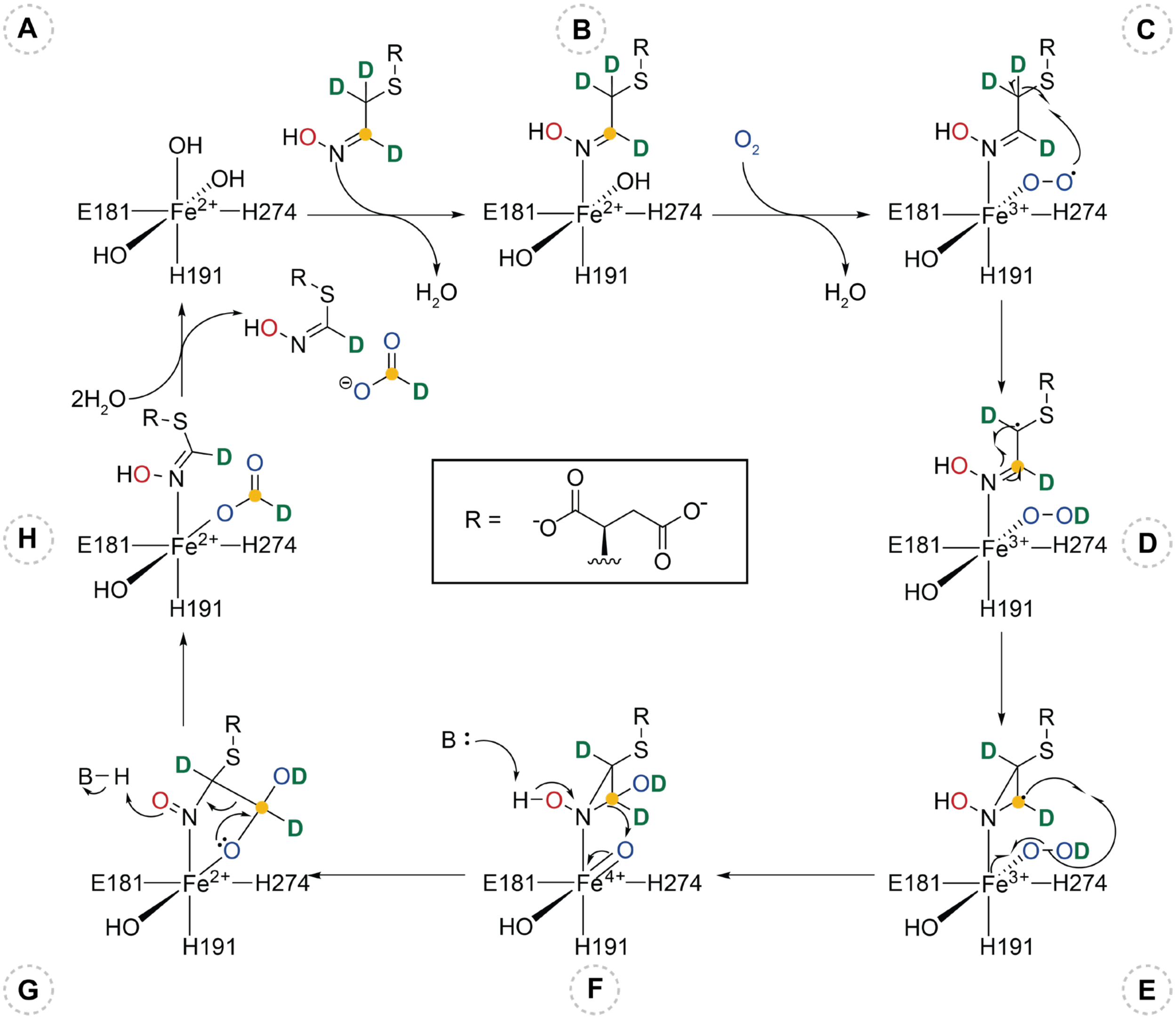


**Figure S63. Alternative proposed mechanism of FlcD.**

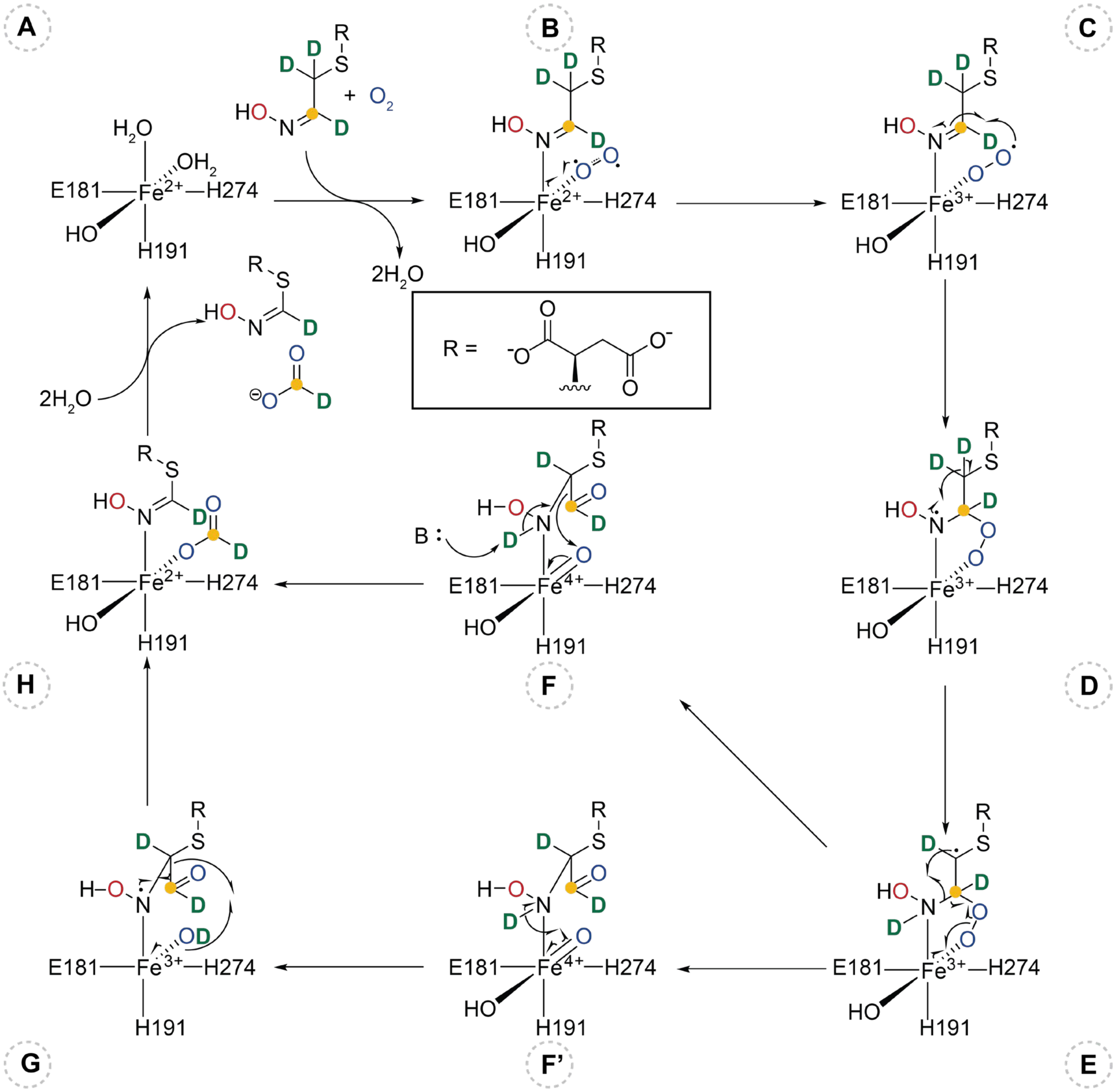


**Figure S64. Alternative proposed mechanism of FlcD.**

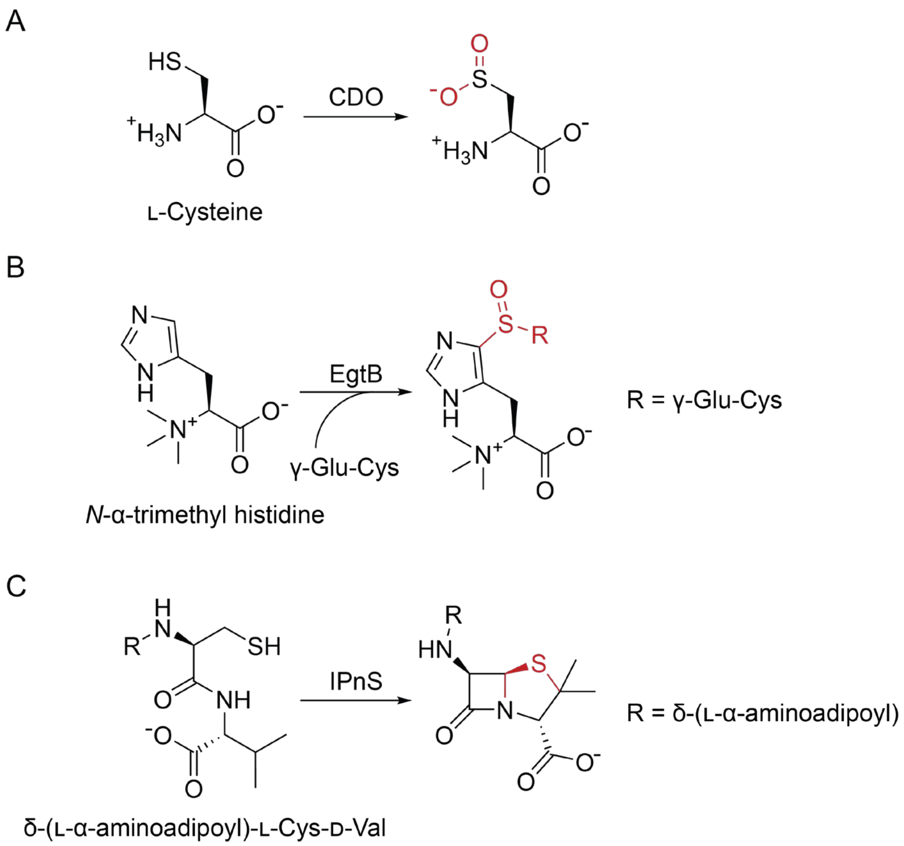


**Figure S65. Reactions catalyzed by other mononuclear non-heme iron-dependent oxidases.** A) Cysteine dioxygenase (CDO) oxidizes the cysteine thiol to a sulfinic acid.[39-42] B) EgtB oxidizes γ-Glu-Cys to a sulfoxide and couples the γ-Glu-Cys-sulfoxide to *N*-α-trimethyl histidine.[43-45] C) Isopenicillin *N* synthase (IPNS) catalyzes the formation of the thiazolidine and β-lactam rings of penicillins.[46-51] Transformations are highlighted in red.

## References

1. Patteson, J. B.; Putz, A. T.; Tao, L.; Simke, W. C.; Bryant III, L. H.; Britt, R. D.; Li, B., Biosynthesis of fluopsin C, a copper-containing antibiotic from *Pseudomonas aeruginosa*. *Science* **2021,** *374(6570),* 1005-1009.

2. Ortlund, E. A.; Lee, Y.; Solomon, I. H.; Hager, J. M.; Safi, R.; Choi, Y.; Guan, Z.; Tripathy, A.; Raetz, C. R. H.; McDonnell, D. P.; Moore, D. D.; Redinbo, M. R., Modulation of human nuclear receptor LRH-1 activity by phospholipids and SHP. *Nat. Struct. Mol. Bio.* **2005,** *12* (4), 357-363.

3. Grosse-Kunstleve, R. W.; Sauter, N. K.; Moriarty, N. W.; Adams, P. D., The Computational Crystallography Toolbox: crystallographic algorithms in a reusable software framework. *J. Appl. Crystallogr.* **2002,** *35* (1), 126-136.

4. Winn, M. D.; Ballard, C. C.; Cowtan, K. D.; Dodson, E. J.; Emsley, P.; Evans, P. R.; Keegan, R. M.; Krissinel, E. B.; Leslie, A. G. W.; McCoy, A.; McNicholas, S. J.; Murshudov, G. N.; Pannu, N. S.; Potterton, E. A.; Powell, H. R.; Read, R. J.; Vagin, A.; Wilson, K. S., Overview of the *CCP*4 suite and current developments. *Acta Crystallogr., Sect. D: Biol. Crystallogr.* **2011,** *67* (4), 235-242.
5. Winter, G.; McAuley, K. E., Automated data collection for macromolecular crystallography. *Methods* **2011,** *55* (1), 81-93.
6. Kabsch, W., *XDS*. *Acta Crystallogr., Sect. D: Biol. Crystallogr.* **2010,** *66* (2), 125-132.
7. Otwinowski, Z.; Minor, W., Processing of X-ray diffraction data collected in oscillation mode. In *Method. Enzymol.*, Academic Press: 1997; Vol. 276, pp 307-326.
8. Liebschner, D.; Afonine, P. V.; Baker, M. L.; Bunkóczi, G.; Chen, V. B.; Croll, T. I.; Hintze, B.; Hung, L.-W.; Jain, S.; McCoy, A. J.; Moriarty, N. W.; Oeffner, R. D.; Poon, B. K.; Prisant, M. G.; Read, R. J.; Richardson, J. S.; Richardson, D. C.; Sammito, M. D.; Sobolev, O. V.; Stockwell, D. H.; Terwilliger, T. C.; Urzhumtsev, A. G.; Videau, L. L.; Williams, C. J.; Adams, P. D., Macromolecular structure determination using X-rays, neutrons and electrons: recent developments in *Phenix*. *Acta Crystallogr. Sect. D: Struct. Biol.* **2019,** *75* (10), 861-877.
9. Casañal, A.; Lohkamp, B.; Emsley, P., Current developments in *Coot* for macromolecular model building of Electron Cryo-microscopy and Crystallographic Data. *Prot. Sci.* **2020,** *29* (4), 1055-1064.
10. Madeira, F.; Pearce, M.; Tivey, A. R. N.; Basutkar, P.; Lee, J.; Edbali, O.; Madhusoodanan, N.; Kolesnikov, A.; Lopez, R., Search and sequence analysis tools services from EMBL-EBI in 2022. *Nucleic Acids Res.* **2022,** *50* (W1), W276-W279.
11. Waterhouse, A. M.; Procter, J. B.; Martin, D. M. A.; Clamp, M.; Barton, G. J., Jalview Version 2—a multiple sequence alignment editor and analysis workbench. *Bioinformatics* **2009,** *25* (9), 1189-1191.
12. D.J., W., rentrez: an R package for the NCBI eUtils API. *The R Journal* **2017,** *9*, 520–526.
13. Team, R. C., R: A language and environment for statistical computing. *R Foundation for Statistical Computing, Vienna, Austria* **2021**.
14. Paysan-Lafosse, T.; Blum, M.; Chuguransky, S.; Grego, T.; Pinto, B. L.; Salazar, G. A.; Bileschi, M. L.; Bork, P.; Bridge, A.; Colwell, L.; Gough, J.; Haft, D. H.; Letunić, I.; Marchler-Bauer, A.; Mi, H.; Natale, D. A.; Orengo, C. A.; Pandurangan, A. P.; Rivoire, C.; Sigrist, C. J. A.; Sillitoe, I.; Thanki, N.; Thomas, P. D.; Tosatto, S. C. E.; Wu, C. H.; Bateman, A., InterPro in 2022. *Nucleic Acids Res.* **2023,** *51* (D1), D418-D427.
15. Magnusson, O. T.; Toyama, H.; Saeki, M.; Rojas, A.; Reed, J. C.; Liddington, R. C.; Klinman, J. P.; Schwarzenbacher, R., Quinone biogenesis: Structure and mechanism of PqqC, the final catalyst in the production of pyrroloquinoline quinone. *Proc. Natl. Acad. Sci. U. S. A.* **2004,** *101* (21), 7913-7918.
16. Bonnot, F.; Iavarone, A. T.; Klinman, J. P., Multistep, eight-electron oxidation catalyzed by the cofactorless oxidase, PqqC: Identification of chemical intermediates and their dependence on molecular oxygen. *Biochemistry* **2013,** *52* (27), 4667-4675.
17. Jenkins, A. H.; Schyns, G.; Potot, S.; Sun, G.; Begley, T. P., A new thiamin salvage pathway. *Nat. Chem. Bio.* **2007,** *3* (8), 492-497.
18. Ting, C. P., Funk, M. A., Halaby, S. L., Zhang, Z., Gonen, T., & van der Donk, W. A., Use of a scaffold peptide in the biosynthesis of amino acid–derived natural products. *Science* **2019,** *365* (6450), 280-284.
19. Blodgett, J. A.; Thomas, P. M.; Li, G.; Velasquez, J. E.; van der Donk, W. A.; Kelleher, N. L.; Metcalf, W. W., Unusual transformations in the biosynthesis of the antibiotic phosphinothricin tripeptide. *Nat. Chem. Bio.* **2007,** *3* (8), 480-485.
20. Cicchillo, R. M.; Zhang, H.; Blodgett, J. A.; Whitteck, J. T.; Li, G.; Nair, S. K.; van der Donk, W. A.; Metcalf, W. W., An unusual carbon-carbon bond cleavage reaction during phosphinothricin biosynthesis. *Nature* **2009,** *459* (7248), 871-874.

21. Zhu, H.; Peck, S. C.; Bonnot, F.; van der Donk, W. A.; Klinman, J. P., Oxygen-18 kinetic isotope effects of nonheme iron enzymes HEPD and MPnS support iron(III) superoxide as the hydrogen abstraction species. *J. Am. Chem. Soc.* **2015,** *137* (33), 10448-10451.
22. Ng, T. L.; Rohac, R.; Mitchell, A. J.; Boal, A. K.; Balskus, E. P., An N-nitrosating metalloenzyme constructs the pharmacophore of streptozotocin. *Nature* **2019,** *566* (7742), 94-99.
23. Rui, Z.; Li, X.; Zhu, X.; Liu, J.; Domigan, B.; Barr, I.; Cate, J. H.; Zhang, W., Microbial biosynthesis of medium-chain 1-alkenes by a nonheme iron oxidase. *Proc. Natl. Acad. Sci. U.S.A.* **2014,** *111* (51), 18237-18242.
24. Marchand, J. A.; Neugebauer, M. E.; Ing, M. C.; Lin, C. I.; Pelton, J. G.; Chang, M. C. Y., Discovery of a pathway for terminal-alkyne amino acid biosynthesis. *Nature* **2019,** *567* (7748), 420-424.
25. Breinlinger, S.; Phillips, T. J.; Haram, B. N.; Mareš, J.; Martínez Yerena, J. A.; Hrouzek, P.; Sobotka, R.; Henderson, W. M.; Schmieder, P.; Williams, S. M.; Lauderdale, J. D.; Wilde, H. D.; Gerrin, W.; Kust, A.; Washington, J. W.; Wagner, C.; Geier, B.; Liebeke, M.; Enke, H.; Niedermeyer, T. H. J.; Wilde, S. B., Hunting the eagle killer: A cyanobacterial neurotoxin causes vacuolar myelinopathy. *Science* **2021,** *371* (6536), eaax9050.
26. Macias-Orihuela, Y.; Cast, T.; Crawford, I.; Brandecker, K. J.; Thiaville, J. J.; Murzin, A. G.; Crécy-Lagard, V. d.; White, R. H.; Allen, K. D., An unusual route for *p*-aminobenzoate biosynthesis in *Chlamydia trachomatis* involves a probable self-sacrificing diiron oxygenase. *J. Bacteriol.* **2020,** *202* (20), e00319-20.
27. Wooldridge, R.; Stone, S.; Pedraza, A.; Ray, W. K.; Helm, R. F.; Allen, K. D., The *Chlamydia trachomatis p*-aminobenzoate synthase CADD is a manganese-dependent oxygenase that uses its own amino acid residues as substrates. *FEBS Lett.* **2023,** *597* (4), 557-572.
28. Manley, O. M.; Fan, R.; Guo, Y.; Makris, T. M., Oxidative decarboxylase UndA utilizes a dinuclear iron cofactor. *J. Am. Chem. Soc.* **2019,** *141* (22), 8684-8688.
29. McLaughlin, M. I.; Yu, Y.; van der Donk, W. A., Substrate recognition by the peptidyl-(*S*)-2-mercaptoglycine synthase TglHI during 3-thiaglutamate biosynthesis. *ACS Chem. Biol.* **2022,** *17* (4), 930-940.
30. Zheng, Y.; Xu, X.; Fu, X.; Zhou, X.; Dou, C.; Yu, Y.; Yan, W.; Yang, J.; Xiao, M.; van der Donk, W. A., Structures of the holoenzyme TglHI required for 3-thiaglutamate biosynthesis. *Structure* **2023,** *31* (10), 1220-1232.
31. Bunno, R.; Awakawa, T.; Mori, T.; Abe, I., Aziridine formation by a Fe(II)/alpha-ketoglutarate dependent oxygenase and 2-aminoisobutyrate biosynthesis in fungi. *Angew. Chem., Int. Ed.* **2021,** *60* (29), 15827-15831.
32. Ushimaru, R.; Abe, I., Unusual dioxygen-dependent reactions catalyzed by nonheme iron enzymes in natural product biosynthesis. *ACS Catal.* **2023,** *13* (2), 1045-1076.
33. Parkinson, E. I.; Lakkis, H. G.; Alwali, A. A.; Metcalf, M. E. M.; Modi, R.; Metcalf, W. W., An unusual oxidative rearrangement catalyzed by a divergent member of the 2-oxoglutarate-dependent dioxygenase superfamily during biosynthesis of dehydrofosmidomycin. *Angew. Chem., Int. Ed.* **2022,** *61* (30), e202206173.
34. McBride, M. J.; Nair, M. A.; Sil, D.; Slater, J. W.; Neugebauer, M. E.; Chang, M. C. Y.; Boal, A. K.; Krebs, C.; Bollinger, J. M., Jr., Substrate-triggered mu-peroxodiiron(III) intermediate in the 4-chloro-l-Lysine-fragmenting heme-oxygenase-like diiron oxidase (HDO) BesC: Substrate dissociation from, and C4 targeting by, the intermediate. *Biochemistry* **2022,** *61* (8), 689-702.
35. McBride, M. J.; Pope, S. R.; Hu, K.; Okafor, C. D.; Balskus, E. P.; Bollinger, J. M., Jr.; Boal, A. K., Structure and assembly of the diiron cofactor in the heme-oxygenase-like domain of the N-nitrosourea-producing enzyme SznF. *Proc. Natl. Acad. Sci. U. S. A.* **2021,** *118* (4), e201593118.
36. Schwarzenbacher, R.; Stenner-Liewen, F.; Liewen, H.; Robinson, H.; Yuan, H.; Bossy-Wetzel, E.; Reed, J. C.; Liddington, R. C., Structure of the *Chlamydia* protein CADD reveals a redox enzyme that modulates host cell apoptosis. *J. Biol. Chem.* **2004,** *279* (28), 29320-29324.

37. Zhang, B.; Rajakovich, L. J.; Van Cura, D.; Blaesi, E. J.; Mitchell, A. J.; Tysoe, C. R.; Zhu, X.; Streit, B. R.; Rui, Z.; Zhang, W.; Boal, A. K.; Krebs, C.; Bollinger, J. M., Jr., Substrate-triggered formation of a peroxo-Fe(2)(III/III) intermediate during fatty acid decarboxylation by UndA. *J. Am. Chem. Soc.* **2019,** *141* (37), 14510-14514.
38. Baugh, L.; Phan, I.; Begley, D. W.; Clifton, M. C.; Armour, B.; Dranow, D. M.; Taylor, B. M.; Muruthi, M. M.; Abendroth, J.; Fairman, J. W.; Fox, D.; Dieterich, S. H.; Staker, B. L.; Gardberg, A. S.; Choi, R.; Hewitt, S. N.; Napuli, A. J.; Myers, J.; Barrett, L. K.; Zhang, Y.; Ferrell, M.; Mundt, E.; Thompkins, K.; Tran, N.; Lyons-Abbott, S.; Abramov, A.; Sekar, A.; Serbzhinskiy, D.; Lorimer, D.; Buchko, G. W.; Stacy, R.; Stewart, L. J.; Edwards, T. E.; Van Voorhis, W. C.; Myler, P. J., Increasing the structural coverage of tuberculosis drug targets. *Tuberculosis* **2015,** *95* (2), 142-148.
39. Sörbo, B.; Ewetz, L., The enzymatic oxidation of cysteine to cysteinesulfinate in rat liver. *Biochem. Biophys. Res. Commun.* **1965,** *18* (3), 359-363.
40. Lombardini, J. B.; Singer, T. P.; Boyer, P. D., Cysteine oxygenase: II. Studies on the mechanism of the reaction with $^{18}$oxygen. *J. Biol. Chem.* **1969,** *244* (5), 1172-1175.
41. Chai, S. C.; Jerkins, A. A.; Banik, J. J.; Shalev, I.; Pinkham, J. L.; Uden, P. C.; Maroney, M. J., Heterologous expression, purification, and characterization of recombinant rat cysteine dioxygenase. *J. Biol. Chem.* **2005,** *280* (11), 9865-9869.
42. McCoy, J. G.; Bailey, L. J.; Bitto, E.; Bingman, C. A.; Aceti, D. J.; Fox, B. G.; Phillips, G. N., Structure and mechanism of mouse cysteine dioxygenase. *Proc. Natl. Acad. Sci. U. S. A.* **2006,** *103* (9), 3084-3089.
43. Ishikawa, Y.; Israel, S. E.; Melville, D. B., Participation of an intermediate sulfoxide in the enzymatic thiolation of the imidazole ring of hercynine to form ergothioneine. *J. Biol. Chem.* **1974,** *249* (14), 4420-4427.
44. Seebeck, F. P., In vitro reconstitution of mycobacterial ergothioneine biosynthesis. *J. Am. Chem. Soc.* **2010,** *132* (19), 6632-6633.
45. Goncharenko, K. V.; Vit, A.; Blankenfeldt, W.; Seebeck, F. P., Structure of the sulfoxide synthase EgtB from the ergothioneine biosynthetic pathway. *Angew. Chem., Int. Ed.* **2015,** *54* (9), 2821-2824.
46. Arnstein, H. R. V.; Clubb, M. E., The biosynthesis of penicillin. 8. Investigation of cyclic cysteinylvaline peptides as precursors. *Biochem. J.* **1958,** *68* (3), 528-535.
47. Pang, C. P.; Chakravarti, B.; Adlington, R. M.; Ting, H. H.; White, R. L.; Jayatilake, G. S.; Baldwin, J. E.; Abraham, E. P., Purification of isopenicillin N synthetase. *Biochem. J.* **1984,** *222* (3), 789-795.
48. Hollander, I. J.; Shen, Y.-Q.; Heim, J.; Demain, A. L.; Wolfe, S., A pure enzyme catalyzing penicillin biosynthesis. *Science* **1984,** *224* (4649), 610-612.
49. Samson, S. M.; Belagaje, R.; Blankenship, D. T.; Chapman, J. L.; Perry, D.; Skatrud, P. L.; Vanfrank, R. M.; Abraham, E. P.; Baldwin, J. E.; Queener, S. W.; Ingolia, T. D., Isolation, sequence determination and expression in *Escherichia coli* of the isopenicillin N synthetase gene from *Cephalosporium acremonium*. *Nature* **1985,** *318* (6042), 191-194.
50. Roach, P. L.; Clifton, I. J.; Hensgens, C. M. H.; Shibata, N.; Schofield, C. J.; Hajdu, J.; Baldwin, J. E., Structure of isopenicillin N synthase complexed with substrate and the mechanism of penicillin formation. *Nature* **1997,** *387* (6635), 827-830.
51. Burzlaff, N. I.; Rutledge, P. J.; Clifton, I. J.; Hensgens, C. M.; Pickford, M.; Adlington, R. M.; Roach, P. L.; Baldwin, J. E., The reaction cycle of isopenicillin N synthase observed by X-ray diffraction. *Nature* **1999,** *401* (6754), 721-724.
